# Role of connexin 43 in hyperoxia-induced bronchopulmonary dysplasia and associated pulmonary hypertension

**DOI:** 10.1371/journal.pone.0356364

**Published:** 2026-08-24

**Authors:** Claire-Marie Pilard, Laure Gassiat, Guillaume Cardouat, Isabel Gauthereau, Paul Robillard, Eric Dumas-de-la-Roque, Fanny Sauvestre, Fanny Pelluard, Sophie Berenguer, Melie Sarreau, Loïc Sentilhes, Fréderic Coatleven, Marie Vincienne, Roger Marthan, Patrick Berger, Véronique Freund-Michel, Christelle Guibert

**Affiliations:** 1 Univ. Bordeaux, INSERM, CRCTB, U 1045, Pessac, France; 2 Bordeaux-Bagatelle Protestant Health Centre, Maternity Department, Talence, France; 3 CHU de Bordeaux, Neonatology Department, Bordeaux, France; 4 CHU de Bordeaux, Pathology Department, Bordeaux, France; 5 CHU de Bordeaux, Obstetrics and Gynecology Department, Bordeaux, France; 6 CHU de Bordeaux, Respiratory Function Testing Department, Bordeaux, France; Royal College of Surgeons in Ireland, IRELAND

## Abstract

Premature infants frequently require oxygen supplementation due to lung immaturity, exposing them to the risk of bronchopulmonary dysplasia (BPD), a chronic lung disease characterized by arrested alveolar growth and inflammation. Connexin 43-dependent gap junctions (Cx43-GJ) regulate intercellular communication during lung development and inflammatory responses, but their involvement in BPD remains incompletely defined. This study aimed to assess the contribution of Cx43-GJ in experimental BPD and to evaluate whether selective pharmacological inhibition of Cx43 modulates hyperoxia-induced lung injury and pulmonary hypertension. Newborn rats were exposed to normoxia or hyperoxia (90% O_2_) for 14 days and treated daily with the Cx43-GJ inhibitor ^43^Gap26 or vehicle. Human fetal pulmonary artery smooth muscle cells (HfPA-SMC) were exposed to 21% O_2_ or 60% O_2_ and treated with or without ^43^Gap26 for 48 hours. Lung morphology, function, surfactant protein expression, extracellular matrix markers, macrophage phenotype, cytokine secretion, and oxidative stress markers were assessed. *In vivo*, ^43^Gap26 reduced hyperoxia-induced Cx43 overexpression and partially improved alveolarization. However, ^43^Gap26 failed to prevent alterations in lung function, decreased surfactant protein-B expression, extracellular matrix remodeling, macrophage M2 polarization, increased tissue inhibitor of metalloproteinase-1 secretion, pulmonary hypertension, or mortality. Inhibition of Cx43 was also associated with reduced markers of type II alveolar epithelial cell expression. In HfPA-SMC, ^43^Gap26 did not modify hyperoxia-induced secretion of pro-inflammatory cytokines, despite increased Cx43 expression. Classical markers of oxidative damage were not significantly increased under our experimental conditions, although heme oxygenase-1 expression was elevated. Overall, pharmacological inhibition of Cx43 partially restored alveolar structure but did not prevent major pathological features of experimental BPD or pulmonary hypertension. These findings suggest that Cx43-GJ signaling is involved in the regulation of alveolar structure during hyperoxic lung injury, but its pharmacological inhibition alone is insufficient to prevent the complex structural and vascular alterations characteristic of experimental BPD.

## Introduction

Bronchopulmonary dysplasia (BPD) was first described in 1967 by Northway *et al*. [1], but it continues to be one of the most common complications of prematurity, despite major advances in neonatology over the last couple of decades. Indeed, improved medical management of extreme prematurity (before 28 weeks of amenorrhea) increases survival rates but also the incidence of BPD, which varies between 20–75% depending on the cohort [2]. BPD is a clinical consequence of prenatal (intra-uterine growth restriction, smoking, chorioamnionitis) and post-natal (oxygen supplementation, mechanical ventilation, sepsis) lung injuries. Indeed, preterm infants are born during the second phase of lung development, which includes the canalicular, saccular and alveolar stages. During these stages, the lung is not yet efficient at gas exchange. Therefore, assisted ventilation and supplemental oxygen will initially support ventilation after birth, but will secondarily lead to severe intrapulmonary inflammation characterized by (i) an increase in pulmonary macrophage infiltration, (ii) an increase in pro-inflammatory cytokines secretion such as interleukin-6 (IL-6) and (iii) an imbalanced M1/M2 macrophage polarization [3–5]. These pro-inflammatory features hurdle to normal pulmonary growth and repair, and are responsible for blunted alveolarization. Abnormal microvascular development also occurs, including pulmonary artery (PA) wall thickening and vascular growth arrest responsible for pulmonary microvasculature rarefaction, leading to pulmonary hypertension associated with BPD (PH-BPD) [6,7]. PH-BPD is a severe complication of BPD affecting nearly 25% of extreme premature infants with moderate to severe BPD [8] and leading to a mortality rate up to 50% within 2 years of diagnosis [6,8,9]. Anti-inflammatory approaches attenuate impaired alveolarization induced by hyperoxia [5]. However, the functional contribution of inflammatory cells to arrested alveolarization and disturbed vascular development remains largely unclear. Moreover, nowadays, our understanding of normal and aberrant lung development is still poor, and there is an urgent need to discover new pathways involved in alveolar development that could reveal new therapeutic targets to prevent abnormal lung development or promote lung regeneration and repair.

Connexin 43 (Cx43), a gap junction (GJ) protein involved in cell-to-cell communication seems to be a new potential key player implicated both in late lung development and in the regulation of the inflammatory response. Indeed, Cx43 is expressed in the mouse embryo from the day of gestation E14.5 and in the adult type I and II alveolar epithelial cells (ATI and II, respectively), and also in pulmonary endothelium, lung macrophages and smooth muscle cells [10]. Cx43 knockout newborn mice have hypoplastic lungs with narrow airspaces and thicker interalveolar septae, and die rapidly after birth from severe respiratory failure [11]. In another inflammatory lung disease model of acute respiratory distress syndrome induced by intratracheal instillation of *Pseudomonas aeruginosa* lipopolysaccharide (LPS), Cx43 knockdown mice (Cx43^+/-^ mice) have decreased neutrophils in the bronchoalveolar lavage (BAL) fluid compared to wild-type mice, suggesting that Cx43 may play a role in pro-inflammatory cells recruitment into the lung [12]. In the same way, Cx43 is known to be upregulated in a model of ovalbumin-induced allergic asthma, and its inhibition with the blocking peptide ^43^Gap26 reduced airway hyper-responsiveness and eosinophil infiltration [13]. At last, Qing *et al*. showed that hyperoxia increased Cx43 expression, apoptosis and reactive oxygen species (ROS) production in lungs from a neonatal model of BPD induced by hyperoxia exposure, and they further demonstrated that inhibition of Cx43 with ^43^Gap26 reversed these changes, thus improving alveolarization [14]. Regarding the role of Cx43 in pulmonary hypertension (PH), a recent study from our laboratory showed that Cx43 expression was increased in pulmonary artery (PA) from patients with chronic-hypoxia-induced PH (CH-PH), and Cx43^+/-^ mice exposed to chronic hypoxia were partially protected against CH-PH with decreased PA remodeling and inflammatory cell infiltration [15]. However, the role of Cx43 in BPD and PH-BPD pathogenesis remains unclear. In this work, we hypothesized that Cx43 plays a key role in the arrested alveolarization and disturbed vascular development associated with BPD and PH-BPD, by regulating macrophage-driven inflammatory response. To test our hypothesis, we used a well-known neonatal murine model of BPD induced by chronic exposure to hyperoxia (90% O_2_) for 14 days, with rat pups treated or not with a selective Cx43 inhibitor, ^43^Gap26. Some experiments were also performed in human fetal pulmonary artery smooth muscle cells (HfPA-SMC) exposed to hyperoxia (60% O_2_) and treated or not with ^43^Gap26 for 2 days, in order to examine some mechanistic underpinnings of Cx43 on inflammation and oxidative stress.

## Methods

Detailed methods are available in supporting information.

### Study design

We employed a cross-sectional design combining *in vivo* investigations in a well-established neonatal rat model of hyperoxia with in vitro analyses using a novel culture system of human fetal pulmonary artery smooth muscle cells (HfPA-SMC) exposed to hyperoxic conditions (S1 Fig). The study first examined the effects of the connexin 43 (Cx43) inhibitory peptide ^43^Gap26 (GenScript Biotech, Rijswijk, NL) on hallmark features of bronchopulmonary dysplasia (BPD)—including alveolar development, lung function, extracellular matrix (ECM) composition, pulmonary inflammation, and oxidative stress—as well as on parameters of pulmonary hypertension (PH) such as pulmonary artery (PA) pressure, right ventricular (RV) hypertrophy, vascular remodeling, reactivity, and density. Selected *in vivo* findings were then validated *in vitro* by assessing Cx43 expression, inflammatory response, and oxidative stress in HfPA-SMC.

PH development was confirmed through *in vivo* hemodynamic assessment by echocardiography and cardiac catheterization. RV hypertrophy was quantified by separating the right ventricle (RV) from the left ventricle (LV) and septum and calculating the Fulton index (RV/[LV + septum] weight ratio). Pulmonary function was measured using the forced oscillation technique. Lung morphometry (alveolarization), vascular remodeling and density, and macrophage infiltration were evaluated by histological staining. Inflammatory markers were quantified by ELISA in both experimental models, while PA reactivity was assessed by myography. Oxidative stress was analyzed by Western blotting. All primary antibodies used for Western blotting and immunostaining experiments are provided in S1 Table.

All animal experiments complied with the Guide for the Care and Use of Laboratory Animals (National Institutes of Health) and were approved by the local ethics committee (CEEA 50) and French regulatory authorities (protocol no. APAFIS#28597). Animals were randomly assigned to experimental groups, and data from replicate cohorts were pooled. Unless otherwise stated in figure legends, each experiment included at least five animals.

The establishment of a human fetal tissue bank (cardiac and pulmonary) and all related procedures were approved by the French Biomedicine Agency (authorization no. PFS15−004, effective since May 13, 2015, with no expiration). Written informed consent was obtained from each donor prior to elective pregnancy termination. Ethical approval for this study was granted by the Research Ethics Committee of Bordeaux University Hospital, and all procedures adhered to the principles of the Declaration of Helsinki. In vitro experiments were conducted using cells from at least five independent donors.

### Statistical analysis

Data are presented as individual values and means ± SEM. The sample size (n) represents the number of independent biological replicates—animals, donors, or HfPA-SMC measurements—as specified in figure legends. Data normality was assessed using the Shapiro–Wilk test. Concentration–response relationships were compared using two-way ANOVA. For comparisons among more than two groups, one-way ANOVA or Kruskal–Wallis tests were applied depending on data distribution, followed by appropriate post hoc analyses. Differences between two groups were evaluated using Student’s t-test. Survival curves were analyzed using the Log-rank test. Statistical analyses were performed using GraphPad Prism (version 9.0.0, GraphPad Software, San Diego, CA, USA). A p-value < 0.05 was considered statistically significant.

## Results

### Effect of hyperoxia and Cx43 inhibition on Cx43 expression in both models

**Fig 1:**
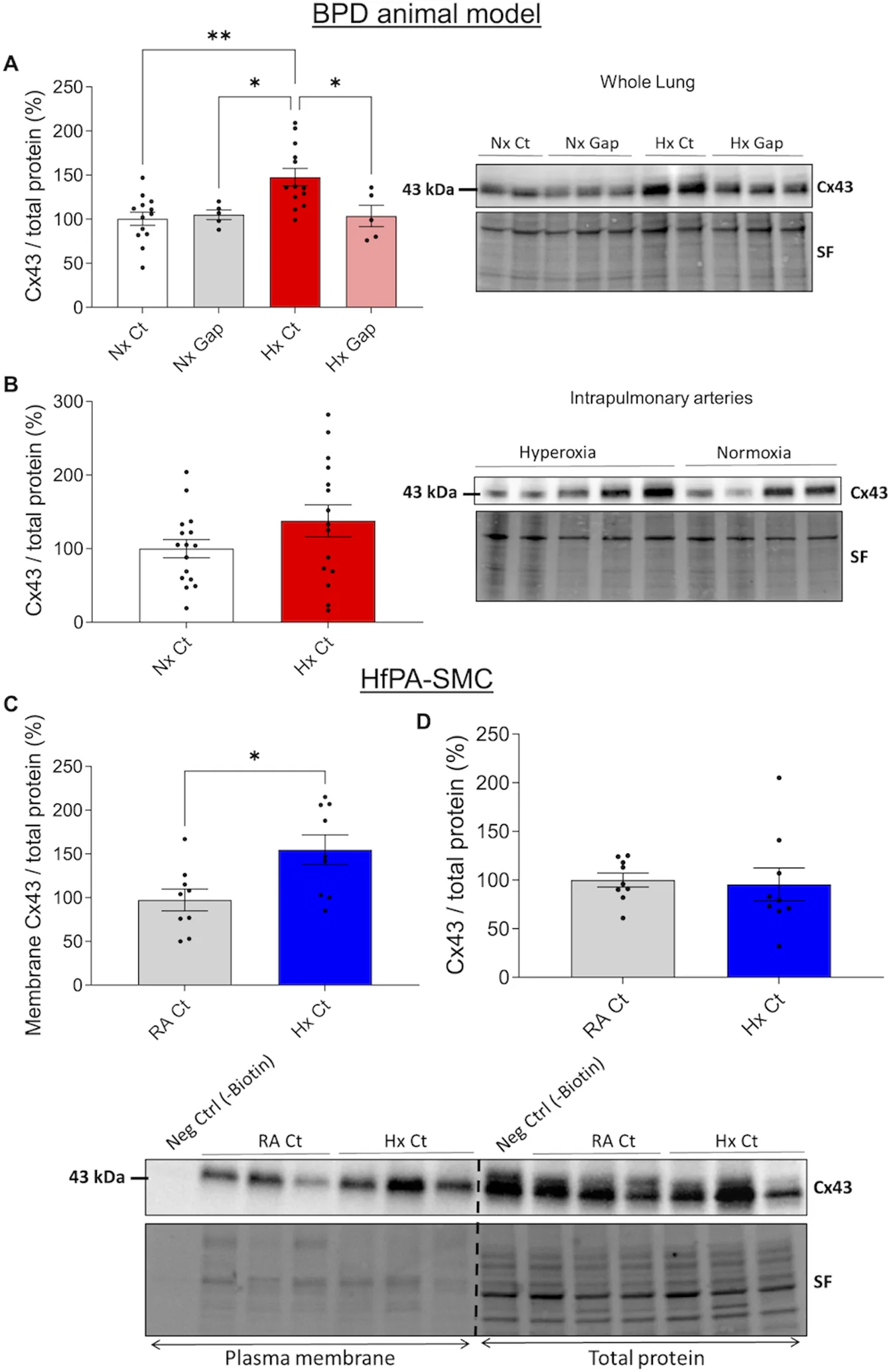
Effect of hyperoxia and Cx43 inhibition on Cx43 expression in animal and human models. (A) Cx43 protein expression was analyzed by Western blot in whole lung homogenates from neonatal rats exposed to normoxia (Nx Ct) or hyperoxia (Hx Ct), and daily treated or not with the Cx43 inhibitor ^43^Gap26 (Nx Gap or Hx Gap) for 14 days. Representative immunoblots are shown (n = 5–13 per group). Protein levels were normalized to total protein using stain-free (SF) technology and expressed as a percentage of Nx Ct. Data are presented as mean ± SEM. *P < 0.05, **P < 0.01, one-way ANOVA followed by Tukey’s post hoc test. (B) Cx43 expression in intrapulmonary arteries from neonatal rats exposed to normoxia (Nx Ct) or hyperoxia (Hx Ct). Representative immunoblots are shown (n = 15–16 per group). Protein levels were normalized to total protein (SF technology) and expressed as a percentage of Nx Ct. Data are presented as mean ± SEM. Unpaired t-test. (C) Cx43 biotinylated membrane fractions obtained from human fetal pulmonary artery smooth muscle cells (HfPA-SMCs) exposed to room air (RA Ct – 21% O_2_) or hyperoxia (Hx Ct). Cell-surface proteins were labeled using a membrane-impermeable biotin reagent at 4 °C to restrict labeling to extracellularly exposed proteins, followed by streptavidin pull-down. Neg Ctrl (–Biotin) corresponds to samples processed in parallel without biotin labeling and was included to assess non-specific binding to streptavidin beads. Representative immunoblots are shown (left side) (n = 9 donors per group). Protein levels were normalized to total protein (SF technology) and expressed as a percentage of RA Ct. Data are presented as mean ± SEM. *P < 0.05, paired t-test. (D) Total Cx43 protein expression in HfPA-SMC exposed to RA (RA Ct) or hyperoxia (Hx Ct). Representative immunoblots are shown (right side) (n = 9 donors per group). Protein levels were normalized to total protein (SF technology) and expressed as a percentage of RA Ct. Data are presented as mean ± SEM. Paired t-test.

We firstly showed that hyperoxia induced an increase in Cx43 expression *in vivo* in rat pups whole lungs (p = 0.0014) but not in their isolated intrapulmonary arteries (Fig 1A and 1B, respectively). Furthermore, in whole lungs from animals exposed to hyperoxia and treated with ^43^Gap26 (Hx Gap), Cx43 inhibition was associated with a significant decrease in Cx43 expression as compared to the hyperoxic group (Hx Ct – p = 0.0353) (Fig 1A).

*In vitro* in HfPA-SMC exposed to hyperoxia, we observed an increase in membrane Cx43 expression (p = 0.0466) (Fig 1C) while total Cx43 protein expression remained unchanged (Fig 1D).

### Effect of Cx43 inhibition on alveolarization and lung function in the BPD animal model

**Fig 2:**
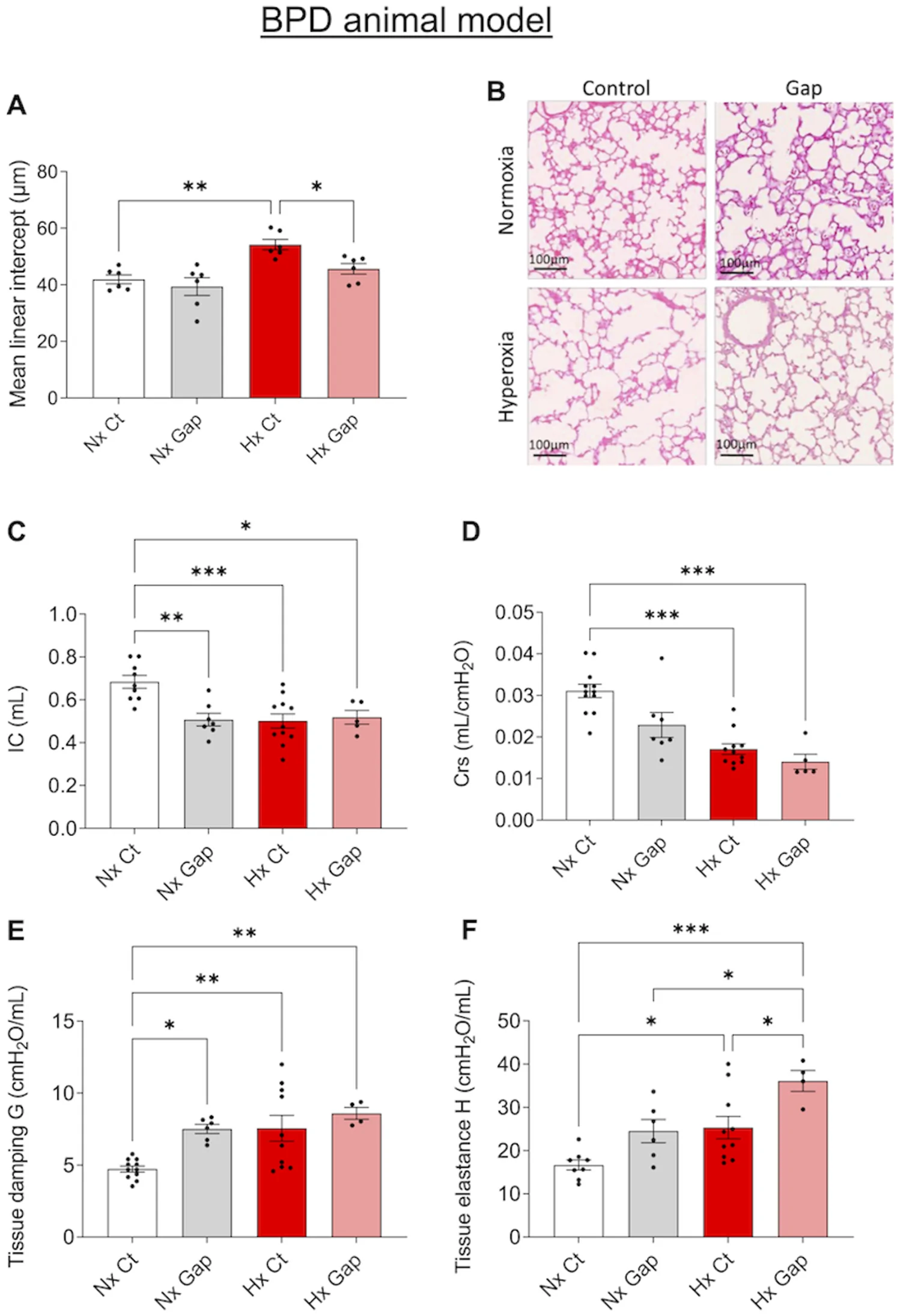
Effect of Cx43 inhibition on alveolarization and lung function in a rat model of bronchopulmonary dysplasia. (A) Mean linear intercept (MLI) measured at postnatal day 14 in neonatal rats exposed to normoxia (Nx Ct) or hyperoxia (Hx Ct), and daily treated or not with the Cx43 inhibitor ^43^Gap26 (Nx Gap or Hx Gap). n = 6 rats per group. Data are presented as mean ± SEM. *P < 0.05, **P < 0.01; one-way ANOVA followed by Tukey’s post hoc test. (B) Representative hematoxylin and eosin-stained lung sections from the same experimental groups as in (A). Images shown are representative of n = 6 animals per group. (C–F) Lung function parameters measured in intubated neonatal rats from the same experimental groups as in (A). Parameters include inspiratory capacity (IC, C), respiratory system compliance (Crs, D), tissue damping G (E), and tissue elastance H (F). n = 4–12 animals per group. Data are presented as mean ± SEM. *P < 0.05, **P < 0.01, ***P < 0.001; one-way ANOVA followed by Tukey’s post hoc test (C, E, F) or Dunn’s multiple comparisons test (D).

We then assessed the effects of Cx43 inhibition by ^43^Gap26 on the main characteristics of BPD. Regarding alveolarization, we demonstrated that ^43^Gap26 treatment significantly prevented hyperoxia-induced hypoalveolarization (p = 0.0465) (Fig 2A and 2B).

However, when studying pulmonary function by forced oscillation maneuver *in vivo*, hyperoxia exposure significantly decreased the inspiratory capacity (IC) and Crs (p < 0.001) (Fig 2C and 2D), and increased tissue damping G (p = 0.0049) (reflecting increased air resistance in the alveoli), and tissue elastance H (p = 0.0382) (reflecting an increased stiffness of the lung tissue) (Fig 2E and 2F). ^43^Gap26 treatment did not improve hyperoxia-induced IC, Crs and tissue damping G alteration (Fig 2C, 2D and 2E) whereas it worsened tissue elastance H in hyperoxic condition (p = 0.0382) (Fig 2F). Moreover, ^43^Gap26 treatment in animals maintained in normoxia (Nx Gap) significantly altered IC (p = 0.0032) and tissue damping G (p = 0.0197) compared to the normoxic control group (Fig 2C and 2E).

### Effect of Cx43 inhibition by ^43^Gap26 on the expression of both alveolar epithelial cells (ATI and II) markers and the surfactant protein B (SP-B) in the BPD animal model

**Fig 3:**
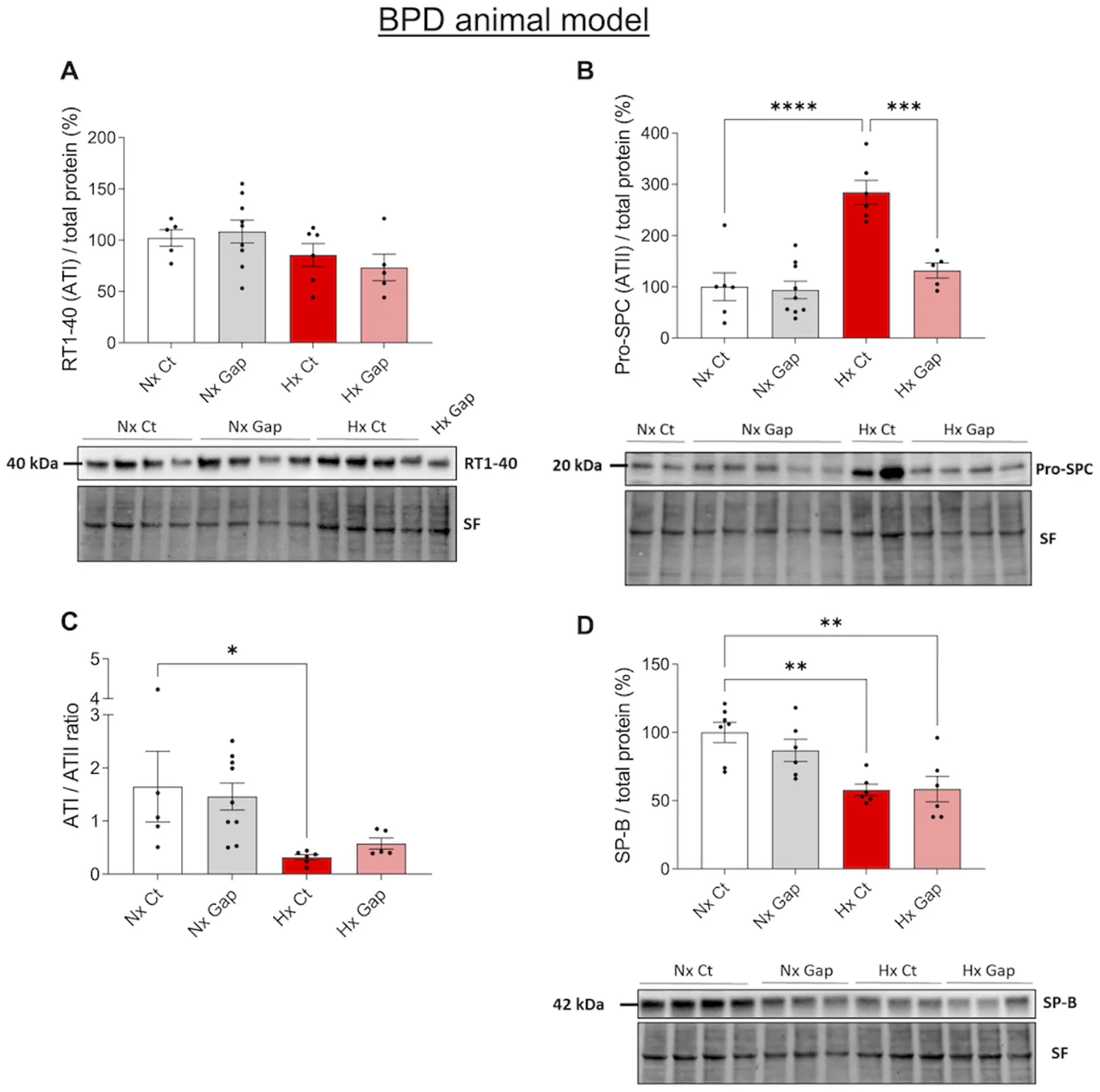
Effect of Cx43 inhibition on alveolar epithelial cell markers and surfactant protein B expression in a rat model of bronchopulmonary dysplasia. (A) Expression of RT1-40 glycoprotein (podoplanin), a specific marker of alveolar type I (ATI) epithelial cells, analyzed by Western blot in whole lung homogenates from neonatal rats exposed to normoxia (Nx Ct) or hyperoxia (Hx Ct), and daily treated or not with the Cx43 inhibitor ^43^Gap26 (Nx Gap or Hx Gap). Representative immunoblots are shown (n = 5–9 per group). Protein levels were normalized to total protein using stain-free (SF) technology and expressed as a percentage of Nx Ct. Data are presented as mean ± SEM. One-way ANOVA followed by Tukey’s post hoc test. (B) Expression of pro-surfactant protein C (pro-SP-C), a specific marker of alveolar type II (ATII) epithelial cells, assessed by Western blot in whole lungs from the same experimental groups as in (A). Representative immunoblots are shown (n = 5–9 per group). Protein levels were normalized to total protein (SF technology) and expressed as a percentage of Nx Ct. Data are presented as mean ± SEM. ***P < 0.001, ****P < 0.0001; one-way ANOVA followed by Tukey’s post hoc test. (C) ATI/ATII marker expression ratio calculated from data in (A) and (B). This ratio reflects relative protein abundance and should be interpreted as an indirect indicator of ATI and ATII representation rather than a direct enumeration of ATI and ATII cells. Data are presented as mean ± SEM. *P < 0.05; one-way ANOVA followed by Dunn’s multiple comparisons test. (D) Expression of surfactant protein B (SP-B) in whole lung homogenates from the same experimental groups as in (A). Representative immunoblots are shown (n = 6–7 per group). Protein levels were normalized to total protein (SF technology) and expressed as a percentage of Nx Ct. Data are presented as mean ± SEM. **P < 0.01; one-way ANOVA followed by Tukey’s post hoc test.

Then, we focused on ATI and ATII cells and we showed that neither hyperoxia nor ^43^Gap26 had any effect on ATI cell marker expression (Fig 3A). Regarding ATII cell marker expression, we demonstrated that hyperoxia significantly increased ATII cell marker expression (p < 0.0001) (Fig 3B), leading to a significant reduction in the ATI (RT1–40) to ATII (pro-SPC) cell marker ratio (p = 0.0141) (Fig 3C). Moreover, ^43^Gap26 prevented the hyperoxia-induced increase in ATII cell marker expression as compared to the hyperoxic control group (p = 0.006) (Fig 3B).

We also studied surfactant protein B (SP-B) expression synthesized by ATII cells. We showed that although hyperoxia increased ATII cell marker expression, it did significantly decrease the expression of SP-B as compared to the normoxic control group (p = 0.0032) and ^43^Gap26 did not prevent this decrease (Fig 3D).

### Effect of Cx43 inhibition by ^43^Gap26 on macrophages infiltration in the lung in the BPD animal model and on the pro-inflammatory marker expression in both animal and human models

**Fig 4:**
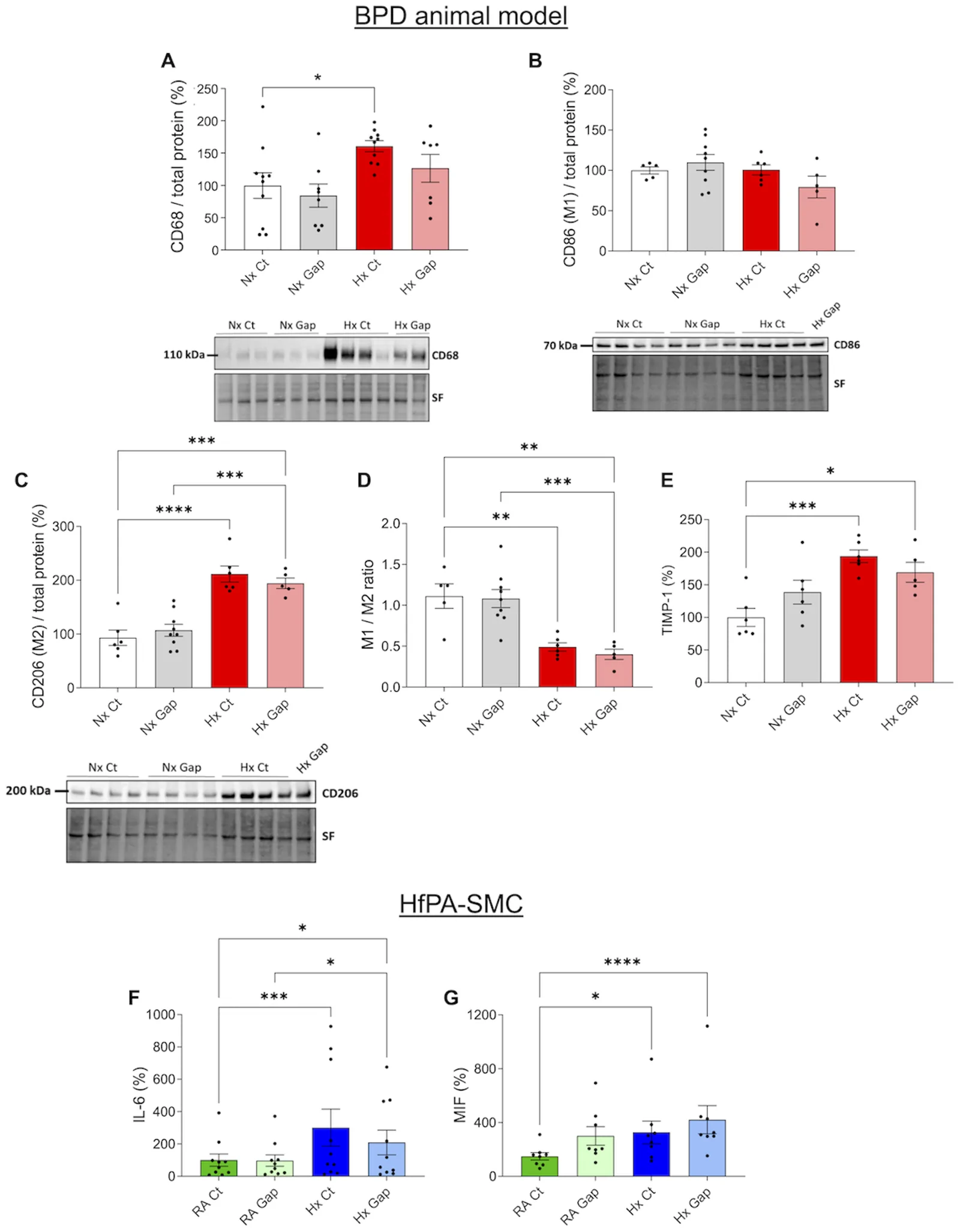
Effect of Cx43 inhibition on hyperoxia-induced inflammation in a rat model of bronchopulmonary dysplasia. (A) Expression of CD68, a pan-macrophage marker, analyzed by Western blot in whole lung homogenates from neonatal rats exposed to normoxia (Nx Ct) or hyperoxia (Hx Ct), and daily treated or not with the Cx43 inhibitor ^43^Gap26 (Nx Gap or Hx Gap). Representative immunoblots are shown (n = 7–10 per group). Protein levels were normalized to total protein using stain-free (SF) technology and expressed as a percentage of Nx Ct. Data are presented as mean ± SEM. *P < 0.05; one-way ANOVA followed by Tukey’s post hoc test. (B) Expression of CD86, a marker of classically activated (M1) macrophages, assessed by Western blot in the same experimental groups as in (A). Representative immunoblots are shown (n = 5–9 per group). Protein levels were normalized to total protein (SF technology) and expressed as a percentage of Nx Ct. Data are presented as mean ± SEM; one-way ANOVA followed by Sidak’s post hoc test. (C) Expression of CD206, a marker of alternatively activated (M2) macrophages, assessed by Western blot in the same experimental groups as in (A). Representative immunoblots are shown (n = 5–9 per group). Protein levels were normalized to total protein (SF technology) and expressed as a percentage of Nx Ct. Data are presented as mean ± SEM. ***P < 0.001, ****P < 0.0001; one-way ANOVA followed by Tukey’s post hoc test. (D) M1/M2 ratio calculated from CD86 and CD206 protein expression levels in the same experimental groups as in (A). Data are presented as mean ± SEM. **P < 0.01, ***P < 0.001; one-way ANOVA followed by Tukey’s post hoc test. (E) Secretion of the pro-inflammatory marker tissue inhibitor of metalloproteinase-1 (TIMP-1) measured by ELISA in whole lungs from neonatal rats exposed to normoxia (Nx Ct) or hyperoxia (Hx Ct), and treated or not with ^43^Gap26 (Nx Gap or Hx Gap). n = 5–6 animals per group. Data are presented as mean ± SEM. *P < 0.05, ***P < 0.001; one-way ANOVA followed by Tukey’s post hoc test. (F–G) Secretion of the pro-inflammatory cytokines interleukin-6 (IL-6) (F) and macrophage migration inhibitory factor (MIF) (G) measured by ELISA in supernatants of human fetal pulmonary artery smooth muscle cells (HfPA-SMC) exposed to room air (RA Ct) or hyperoxia (Hx Ct), and treated or not with ^43^Gap26 (RA Gap or Hx Gap). n = 8–10 measurements per group from 5–8 donors. Data are presented as mean ± SEM. *P < 0.05, ***P < 0.001, ****P < 0.0001; one-way ANOVA followed by Dunn’s multiple comparisons test.

We showed that hyperoxia significantly increased CD68 expression (marker of macrophages) in lungs as compared to the normoxic control group (p = 0.0448) (Fig 4A). However, Cx43 inhibition by ^43^Gap26 did not prevent this increase (Fig 4A).

In our animal model, we showed that hyperoxia did not modify M1 macrophage expression (CD86) (Fig 4B) but significantly increased M2 macrophage expression (CD206) as compared to the normoxic control group (p < 0.0001) (Fig 4C), leading to a significant decrease in M1 to M2 ratio (p = 0.0045) (Fig 4D). However, we demonstrated that Cx43 inhibition by ^43^Gap26 did not prevent this imbalance in M1/M2 polarization (Fig 4B, 4C and 4D).

Since hyperoxia increased macrophages infiltration, we then focused on cytokine production in the animal model and, after performing a cytokine array of different anti-inflammatory and pro-inflammatory cytokines (results not shown), we demonstrated that TIMP-1 expression was significantly increased by hyperoxia (p = 0.0009) but Cx43 inhibition by ^43^Gap26 did not prevent this increase (Fig 4E). In the same way, in HfPA-SMC, we demonstrated that hyperoxia significantly increased IL-6 (p = 0.0001) and MIF (p = 0.0402) expression (Fig 4F and 4G, respectively), and Cx43 inhibition by ^43^Gap26 did not prevent these increases.

### Effect of Cx43 inhibition by ^43^Gap26 on the extracellular matrix remodeling in the BPD animal model

In order to better understand why lung function was damaged while alveolar development was improved by ^43^Gap26, we studied whether ^43^Gap26 increased lung fibrosis.

**Fig 5:**
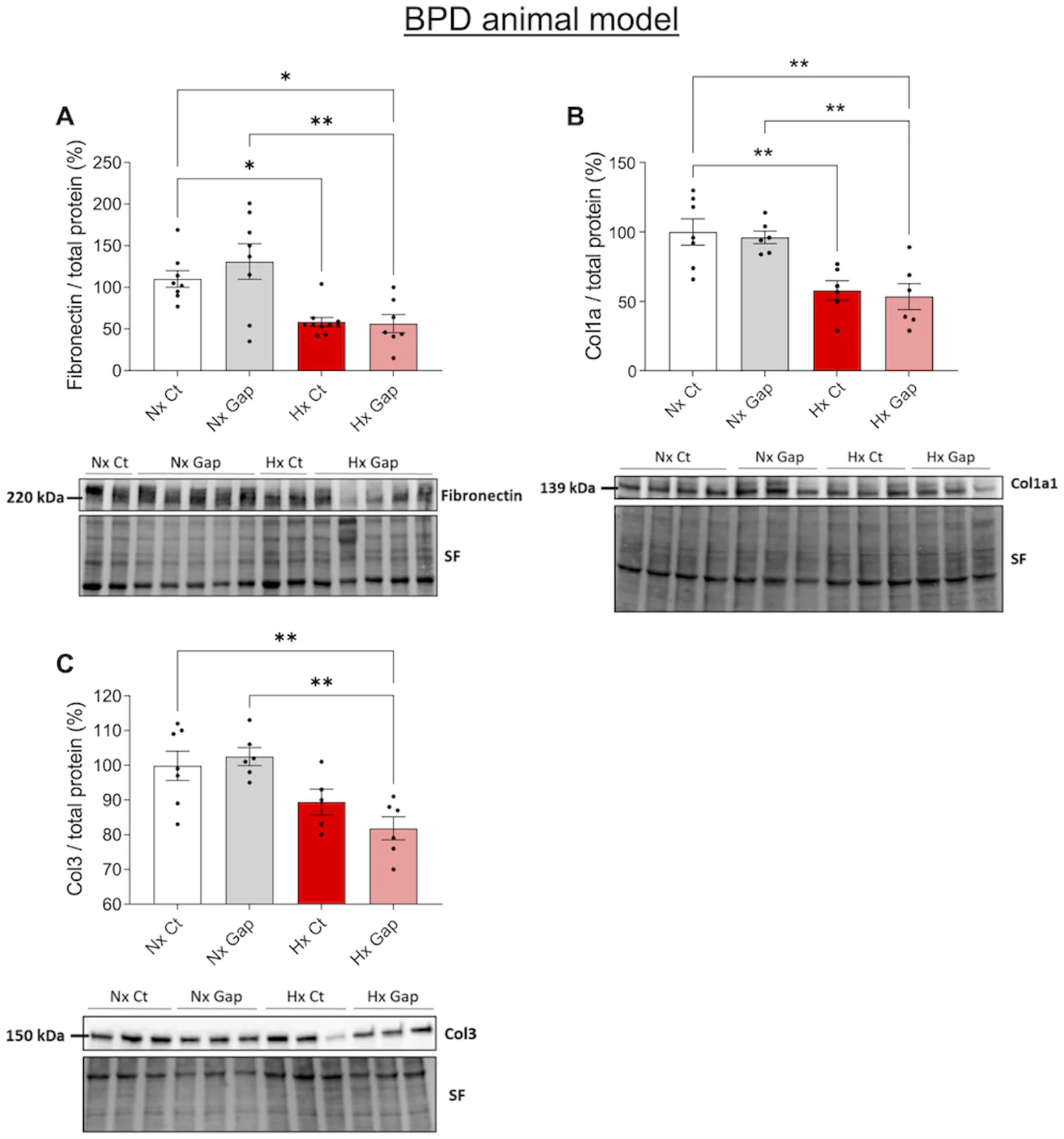
Effect of Cx43 inhibition on extracellular matrix remodeling in a rat model of bronchopulmonary dysplasia. (A) Expression of fibronectin analyzed by Western blot in whole lung homogenates from neonatal rats exposed to normoxia (Nx Ct) or hyperoxia (Hx Ct), and daily treated or not with the Cx43 inhibitor ^43^Gap26 (Nx Gap or Hx Gap). Representative immunoblots are shown (n = 7–10 per group). Protein levels were normalized to total protein using stain-free (SF) technology and expressed as a percentage of Nx Ct. Data are presented as mean ± SEM. *P < 0.05, **P < 0.01; one-way ANOVA followed by Tukey’s post hoc test. (B) Expression of collagen type I alpha (Col1a) assessed by Western blot in whole lungs from the same experimental groups as in (A). Representative immunoblots are shown (n = 6–7 per group). Protein levels were normalized to total protein (SF technology) and expressed as a percentage of Nx Ct. Data are presented as mean ± SEM. **P < 0.01; one-way ANOVA followed by Tukey’s post hoc test. (C) Expression of collagen type III (Col3) analyzed by Western blot in whole lungs from the same experimental groups as in (A). Representative immunoblots are shown (n = 6–7 per group). Protein levels were normalized to total protein (SF technology) and expressed as a percentage of Nx Ct. Data are presented as mean ± SEM. **P < 0.01; one-way ANOVA followed by Tukey’s post hoc test.

Thus, we showed that hyperoxia induced a decrease in fibronectin expression (p = 0.0277) and ^43^Gap26 treatment did not prevent this decrease (Fig 5A). Regarding collagen 1a and 3 expression, we demonstrated that hyperoxia significantly decreased collagen 1a expression (p = 0.0057) (Fig 5B) and tended to reduce collagen 3 expression (Fig 5C), and ^43^Gap26 did not prevent these decreases (Fig 5B and 5C).

### Effect of Cx43 inhibition by ^43^Gap26 on PH associated with BPD induced by hyperoxia in the BPD animal model

**Fig 6:**
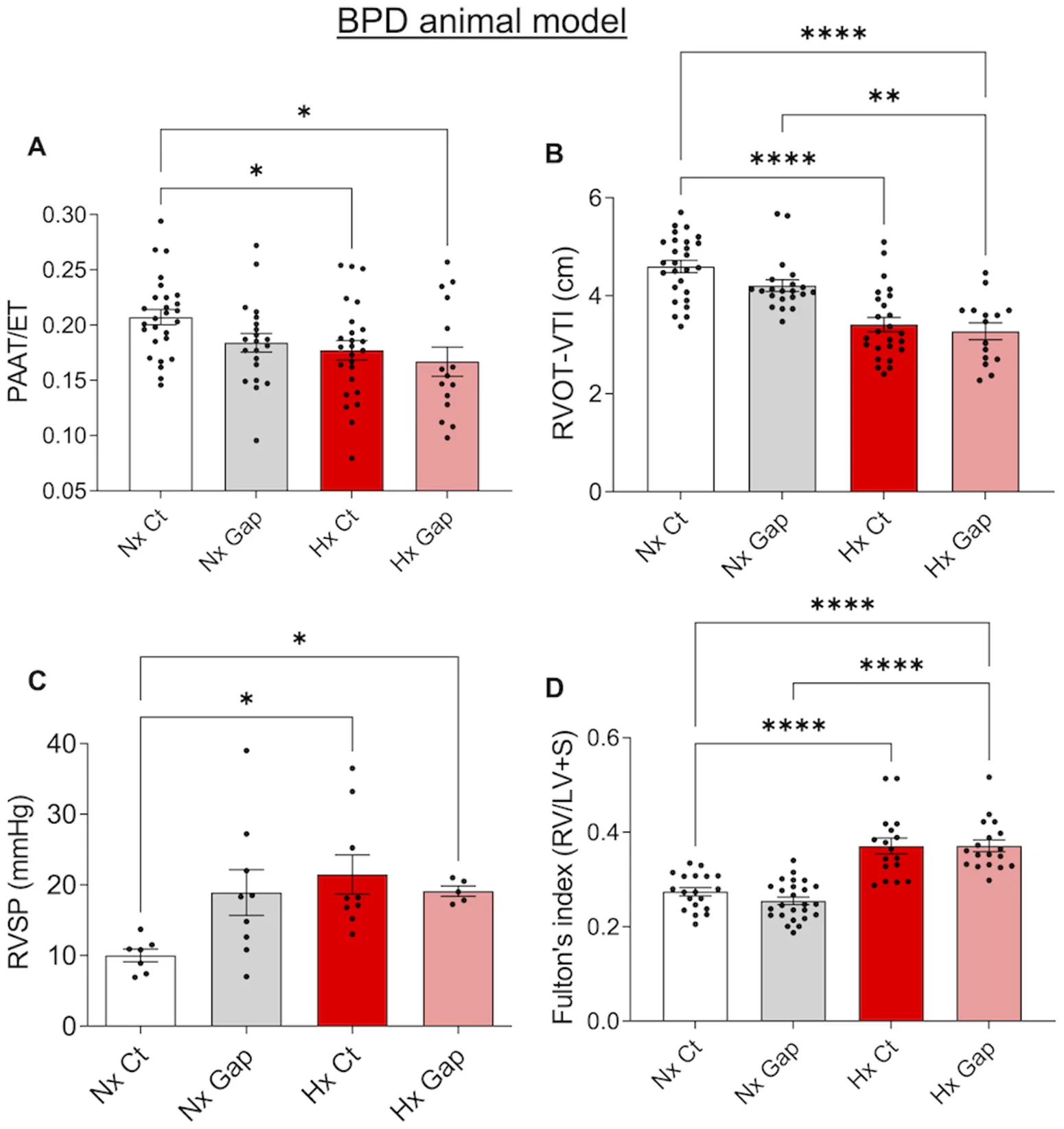
Effect of Cx43 inhibition on pulmonary hypertension associated with BPD (PH-BPD) in a neonatal rat model. (A) Ratio of pulmonary artery acceleration time (PAAT, ms) to ejection time (ET, ms) in neonatal rats exposed to normoxia (Nx Ct) or hyperoxia (Hx Ct) and daily treated or not with the Cx43 inhibitor ^43^Gap26 (Nx Gap or Hx Gap). n = 15–27 animals per group. Data are presented as mean ± SEM. *P < 0.05; one-way ANOVA followed by Sidak’s post hoc test. (B) Right ventricular outflow tract velocity time integral (RVOT-VTI, cm) in the same experimental groups as in (A). n = 15–27 animals per group. Data are presented as mean ± SEM. **P < 0.01, ****P < 0.0001; one-way ANOVA followed by Dunn’s post hoc test. (C) Right ventricular systolic pressure (RVSP, mmHg) measured in the same experimental groups as in (A). n = 5–9 animals per group. Data are presented as mean ± SEM. *P < 0.05; one-way ANOVA followed by Dunn’s post hoc test. (D) Right ventricular (RV) hypertrophy assessed by Fulton index (ratio of RV weight to left ventricle plus septum weight, LV + S) in the same experimental groups. n = 17–25 animals per group. Data are presented as mean ± SEM. ****P < 0.0001; one-way ANOVA followed by Tukey’s post hoc test.

As PH is the most severe complication of BPD, we then evaluated whether Cx43 inhibition by ^43^Gap26 may prevent PH-BPD in our animal model. Firstly, on echocardiographic measurements, ^43^Gap26 did not prevent the hyperoxia-induced decrease in PAAT/ET ratio (p = 0.0441) and RVOT-VTI (p < 0.0001) (Fig 6A and 6B, respectively). These results were confirmed in right heart catheterization, which showed a hyperoxia-induced increase in RVSP (p = 0.0144) without effect of ^43^Gap26 on this increase (Fig 6C). At last, in line with the previous results, Cx43 inhibition by ^43^Gap26 did not prevent hyperoxia-induced RV hypertrophy (p < 0.0001) as assessed by the Fulton index (Fig 6D). In conclusion, Cx43 inhibition by ^43^Gap26 did not prevent PH-BPD.

### Effect of Cx43 inhibition by ^43^Gap26 on hypoangiogenesis and PA remodeling induced by hyperoxia in the BPD animal model

To further understand the lack of effect of the ^43^Gap26 *in vivo* on pulmonary pressures, we studied vascular density and pulmonary arterial remodeling in our BPD animal model.

**Fig 7:**
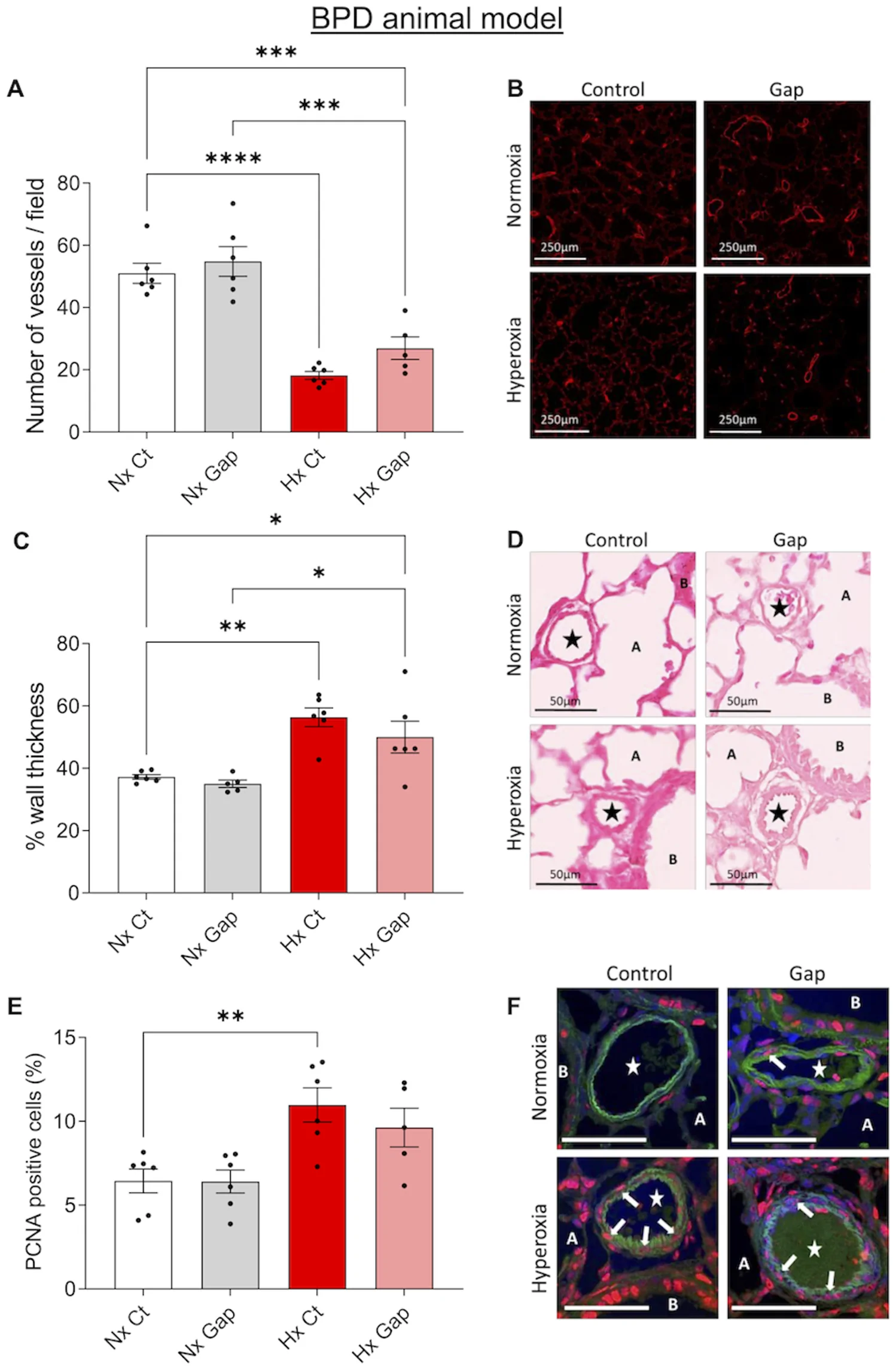
Effect of Cx43 inhibition on hyperoxia-induced hypoangiogenesis and pulmonary artery remodeling in a rat model of bronchopulmonary dysplasia. (A) Quantification of the number of vessels per field in lungs from neonatal rats exposed to normoxia (Nx Ct) or hyperoxia (Hx Ct) and daily treated or not with the Cx43 inhibitor ^43^Gap26 (Nx Gap or Hx Gap). n = 6 rats per group. Data are presented as mean ± SEM. ***P < 0.001, ****P < 0.0001; one-way ANOVA followed by Tukey’s post hoc test. (B) Representative von Willebrand Factor-immunostained (vWF) lung sections from the same experimental groups as in (A). Images shown are representative of n = 6 animals per group. Endothelium is labeled in red. (C) Percentage wall thickness of small pulmonary arteries (external diameter < 100 µm) in the same experimental groups as in (A). n = 5–6 animals per group. Data are presented as mean ± SEM. *P < 0.05, **P < 0.01; one-way ANOVA followed by Tukey’s post hoc test. (D) Representative hematoxylin and eosin-stained sections of small pulmonary arteries. Images shown are representative of n = 5–6 animals per group. Black stars indicate the PA lumen, A indicates alveoli, and B indicates bronchi lumen. (E) Percentage of PCNA-positive cells relative to total cells in the media of small pulmonary arteries in the same experimental groups as in (A). n = 5–6 animals per group. Data are presented as mean ± SEM. **P < 0.01; one-way ANOVA followed by Tukey’s post hoc test. (F) Representative Proliferating Cell Nuclear Antigen-immunostained (PCNA) small pulmonary artery sections. Images shown are representative of n = 5–6 animals per group. Proliferating cell nuclei are labeled in red, all nuclei with DAPI (blue), and elastic lamina autofluorescence is shown in green. A indicates alveoli, B indicates bronchi lumen, white stars indicate PA lumen, and white arrows highlight PCNA-positive cells.

First, we demonstrated that Cx43 inhibition by ^43^Gap26 did not prevent hypoangiogenesis induced by hyperoxia (p < 0.0001) (Fig 7A and 7B). Moreover, ^43^Gap26 treatment did not prevent hyperoxia-induced increase in PA wall thickness (p = 0.0018) (Fig 7C and 7D). This result was consistent with the fact that ^43^Gap26 had no effect on the significant increase in PA-SMC proliferation induced by hyperoxia (p = 0.0084) (Fig 7E and 7F).

### Effect of Cx43 inhibition by ^43^Gap26 on PA hyperreactivity induced by hyperoxia in the BPD animal model

Since alterations in vascular reactivity, including sustained pulmonary vasoconstriction, are involved in PH development [15], we evaluated PA reactivity to vasocontracting agents by myography. PA from neonatal rats exposed to hyperoxia displayed a significant hyperreactivity to serotonin (p = 0.0382), phenylephrine (p < 0.0001) and ET-1 (p = 0.0350) as compared to the normoxic control group, and Cx43 inhibition by ^43^Gap26 did not prevent this hyperreactivity (S2A, S2B and S2C Fig, respectively).

### Effect of Cx43 inhibition by ^43^Gap26 on the oxidative stress in both animal and human models

BPD and PH-BPD have multifactorial origins but one of their main risk factor is long-term exposure to hyperoxia and consequently the reactive oxygen species (ROS) produced [16]. Therefore, we first studied lipid peroxidation and protein carbonylation due to oxidative stress in the BPD animal model and we showed no significant difference in hyperoxic conditions compared to controls, with or without ^43^Gap26 (S3A and S3B Fig, respectively). We then studied the anti-oxidant response and demonstrated that hyperoxia significantly increased HO-1 expression in whole lungs and in intrapulmonary arteries (p = 0.0019 and < 0.0001, respectively) (S3C and S3D Fig). Moreover, we highlighted that Cx43 inhibition by ^43^Gap26 did not alter the increase in HO-1 expression in whole lungs (p = 0.0090) (S3C Fig) whereas in the intrapulmonary arteries, ^43^Gap26 significantly decreased HO-1 expression as compared to the hyperoxic control group (p = 0.0163) (S3D Fig).

In HfPA-SMC, unlike in rat intrapulmonary arteries, hyperoxia also significantly increased HO-1 expression (p = 0.0299) but ^43^Gap26 did not alter this increase (S3E Fig).

### Effect of Cx43 inhibition by ^43^Gap26 on the increased mortality in the BPD animal model

PH-BPD is a severe disease responsible for high and early mortality [6,17,18]. Therefore, we analyzed survival in our BPD animal model and we confirmed that hyperoxia significantly increased mortality as compared to the normoxic control group (p = 0.0064) (S4A Fig), and Cx43 inhibition by ^43^Gap26 did not prevent this hyperoxia-induced mortality (S4A Fig).

Finally, as growth retardation is a key factor in the severity and mortality of BPD and PH-BPD [2,19], we monitored the animals’ growth during the protocol and found no difference in weight between the two conditions (S4B Fig).

## Discussion

In this study, using a well-described neonatal rat model of hyperoxia-induced BPD, we found that hyperoxia increased Cx43 expression and intrapulmonary inflammation characterized by an increase in (i) recruitment of intrapulmonary macrophages with an M1/M2 polarization imbalance in favor of M2, and in (ii) synthesis of pro-inflammatory cytokines, concomitantly with a blunted alveolar development, an arrested pulmonary vascularization, the onset of PH-BPD and an increased mortality rate. To explore the link between Cx43 expression and intrapulmonary inflammation in BPD, we treated neonatal rats exposed to hyperoxia with a selective Cx43 inhibitor, ^43^Gap26. Our results indicated that this treatment (i) reversed hyperoxia-induced increase in Cx43 lung expression in neonatal rats, (ii) did not prevent hyperoxia-induced BPD features (namely altered expression of SP-B, disruption of extracellular matrix structures, increased intrapulmonary macrophages infiltration and pro-inflammatory cytokine secretion) despite an improvement in alveolar development, (iii) did not prevent the onset of PH-BPD, and (iv) even further disturbed some parameters of BPD (lung elastance and HO-1 expression). These results consequently show that Cx43 appears to be involved in hyperoxia-induced abnormalities in alveolar development associated with BPD. Therefore, although selective inhibition of Cx43 has limited effects in treating BPD, its beneficial effect on alveolarization requires further studies to better understand the underpinning mechanisms.

### ATII cell alterations

Studies using lineage tracing experiments demonstrate that hyperoxia causes changes in senescence and proliferation in ATII cells [20,21]. Senescence has been recently considered as an important driving mechanism for chronic lung diseases, including BPD. Hyperoxia exposure of ATII cells from mice at the saccular stage or human fetal airway smooth muscle cells induced an increase in the senescence-associated secretory phenotype (SASP) (namely secretion of pro-inflammatory cytokines such as TIMP1 and IL-6) contributing to inflammation and tissue remodeling *via* paracrine effect on non-senescent adjacent cells [22,23]. In mice, such SASP was involved in both alveolar and vascular rarefaction [22]. Moreover, physiological senescence induced by ageing in other models such as cardiomyocytes or cerebral vascular cells, was associated with a Cx43 decreased expression [24,25]. These observations and ours raise the possibility that Cx43 signaling could be linked to cellular senescence pathways in BPD. However, in the present study, we did not directly quantify ATII cell number or assess specific senescence markers. Therefore, our findings based on pro-SPC and TIMP-1 expression should be interpreted cautiously and do not allow definitive conclusions regarding ATII senescence.

Although ^43^Gap26 reduced Cx43 expression and improved alveolarization under hyperoxic conditions, our data do not establish a direct mechanistic link between Cx43 modulation and ATII senescence. The observed changes may reflect alterations in ATII cell status or function, but additional targeted investigations would be required to clarify this relationship. Interestingly, improved alveolarization following Cx43 inhibition has also been reported by Qing *et al*. [14]. However, in contrast to that study, we assessed lung mechanics and PH-BPD in parallel and observed that this beneficial effect of ^43^Gap26 was accompanied by an alteration in lung function (compliance, tissue damping and elastance), with similar rates of PH-BPD and mortality observed in either ^43^Gap26-treated or untreated animals. Moreover, Qing *et al*. linked the improved alveolarization associated to ^43^Gap26 to a reduction of both oxidative stress and excessive alveolar apoptosis. However, in our study, we were not able to observe any oxidative stress, but differences in species or experimental conditions may partly explain these discrepancies.

Overall, our findings suggest that Cx43 modulation is associated with improved alveolar structure under hyperoxia, but further studies are needed to determine the precise cellular and molecular mechanisms involved.

### The pulmonary surfactant

Consistently with our results, several studies on different animal models have shown that hyperoxia is involved in impaired surfactant synthesis and function [26,27]. Lamellar bodies exocytosis by ATII cells is triggered by an increase in cytosolic Ca^2+^ concentration in ATII cells, resulting mainly from communication with ATI cells, either through GJ or through paracrine pathways [28]. Since Cx43 inhibition with ^43^Gap26 completely and reversibly blocked such process, we can presume that ATI cells act as sensors that transmit Ca^2+^ transients to ATII cells via Cx43-GJ to induce lamellar bodies’ exocytosis and thus pulmonary surfactant production [29]. However, GJ-independent release of surfactant also occurs through ATP secretion and paracrine stimulation of purinergic receptors [30]. It may thus explain why ^43^Gap26 does not decrease the SP-B expression in Nx Ct conditions nor exacerbates SP-B decrease induced by hyperoxia in our study.

### Macrophages-induced inflammation

Connexins are known to be involved in inflammation and Cx43 is the most expressed connexin in macrophages [31]. BPD and PH-BPD are characterized by an increased secretion of pro-inflammatory cytokines and an imbalance between the inflammatory cells that cause tissue damage and those that promote tissue repair in lungs [32,33]. Anti-inflammatory approaches attenuate hyperoxia-induced hypoalveolarization in a neonatal mice model of BPD [5,34] and pulmonary vascular remodeling in adult rodent models of pulmonary arterial hypertension (PAH) [35,36]. Alveolar macrophages (AMs) exhibit functional heterogeneity and plasticity depending on the microenvironment and they can be divided into 2 populations: M1 macrophages, which release pro-inflammatory cytokines and M2 macrophages, which express anti-inflammatory and wound repair mediators. However, any imbalance in macrophage M1/M2 polarization may have adverse effects in wound healing and tissue repair leading to various diseases or inflammatory conditions [4,37]. In our study, we showed an increased expression of CD68 (AM marker) and CD206 (M2 marker), highlighting increased intrapulmonary recruitment of macrophages and their M2 polarization. Interestingly, a recent study found that AMs from newborn mice exposed to hyperoxia acquired a senescence-like phenotype leading to a change in their polarization status towards the M2 phenotype [22]. In addition, in adult mice and humans with PH or PAH, M2 macrophages have been shown to be increased and involved in PA remodeling [35,36]. These results, together with ours, suggest that M2 macrophages play a critical role in modulating the pulmonary inflammatory response in BPD and PH-BPD. Among AMs, a cluster of cells is connected to the alveolar epithelium via Cx43-GJ and thus communicates with each other through synchronized Ca^2+^ signals, using the epithelium as a conducting pathway [38]. In this study, the authors showed that the selective inhibition of Cx43-GJ blocked the synchronous Ca^2+^ signals between AMs and the epithelium resulting in an increased inflammation. These results were confirmed in a mouse model with a specific deletion of Cx43 in AMs (CD11cCx43^-/-^) where lung inflammation was markedly greater compared to wild type mice. In our model, ^43^Gap26 did not worsen TIMP-1 secretion in animals exposed to hyperoxia (Hx Gap). Since ATII cell numbers were significantly reduced in the Hx Gap group, and since ATII cells have been shown to participate in pro-inflammatory cytokine secretion together with AMs [37,38], we can assume that it could be due to an increase in TIMP-1 synthesis by AMs instead of ATII cells.

### Inflammation

Inflammation is an early and persistent feature of PH characterized by inflammatory cells infiltration such as macrophages and increased secretion of various inflammatory markers (including IL-6, TIMP-1 and MIF) which are correlated with PH severity. These inflammatory markers are secreted by various cell types in lung, including ATII cells, PA-SMC and infiltrated macrophages [39,40]. IL-6 is a circulating pro-inflammatory cytokine that contributes to cell proliferation, migration and differentiation in lungs [41]. In premature newborns, patients with BPD and PH-BPD exhibit increased IL-6 levels in both serum and tracheal aspiration which correlate with their prognoses [42]. All these results agree with ours showing an increase in IL-6 in HfPA-SMC exposed to hyperoxia. In patients with BPD, TIMP-1 expression, a key mediator of acute inflammation and wound repair process mainly secreted by macrophages and ATII cells in the lung, is increased [43]. In addition, our group has previously shown that Cx43 expression is increased in PA from CH-PH patients and that knockdown mice for Cx43 (Cx43^+/-^) were partially protected against CH-PH with reduced PA remodeling and inflammation [15]. Indeed, in the lung capillary bed, Cx43-GJ is thought to mediate an interendothelial Ca^2+^ signal involved in the activation of pro-inflammatory pathways [44]. In our study, on one hand, we found no evidence of altered Cx43 expression in PA from the *in vivo* model and treatment with ^43^Gap26 had no effect on PH-BPD (namely high RVSP, RV hypertrophy, PA remodeling and hyperreactivity) and on the other hand, in HfPA-SMC and whole lungs, we demonstrated a hyperoxia-induced increase in Cx43 expression and pro-inflammatory secretion. However, ^43^Gap26 had no effect on inflammation suggesting that the role of Cx43 in inflammation may be different in adult *versus* neonates and/or there are species differences (mice *versus* rat). Altogether, although ^43^Gap26 has no deleterious effect on PH-BPD, it has no beneficial effect either.

### The extracellular matrix

The ECM serves as a framework to guide lung development, and ECM composition continuously evolve as the lung matures. Collagen is the most abundant protein of the lung ECM (50% of ECM components), predominantly represented by fibrillary collagens (collagen 1 and 3) produced by fibroblasts. Collagens are found in the bronchi, blood vessels and alveolar septa, and increase throughout lung development. Fibronectin is a major blood and tissue glycoprotein, and a major component of the ECM. Several studies have documented the increased expression of collagen and fibronectin in preterm newborns with BPD in endotracheal aspirates, BAL or lung samples [45,46]. These results run counter to those of our study. However, the vast majority of these studies were carried out using samples collected during the period of the “old BPD”, characterized by significant pulmonary fibrosis due to an aggressive mechanical ventilation, a maximal O_2_ supplementation and the absence of exogenous surfactant supply, making it difficult to compare with our results. Regarding animal studies, although there is a tendency for collagen and fibronectin to increase in hyperoxia-induced BPD models, it remains controversial depending on species, oxygen concentration, duration of exposure, developmental stage at injury, and time point of analysis. These methodological differences likely contribute to the variability across studies and may account for the apparent discrepancies with our results. However, as in our results, a recent study found that elevated IL-33/ST2 (receptor suppression of tumorigenicity 2) signaling pathway in a double-hit neonatal mice model of BPD induced by hyperoxia and intraperitoneal injection of LPS led to a decrease in fibronectin level in lungs [47]. The authors showed that IL-33 promoted the formation of neutrophil extracellular traps (NETs) carrying proteases, which degraded fibronectin in alveolar epithelial cells causing BPD-like disease. These results are in line with other studies on the hyperoxia-induced neonatal rat model of BPD, showing that inhibition of NETs by heparin or vitamin D improved survival, alveolarization and vascular development, and decreased inflammation [48,49]. These findings suggest that extracellular matrix remodeling in hyperoxia-induced lung injury may be context-dependent and not uniformly characterized by increased collagen or fibronectin deposition.

### Limitations

There are several limitations to this study, firstly those inherent in the experimental models used. Although continuous high-concentration hyperoxia does not fully reproduce the heterogeneous oxygen exposure and clinical complexity of contemporary human BPD, it remains one of the most widely used and well-characterized experimental models of neonatal lung injury. Severe hyperoxia exposure (≥85–95% O_2_) has consistently been shown to induce impaired alveolarization, vascular remodeling, and inflammatory responses, and has been extensively employed to investigate the molecular and cellular mechanisms underlying BPD pathogenesis [2,50]. The reproducibility and robustness of this model provide a strong framework for mechanistic studies and allow direct comparison with a substantial body of literature. Nevertheless, alternative models using moderate (e.g., ~ 60% O_2_) or intermittent hyperoxia protocols may better reproduce certain aspects of patchy alveolar hypoplasia and pulmonary hypertension observed in current clinical settings. However, each model captures distinct pathological features, and the severe hyperoxia model remains particularly useful for investigating defined molecular pathways under controlled and reproducible injury conditions. Future studies using complementary models may further refine the translational implications of Cx43 modulation in BPD complicated by pulmonary hypertension.

An additional limitation of the rat model is its relatively mature antioxidant capacity at birth compared with extremely premature infants [51]. This increased tolerance to hyperoxia may partly explain why classical markers of oxidative damage (protein carbonylation and lipid peroxidation) were not significantly elevated under our experimental conditions, despite sustained oxygen exposure. However, the hyperoxia-induced neonatal rat model of BPD is highly reproducible and quantifiable and has several similarities with BPD, enabling to further improve knowledge on lung development, injury and repair that will ultimately benefit premature newborns.

The use of whole-lung homogenates provides a global overview of molecular alterations but lacks spatial and cell-type–specific resolution. Consequently, our findings should be interpreted as reflecting overall pathway involvement rather than precise cellular mechanisms. Future studies employing immunohistochemistry, lineage tracing, or cell-specific approaches will be required to further dissect the role of Cx43 in distinct pulmonary compartments, especially the alveoli.

Regarding the *in vitro* experiments, HfPA-SMC, unlike the *in vivo* model, only focuses on PA without consideration on bronchial and alveolar tissues. Therefore, since (i) lung samples from patients who have died of BPD are rare, (ii) our *in vitro* human model does not fully recapitulate the complexities of the *in vivo* microenvironment and (iii) no integrated viable *in vitro* models exist on human fetal PA, HfPA-SMC were used in order to confirm and further demonstrate some mechanistic underpinnings of Cx43 on inflammation and oxidative stress.

Finally, although ^43^Gap26 has already been used in various disease models *in vivo*, questions remain about its pharmacokinetics, stability, optimal route of administration, and potential systemic effects, given the ubiquitous expression of Cx43. Further pharmacological and safety studies will be required before considering translational applications.

## Conclusions

Our novel results highlighted that, despite an increase in Cx43 expression induced by hyperoxia in an animal model of BPD, Cx43 seems only partially involved in BPD pathophysiology. Indeed, selective inhibition of Cx43 by ^43^Gap26 did not prevent most of the hallmarks of BPD, *i.e.,* arrested vascular development, intrapulmonary inflammation, decreased surfactant production nor the occurrence of PH-BPD, nor the poor survival. Together, these results suggest that while Cx43-GJ modulation influences alveolar development under hyperoxic conditions, targeting this pathway alone does not provide comprehensive protection against the multifactorial pathology of experimental BPD.

## Supporting information

S1 Fig Neonatal rat BPD model and human fetal pulmonary artery smooth muscle cells (HfPA-SMC) culture. (A) Neonatal Wistar rats were exposed to normoxia (Nx, 21 % O_2_) or hyperoxia (Hx, 90 % O_2_) from postnatal day 1 (D1) to day 14 (D14). Animals were daily intraperitoneally injected with ^43^Gap26 (50 µg/kg/day; 10 µL/g body weight) or an equal volume of saline solution (Ct). n = 12 pups per group. i.p. indicates intraperitoneally. (B) HfPA-SMC were obtained from intrapulmonary arteries dissected from human fetal lungs after elective termination of pregnancy (12–22 weeks of amenorrhea). Cells were treated or not with ^43^Gap26 (300 µM) and exposed *in vitro* to room air (RA, 21 % O_2_, 5 % CO_2_) or hyperoxia (Hx, 60 % O_2_, 5 % CO_2_) for 48 h in a humidified incubator at 37°C. DMEM indicates Dulbecco’s Modified Eagle Medium. Illustrations were created with BioRender.com.
(TIFF)

S2 Fig Effect of Cx43 inhibition on hyperoxia-induced pulmonary artery hyperreactivity in a neonatal rat model of BPD. (A) Contraction of neonatal rat intrapulmonary arterial rings induced by cumulative concentrations of serotonin (5-HT, 10^−9^ to 10^−3^ mol/L). Rings were collected at postnatal day 14 from rats exposed to normoxia (Nx Ct) or hyperoxia (Hx Ct) and daily treated with the Cx43 inhibitor ^43^Gap26 (Nx Gap or Hx Gap). Data represent means ± SEM. n = 4-12 arterial rings per group from 2-5 animals per condition. *P < 0.05. Two-way ANOVA followed by post-hoc Sidak’s multiple comparisons test. (B) Contraction of rat intrapulmonary arterial rings induced by cumulative concentrations of phenylephrine (Phe, 10^−10^ to 10^−4^ mol/L). Data were collected as in (A). Data represent means ± SEM. n = 4-12 arterial rings per group from 2-5 animals per condition. *P < 0.05 and ****P < 0.0001. Two-way ANOVA followed by post-hoc Sidak’s multiple comparisons test. (C) Contraction of rat intrapulmonary arterial rings induced by cumulative concentrations of endothelin-1 (ET-1, 10^−10^ to 10^−7^ mol/L). Data were collected as in (A). Data represent means ± SEM. n = 4-12 arterial rings per group from 2-5 animals per condition. *P < 0.05 and **P < 0.01. Two-way ANOVA followed by post-hoc Sidak’s multiple comparisons test.
(TIFF)

S3 Fig Effects of hyperoxia and Cx43 inhibition on the oxidative stress and the antioxidant enzyme HO-1 in both models. (A) Expression of 4-hydroxynonenal (4-HNE – lipid peroxidation), analyzed by Western blot in whole lung homogenates from neonatal rats exposed to normoxia (Nx Ct) or to hyperoxia (Hx Ct) and daily treated with the Cx43 inhibitor ^43^Gap26 (Nx Gap or Hx Gap). Representative immunoblots are shown (n = 6-7 animals per group). Protein levels were normalized to total protein using stain-free (SF) technology and expressed as a percentage of Nx Ct. Data represent means ± SEM; one-way ANOVA followed by Tukey’s post-hoc test. (B) Protein oxidation level assessed by anti-2,4-Dinitrophenol (DNP) immunoassay (Oxyblot) in the same experimental groups as in (A). Representative immunoblots are shown (n = 4-7 animals per group). Protein levels were normalized to total protein (SF technology) and expressed as a percentage of Nx Ct. Data represent means ± SEM; one-way ANOVA followed by Tukey’s post-hoc test. (C-D) Expression of heme oxygenase-1 (HO-1) assessed by Western blot anlysis in whole lungs (C) and intrapulmonary arteries (D) from the same experimental groups as in (A). Representative immunoblots are shown (n = 5-8 animals per group). Protein levels were normalized to total protein (SF technology) and expressed as a percentage of Nx Ct. Data represent means ± SEM; one-way ANOVA followed by Dunn’s (C) and Tukey’s (D) post-hoc test. (E) Expression of HO-1 assessed by Western blot in human fetal pulmonary artery smooth muscle cells (HfPA-SMC) exposed to room air (RA Ct) or hyperoxia (Hx Ct) and treated with ^43^Gap26 (RA Gap or Hx Gap). Representative immunoblots are shown (n = 5 donors per group). Protein levels were normalized to total protein (SF technology) and expressed as a percentage of RA Ct. Data represent means +/- SEM. *P < 0.05, **P < 0.01 and ****P < 0.0001; one-way ANOVA followed by Tukey’s post-hoc test.
(TIFF)

S4 Fig Effect of Cx43 inhibition on hyperoxia-induced mortality and body weight in a neonatal rat model of BPD. (A) Kaplan-Meyer survival curve for the first 14 postnatal days in neonatal rats exposed to normoxia (Nx Ct) or to hyperoxia (Hx Ct) and daily treated with the Cx43 inhibitor ^43^Gap26 (Nx Gap or Hx Gap). The survival curve for condition Nx Gap (purple line) overlaps with the Nx Ct (blue line) and is therefore not visible. n = 15-32 animals per group. **P < 0.01; log-rank test. (B) Mean body weight over the first 14 postnatal days in the same animal groups as in (A). n = 18-25 animals per group. Data represent means +/- SEM; one-way ANOVA followed by Tukey’s post-hoc test.
(TIFF)

S1 Table Primary antibodies used for Western blotting and immunostaining experiments. (DOCX)

S1 File Membranes. (PDF)

S2 File Raw data. (XLSX)

## References

[1] Northway WH, Rosan RC, Porter DY. Pulmonary disease following respirator therapy of hyaline-membrane disease. Bronchopulmonary dysplasia. N Engl J Med. 1967;276(7):357–68. doi: 10.1056/NEJM196702162760701 5334613

[2] Thébaud B, Goss KN, Laughon M, Whitsett JA, Abman SH, Steinhorn RH, et al. Bronchopulmonary dysplasia. Nat Rev Dis Primers. 2019;5(1):78. doi: 10.1038/s41572-019-0127-7 31727986 PMC6986462

[3] Kalymbetova TV, Selvakumar B, Rodríguez-Castillo JA, Gunjak M, Malainou C, Heindl MR, et al. Resident alveolar macrophages are master regulators of arrested alveolarization in experimental bronchopulmonary dysplasia. J Pathol. 2018;245(2):153–9. doi: 10.1002/path.5076 29574785

[4] Arora S, Dev K, Agarwal B, Das P, Syed MA. Macrophages: their role, activation and polarization in pulmonary diseases. Immunobiology. 2018;223(4–5):383–96. doi: 10.1016/j.imbio.2017.11.001 29146235 PMC7114886

[5] Hirani D, Alvira CM, Danopoulos S, Milla C, Donato M, Tian L, et al. Macrophage-derived IL-6 trans-signalling as a novel target in the pathogenesis of bronchopulmonary dysplasia. Eur Respir J. 2022;59(2):2002248. doi: 10.1183/13993003.02248-2020 34446466 PMC8850688

[6] Mourani PM, Sontag MK, Younoszai A, Miller JI, Kinsella JP, Baker CD, et al. Early pulmonary vascular disease in preterm infants at risk for bronchopulmonary dysplasia. Am J Respir Crit Care Med. 2015;191(1):87–95. doi: 10.1164/rccm.201409-1594OC 25389562 PMC4299632

[7] Hansmann G, Sallmon H, Roehr CC, Kourembanas S, Austin ED, Koestenberger M, et al. Pulmonary hypertension in bronchopulmonary dysplasia. Pediatr Res. 2021;89(3):446–55. doi: 10.1038/s41390-020-0993-4 32521539 PMC7979539

[8] Al-Ghanem G, Shah P, Thomas S, Banfield L, El Helou S, Fusch C, et al. Bronchopulmonary dysplasia and pulmonary hypertension: a meta-analysis. J Perinatol. 2017;37(4):414–9. doi: 10.1038/jp.2016.250 28079864

[9] Altit G, Bhombal S, Hopper RK, Tacy TA, Feinstein J. Death or resolution: the “natural history” of pulmonary hypertension in bronchopulmonary dysplasia. J Perinatol. 2019;39(3):415–25. doi: 10.1038/s41372-018-0303-8 30617286

[10] Freund-Michel V, Muller B, Marthan R, Savineau J-P, Guibert C. Expression and role of connexin-based gap junctions in pulmonary inflammatory diseases. Pharmacol Ther. 2016;164:105–19. doi: 10.1016/j.pharmthera.2016.04.004 27126473

[11] Nagata K, Masumoto K, Esumi G, Teshiba R, Yoshizaki K, Fukumoto S, et al. Connexin43 plays an important role in lung development. J Pediatr Surg. 2009;44(12):2296–301. doi: 10.1016/j.jpedsurg.2009.07.070 20006013

[12] Sarieddine MZR, Scheckenbach KEL, Foglia B, Maass K, Garcia I, Kwak BR, et al. Connexin43 modulates neutrophil recruitment to the lung. J Cell Mol Med. 2009;13(11–12):4560–70. doi: 10.1111/j.1582-4934.2008.00654.x 19166484 PMC4515071

[13] Huang J-Q, Chen X-Y, Huang F, Fan J-M, Shi X-W, Ju Y-K. Effects of Connexin 43 inhibition in an ovalbumin-induced mouse model of asthma. Iran J Allergy Asthma Immunol. 2018;17(1):29–38. 29512367

[14] Qing C, Xinyi Z, Xuefei Y, Xindong X, Jianhua F. The specific Connexin 43-Inhibiting Peptide Gap26 improved alveolar development of neonatal rats with hyperoxia exposure. Front Pharmacol. 2021;12:587267. doi: 10.3389/fphar.2021.587267 34290603 PMC8287833

[15] Bouvard C, Genet N, Phan C, Rode B, Thuillet R, Tu L, et al. Connexin-43 is a promising target for pulmonary hypertension due to hypoxaemic lung disease. Eur Respir J. 2020;55(3):1900169. doi: 10.1183/13993003.00169-2019 31862763

[16] Wang J, Dong W. Oxidative stress and bronchopulmonary dysplasia. Gene. 2018;678:177–83. doi: 10.1016/j.gene.2018.08.031 30098433

[17] Khemani E, McElhinney DB, Rhein L, Andrade O, Lacro RV, Thomas KC, et al. Pulmonary artery hypertension in formerly premature infants with bronchopulmonary dysplasia: clinical features and outcomes in the surfactant era. Pediatrics. 2007;120(6):1260–9. doi: 10.1542/peds.2007-0971 18055675

[18] Altit G, Bhombal S, Feinstein J, Hopper RK, Tacy TA. Diminished right ventricular function at diagnosis of pulmonary hypertension is associated with mortality in bronchopulmonary dysplasia. Pulm Circ. 2019;9(3). doi: 10.1177/2045894019878598 31662848 PMC6792284

[19] Nagiub M, Kanaan U, Simon D, Guglani L. Risk factors for development of pulmonary hypertension in infants with bronchopulmonary dysplasia: systematic review and meta-analysis. Paediatr Respir Rev. 2017;23:27–32. doi: 10.1016/j.prrv.2016.11.003 28188008

[20] Scaffa A, Yao H, Oulhen N, Wallace J, Peterson AL, Rizal S, et al. Single-cell transcriptomics reveals lasting changes in the lung cellular landscape into adulthood after neonatal hyperoxic exposure. Redox Biol. 2021;48:102091. doi: 10.1016/j.redox.2021.102091 34417156 PMC8710996

[21] Dennery PA, Yao H. Emerging role of cellular senescence in normal lung development and perinatal lung injury. Chin Med J Pulm Crit Care Med. 2024;2(1):10–6. doi: 10.1016/j.pccm.2024.01.001 38567372 PMC10987039

[22] Yao H, Wallace J, Peterson AL, Scaffa A, Rizal S, Hegarty K, et al. Timing and cell specificity of senescence drives postnatal lung development and injury. Nat Commun. 2023;14(1):273. doi: 10.1038/s41467-023-35985-4 36650158 PMC9845377

[23] Parikh P, Britt RD Jr, Manlove LJ, Wicher SA, Roesler A, Ravix J, et al. Hyperoxia-induced cellular senescence in fetal airway smooth muscle cells. Am J Respir Cell Mol Biol. 2019;61(1):51–60. doi: 10.1165/rcmb.2018-0176OC 30508396 PMC6604224

[24] Zhan R, Meng X, Tian D, Xu J, Cui H, Yang J, et al. NAD+ rescues aging-induced blood-brain barrier damage via the CX43-PARP1 axis. Neuron. 2023;111(22):3634–3649.e7. doi: 10.1016/j.neuron.2023.08.010 37683629

[25] Nagibin V, Egan Benova T, Viczenczova C, Szeiffova Bacova B, Dovinova I, Barancik M, et al. Ageing related down-regulation of myocardial connexin-43 and up-regulation of MMP-2 may predict propensity to atrial fibrillation in experimental animals. Physiol Res. 2016;65 Suppl 1:S91–100. doi: 10.33549/physiolres.933389 27643943

[26] Zenri H, Rodriquez-Capote K, McCaig L, Yao L-J, Brackenbury A, Possmayer F, et al. Hyperoxia exposure impairs surfactant function and metabolism. Crit Care Med. 2004;32(5):1155–60. doi: 10.1097/01.ccm.0000126264.00551.c8 15190966

[27] Jin Y, Peng L-Q, Zhao A-L. Hyperoxia induces the apoptosis of alveolar epithelial cells and changes of pulmonary surfactant proteins. Eur Rev Med Pharmacol Sci. 2018;22(2):492–7. doi: 10.26355/eurrev_201801_14200 29424908

[28] Johnson LN, Koval M. Cross-talk between pulmonary injury, oxidant stress, and gap junctional communication. Antioxid Redox Signal. 2009;11(2):355–67. doi: 10.1089/ars.2008.2183 18816185 PMC2933150

[29] Ichimura H, Parthasarathi K, Lindert J, Bhattacharya J. Lung surfactant secretion by interalveolar Ca2+ signaling. Am J Physiol Lung Cell Mol Physiol. 2006;291(4):L596–601. doi: 10.1152/ajplung.00036.2006 16698857

[30] Patel AS, Reigada D, Mitchell CH, Bates SR, Margulies SS, Koval M. Paracrine stimulation of surfactant secretion by extracellular ATP in response to mechanical deformation. Am J Physiol Lung Cell Mol Physiol. 2005;289(3):L489–96. doi: 10.1152/ajplung.00074.2005 15908478

[31] He P, Dai M, Li Z, Wang X, Liu H, He Y, et al. Effect of connexin 43 in LPS/IL-4-induced macrophage M1/M2 polarization: An observational study. Medicine (Baltimore). 2024;103(15):e37811. doi: 10.1097/MD.0000000000037811 38608055 PMC11018209

[32] Zhang Z-Q, Huang X-M, Lu H. Early biomarkers as predictors for bronchopulmonary dysplasia in preterm infants: a systematic review. Eur J Pediatr. 2014;173(1):15–23. doi: 10.1007/s00431-013-2148-7 23996017

[33] Giusto K, Wanczyk H, Jensen T, Finck C. Hyperoxia-induced bronchopulmonary dysplasia: better models for better therapies. Dis Model Mech. 2021;14(2):dmm047753. doi: 10.1242/dmm.047753 33729989 PMC7927658

[34] Nold MF, Mangan NE, Rudloff I, Cho SX, Shariatian N, Samarasinghe TD, et al. Interleukin-1 receptor antagonist prevents murine bronchopulmonary dysplasia induced by perinatal inflammation and hyperoxia. Proc Natl Acad Sci U S A. 2013;110(35):14384–9. doi: 10.1073/pnas.1306859110 23946428 PMC3761642

[35] Hashimoto-Kataoka T, Hosen N, Sonobe T, Arita Y, Yasui T, Masaki T, et al. Interleukin-6/interleukin-21 signaling axis is critical in the pathogenesis of pulmonary arterial hypertension. Proc Natl Acad Sci U S A. 2015;112(20):E2677–86. doi: 10.1073/pnas.1424774112 25941359 PMC4443312

[36] Abid S, Marcos E, Parpaleix A, Amsellem V, Breau M, Houssaini A, et al. CCR2/CCR5-mediated macrophage-smooth muscle cell crosstalk in pulmonary hypertension. Eur Respir J. 2019;54(4):1802308. doi: 10.1183/13993003.02308-2018 31320454

[37] Bissonnette EY, Lauzon-Joset J-F, Debley JS, Ziegler SF. Cross-talk between alveolar macrophages and lung epithelial cells is essential to maintain lung homeostasis. Front Immunol. 2020;11:583042. doi: 10.3389/fimmu.2020.583042 33178214 PMC7593577

[38] Westphalen K, Gusarova GA, Islam MN, Subramanian M, Cohen TS, Prince AS, et al. Sessile alveolar macrophages communicate with alveolar epithelium to modulate immunity. Nature. 2014;506(7489):503–6. doi: 10.1038/nature12902 24463523 PMC4117212

[39] Rabinovitch M, Guignabert C, Humbert M, Nicolls MR. Inflammation and immunity in the pathogenesis of pulmonary arterial hypertension. Circ Res. 2014;115(1):165–75. doi: 10.1161/CIRCRESAHA.113.301141 24951765 PMC4097142

[40] Zhang M-Q, Wang C-C, Pang X-B, Shi J-Z, Li H-R, Xie X-M, et al. Role of macrophages in pulmonary arterial hypertension. Front Immunol. 2023;14:1152881. doi: 10.3389/fimmu.2023.1152881 37153557 PMC10154553

[41] Tanaka T, Narazaki M, Kishimoto T. IL-6 in inflammation, immunity, and disease. Cold Spring Harb Perspect Biol. 2014;6(10):a016295. doi: 10.1101/cshperspect.a016295 25190079 PMC4176007

[42] Ambalavanan N, Carlo WA, D’Angio CT, McDonald SA, Das A, Schendel D, et al. Cytokines associated with bronchopulmonary dysplasia or death in extremely low birth weight infants. Pediatrics. 2009;123(4):1132–41. doi: 10.1542/peds.2008-0526 19336372 PMC2903210

[43] Dik WA, De Krijger RR, Bonekamp L, Naber BA, Zimmermann LJ, Versnel MA. Localization and potential role of matrix metalloproteinase-1 and tissue inhibitors of metalloproteinase-1 and -2 in different phases of bronchopulmonary dysplasia. Pediatr Res. 2001;50(6):761–6. doi: 10.1203/00006450-200112000-00022 11726737

[44] Parthasarathi K, Ichimura H, Monma E, Lindert J, Quadri S, Issekutz A, et al. Connexin 43 mediates spread of Ca2+-dependent proinflammatory responses in lung capillaries. J Clin Invest. 2006;116(8):2193–200. doi: 10.1172/JCI26605 16878174 PMC1518791

[45] Shoemaker CT, Reiser KM, Goetzman BW, Last JA. Elevated ratios of type I/III collagen in the lungs of chronically ventilated neonates with respiratory distress. Pediatr Res. 1984;18(11):1176–80. doi: 10.1203/00006450-198411000-00025 6514444

[46] Watts CL, Fanaroff AA, Bruce MC. Elevation of fibronectin levels in lung secretions of infants with respiratory distress syndrome and development of bronchopulmonary dysplasia. J Pediatr. 1992;120(4 Pt 1):614–20. doi: 10.1016/s0022-3476(05)82492-8 1552403

[47] Jin R, Xu J, Gao Q, Mao X, Yin J, Lu K, et al. IL-33-induced neutrophil extracellular traps degrade fibronectin in a murine model of bronchopulmonary dysplasia. Cell Death Discov. 2020;6:33. doi: 10.1038/s41420-020-0267-2 32377396 PMC7198621

[48] Chen C, Weng H, Zhang X, Wang S, Lu C, Jin H, et al. Low-dose vitamin D protects hyperoxia-induced bronchopulmonary dysplasia by inhibiting neutrophil extracellular traps. Front Pediatr. 2020;8:335. doi: 10.3389/fped.2020.00335 32719755 PMC7347751

[49] Sun Y, Chen C, Zhang X, Wang S, Zhu R, Zhou A, et al. Heparin improves alveolarization and vascular development in hyperoxia-induced bronchopulmonary dysplasia by inhibiting neutrophil extracellular traps. Biochem Biophys Res Commun. 2020;522(1):33–9. doi: 10.1016/j.bbrc.2019.11.041 31735330

[50] O’Reilly M, Thébaud B. Animal models of bronchopulmonary dysplasia. The term rat models. Am J Physiol Lung Cell Mol Physiol. 2014;307(12):L948–58. doi: 10.1152/ajplung.00160.2014 25305248

[51] Yam J, Frank L, Roberts RJ. Oxygen toxicity: comparison of lung biochemical responses in neonatal and adult rats. Pediatr Res. 1978;12(2):115–9. doi: 10.1203/00006450-197802000-00010 643379

