# Supplementary material for: Role of connexin 43 in hyperoxia-induced bronchopulmonary dysplasia and associated pulmonary hypertension
Source: PLoS One. 2026 Aug 24;21(8):e0356364. doi: 10.1371/journal.pone.0356364 (PMC13502688; doi:10.1371/journal.pone.0356364)
Supplement: Supplementary file 1 [file pone.0356364.s006.pdf]

ORIGINAL BLOT AND GEL IMAGES

FIGURE 1A

BPD-PH animal model

Connexin 43 GEL

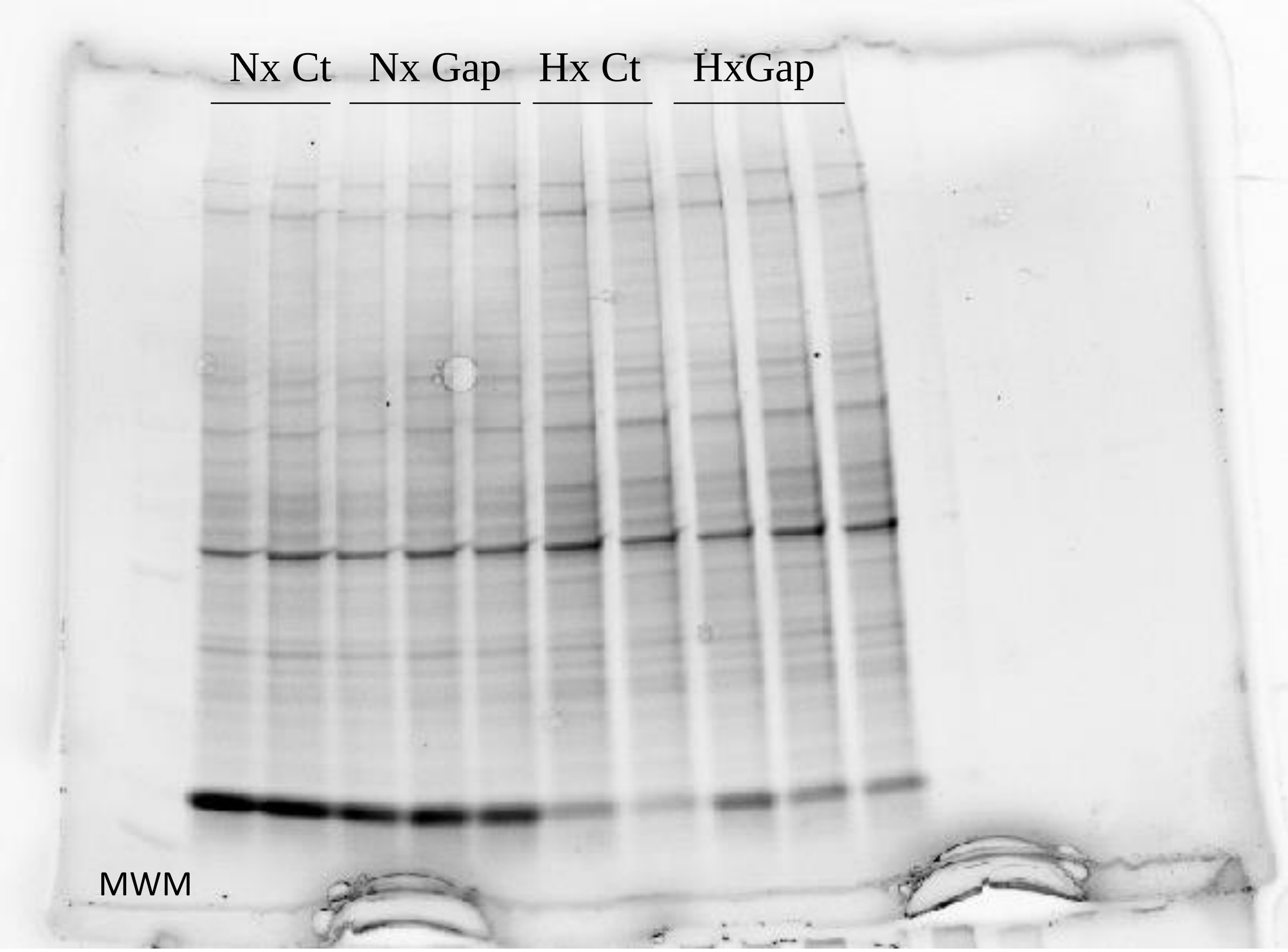

Whole lung

MWM : molecular weight markers

FIGURE 1A

BPD-PH animal model

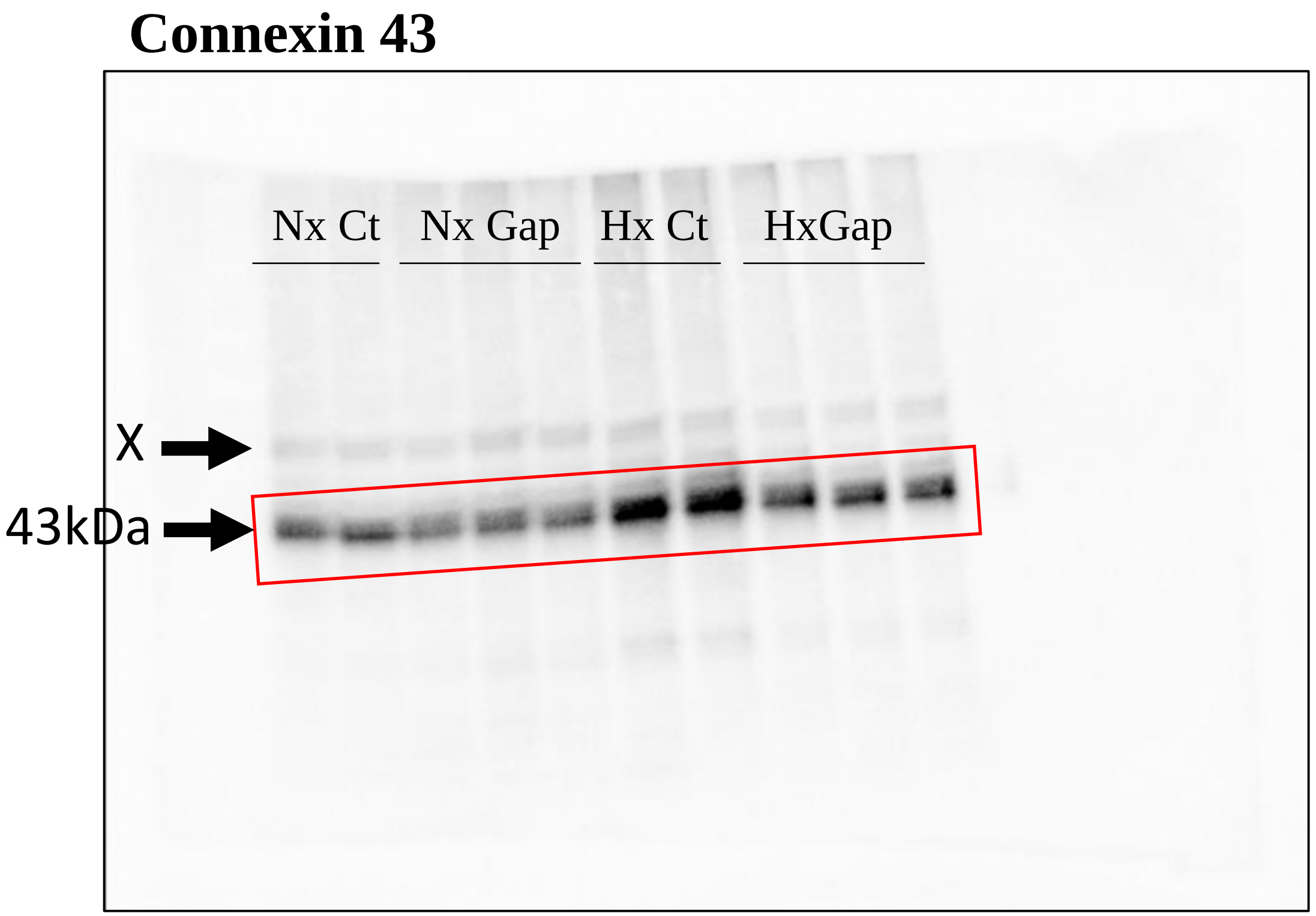

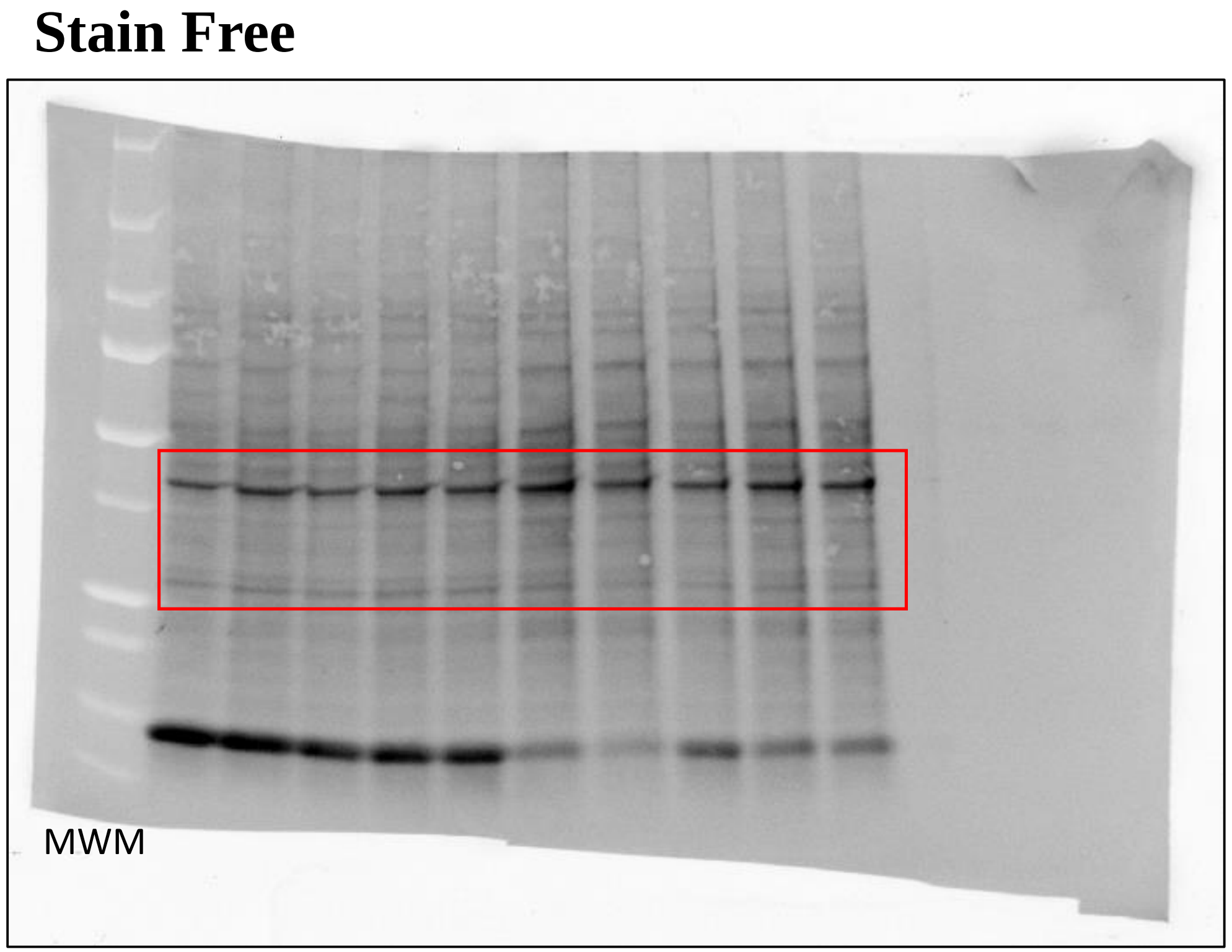

Whole lung

MWM : molecular weight markers

FIGURE 1B

BPD-PH animal model

Connexin 43 GEL

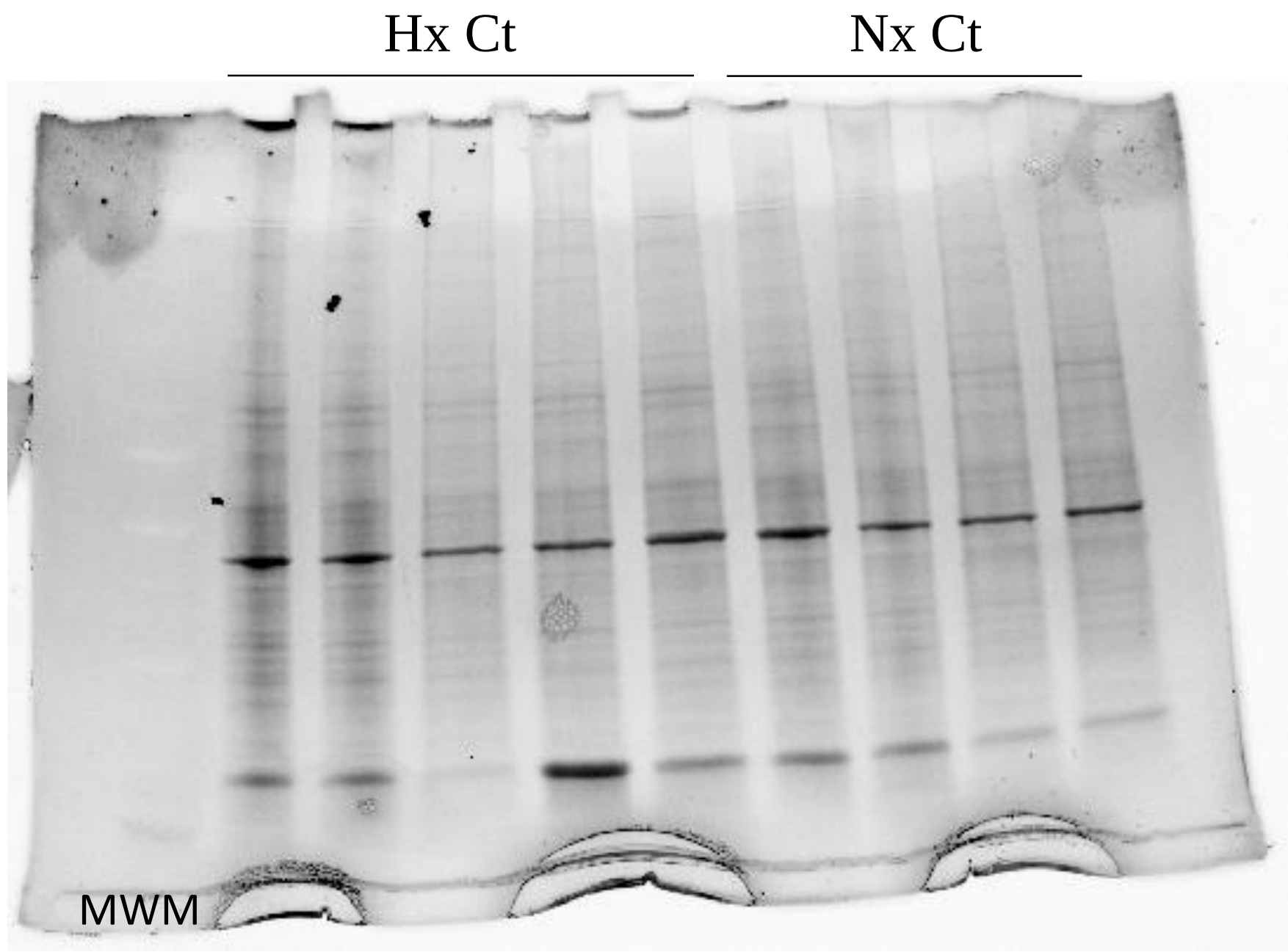

Intrapulmonary arteries

MWM : molecular weight markers

FIGURE 1B

BPD-PH animal model

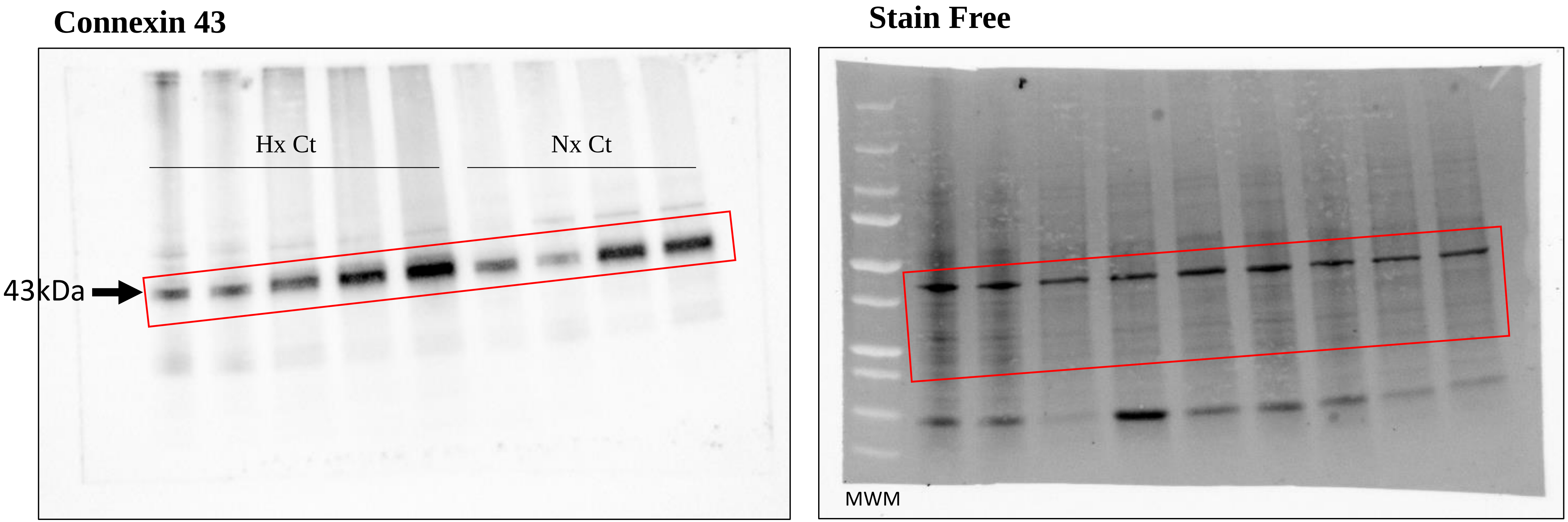

Intrapulmonary arteries

MWM : molecular weight markers

FIGURE 1C

<u>HfPA-SMC</u>

Connexin 43 GEL

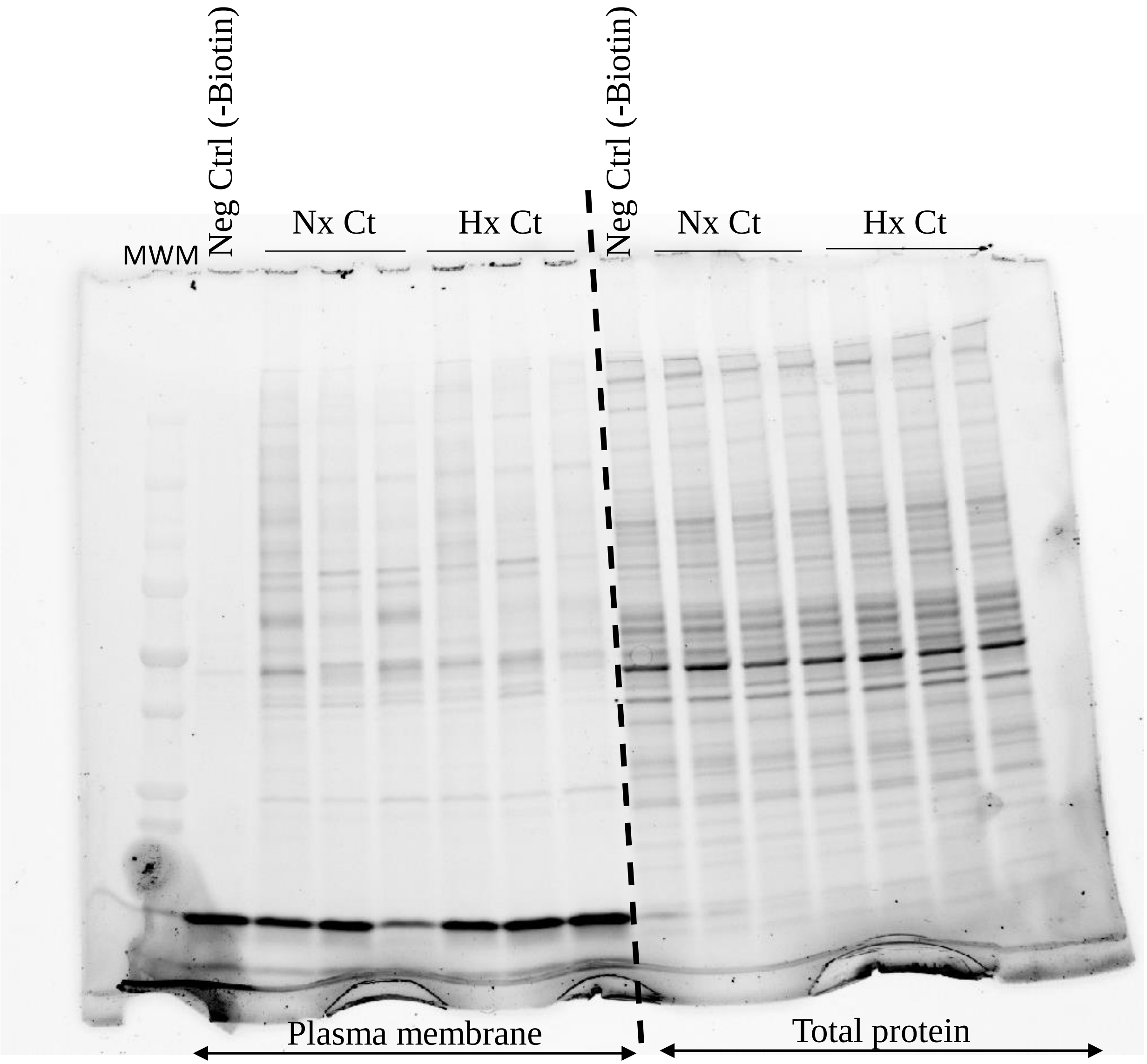

MWM : molecular weight markers

FIGURE 1C

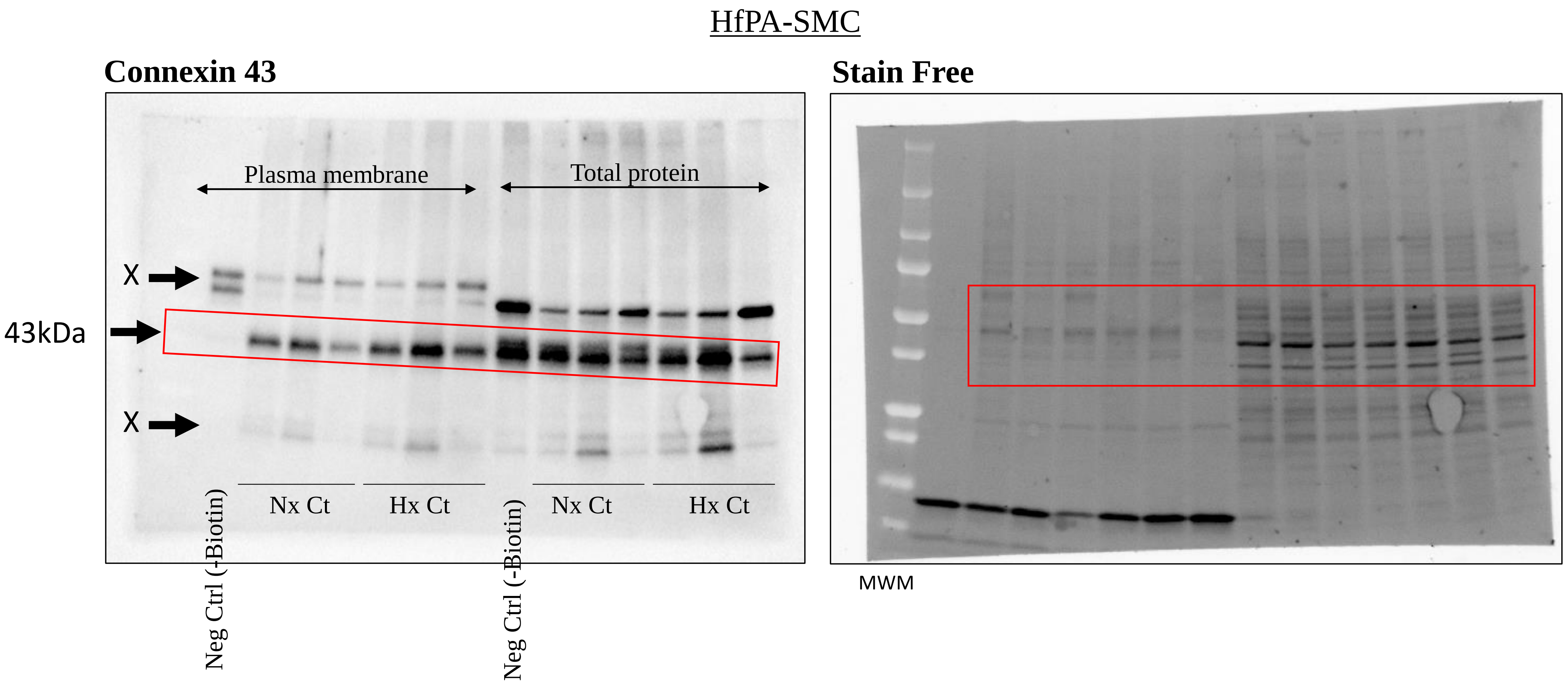

MWM : molecular weight markers

FIGURE 3A

BPD-PH animal model

RT1-40 GEL

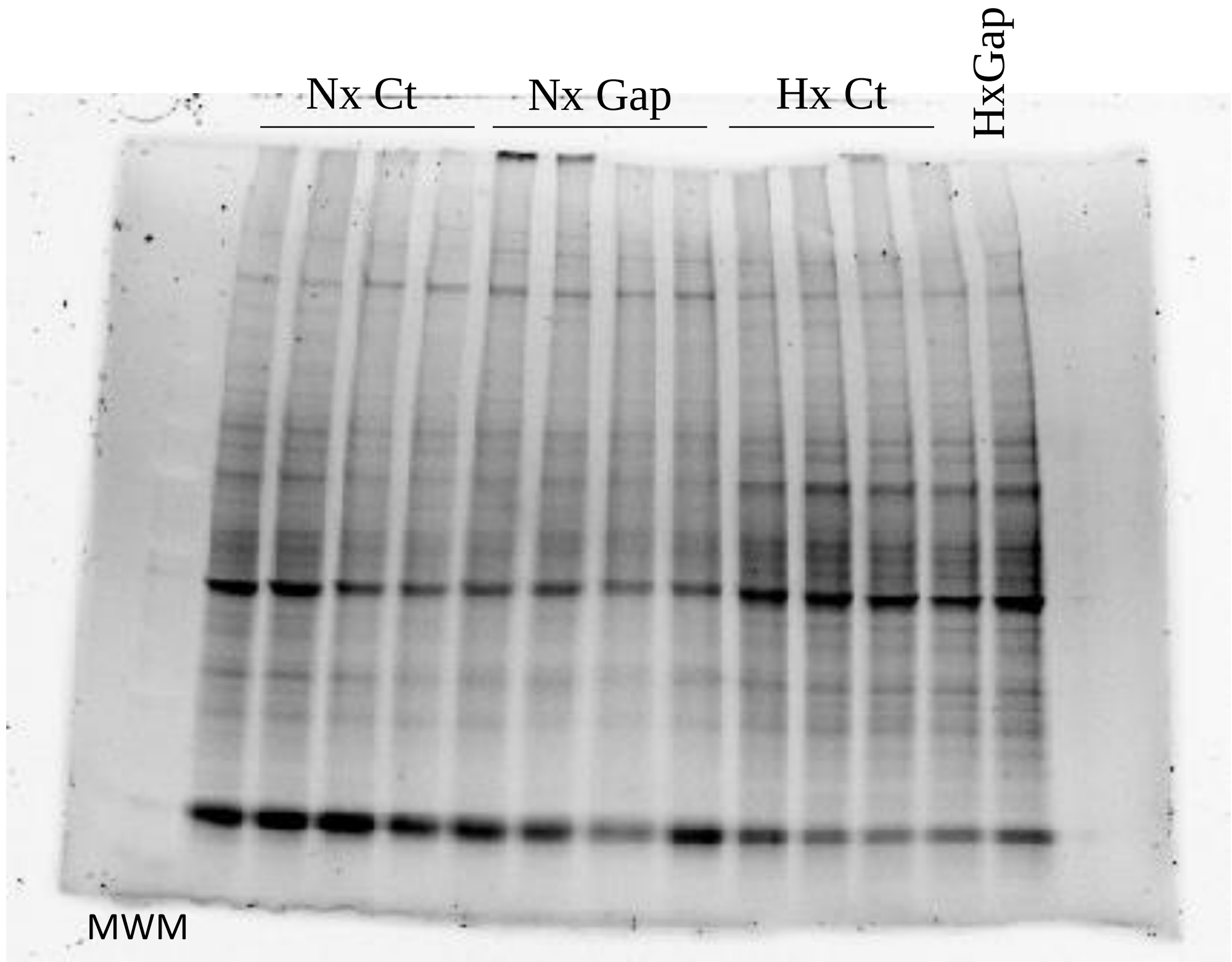

MWM : molecular weight markers

FIGURE 3A

BPD-PH animal model

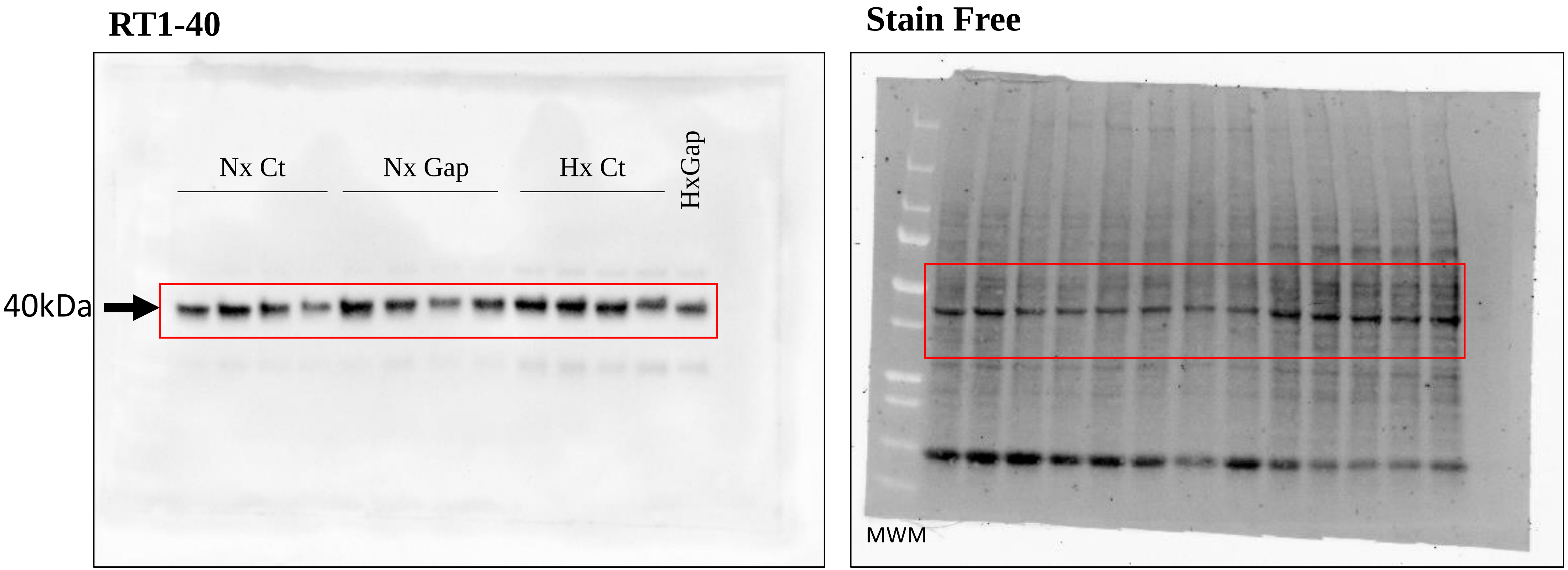

MWM : molecular weight markers

FIGURE 3B

BPD-PH animal model

SP-C GEL

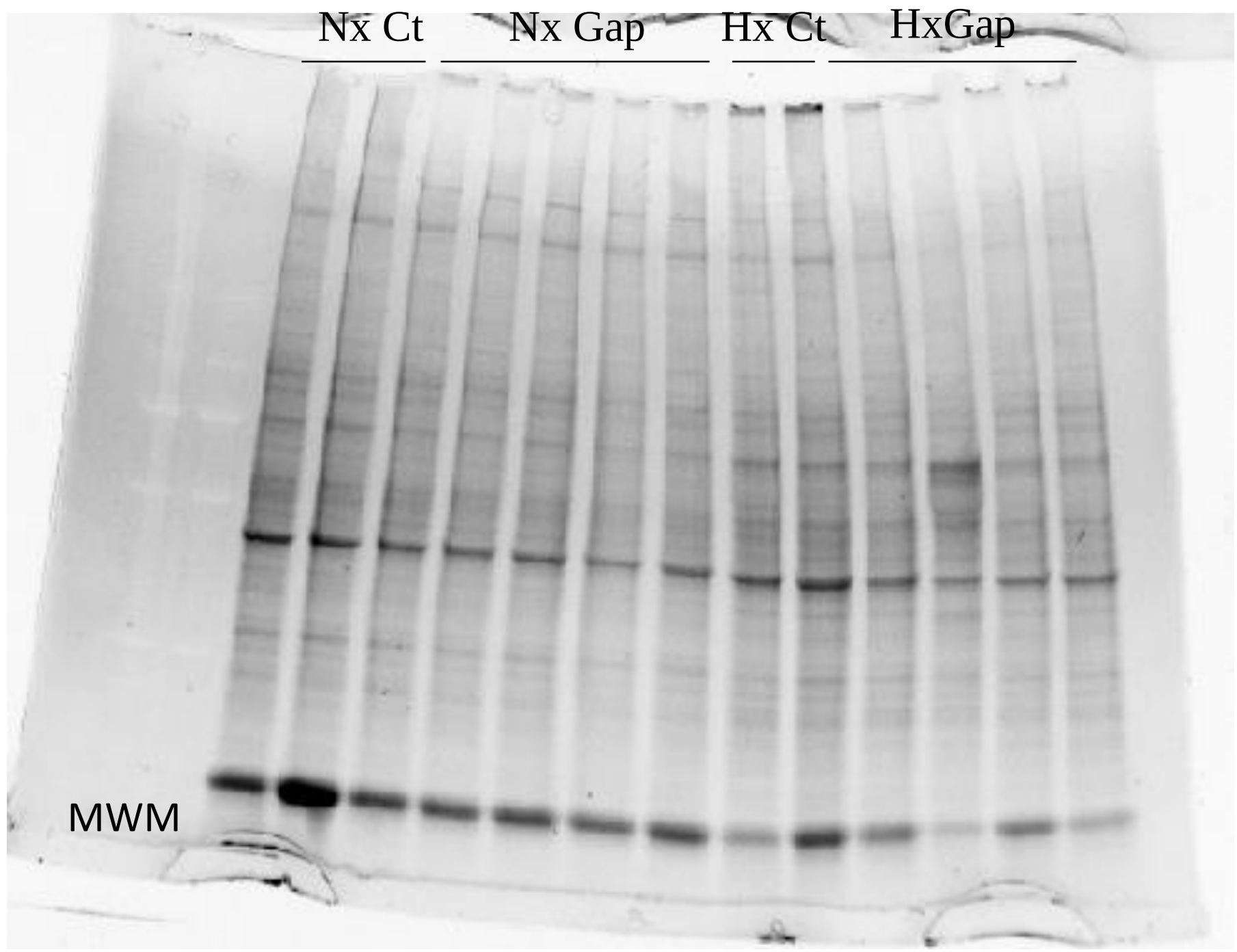

MWM : molecular weight markers

FIGURE 3B

BPD-PH animal model

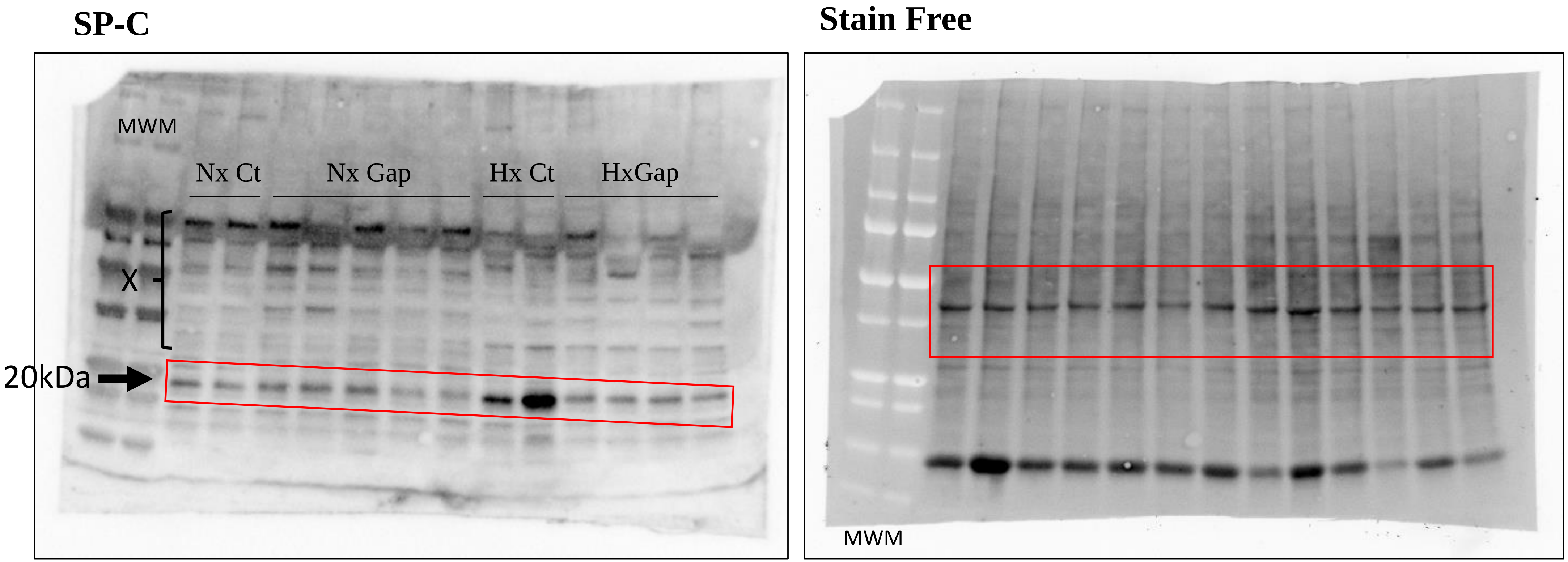

MWM : molecular weight markers

FIGURE 3D

BPD-PH animal model

SP-B GEL

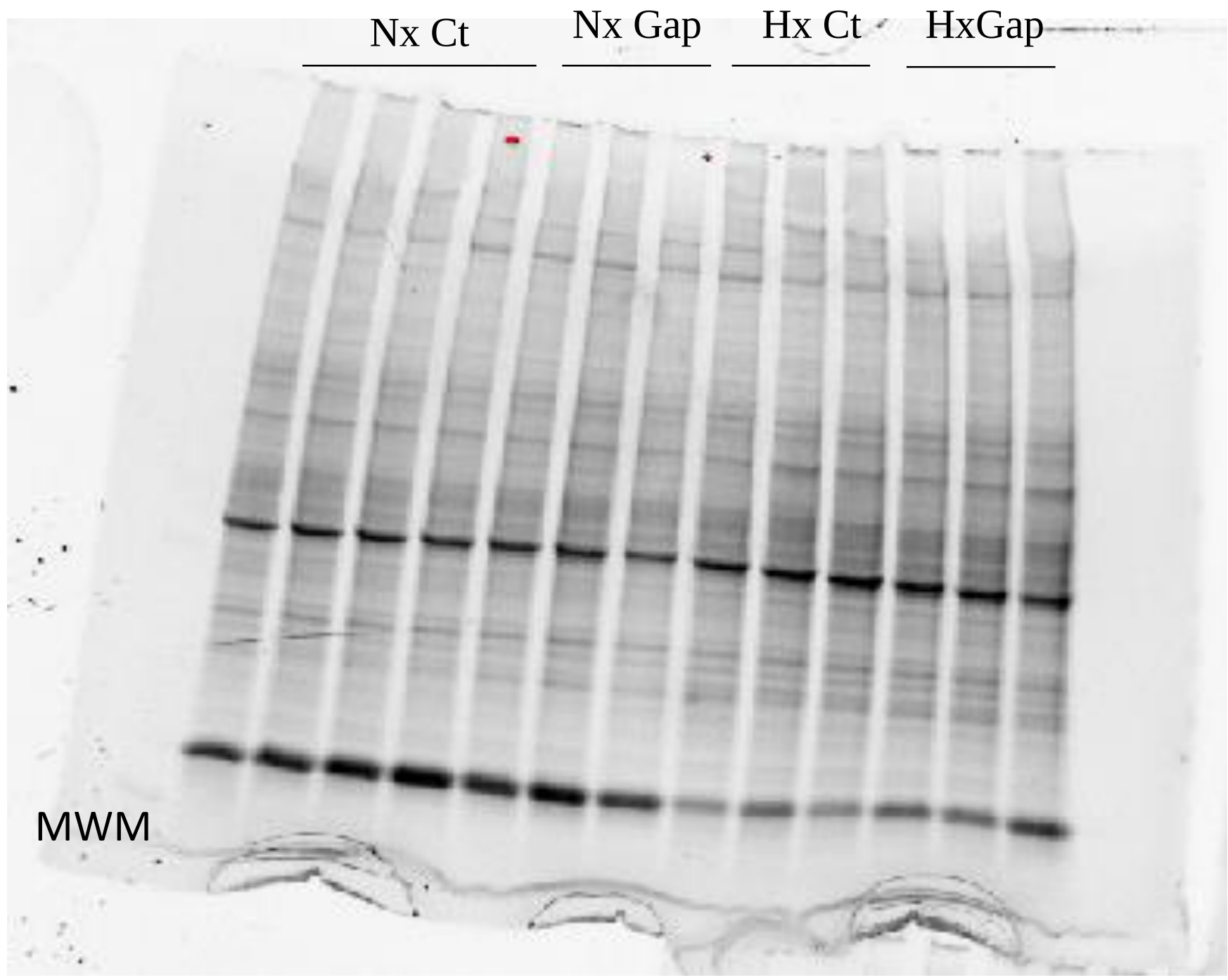

MWM : molecular weight markers

FIGURE 3D

BPD-PH animal model

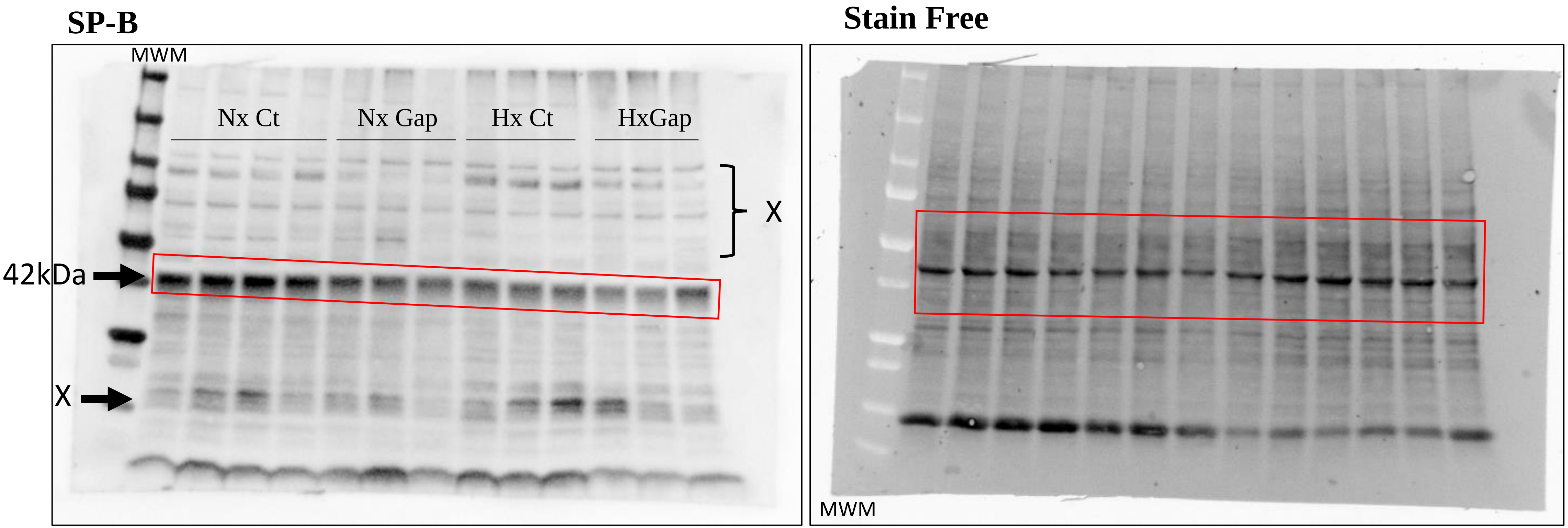

MWM : molecular weight markers

FIGURE 4A

BPD-PH animal model

CD68 GEL

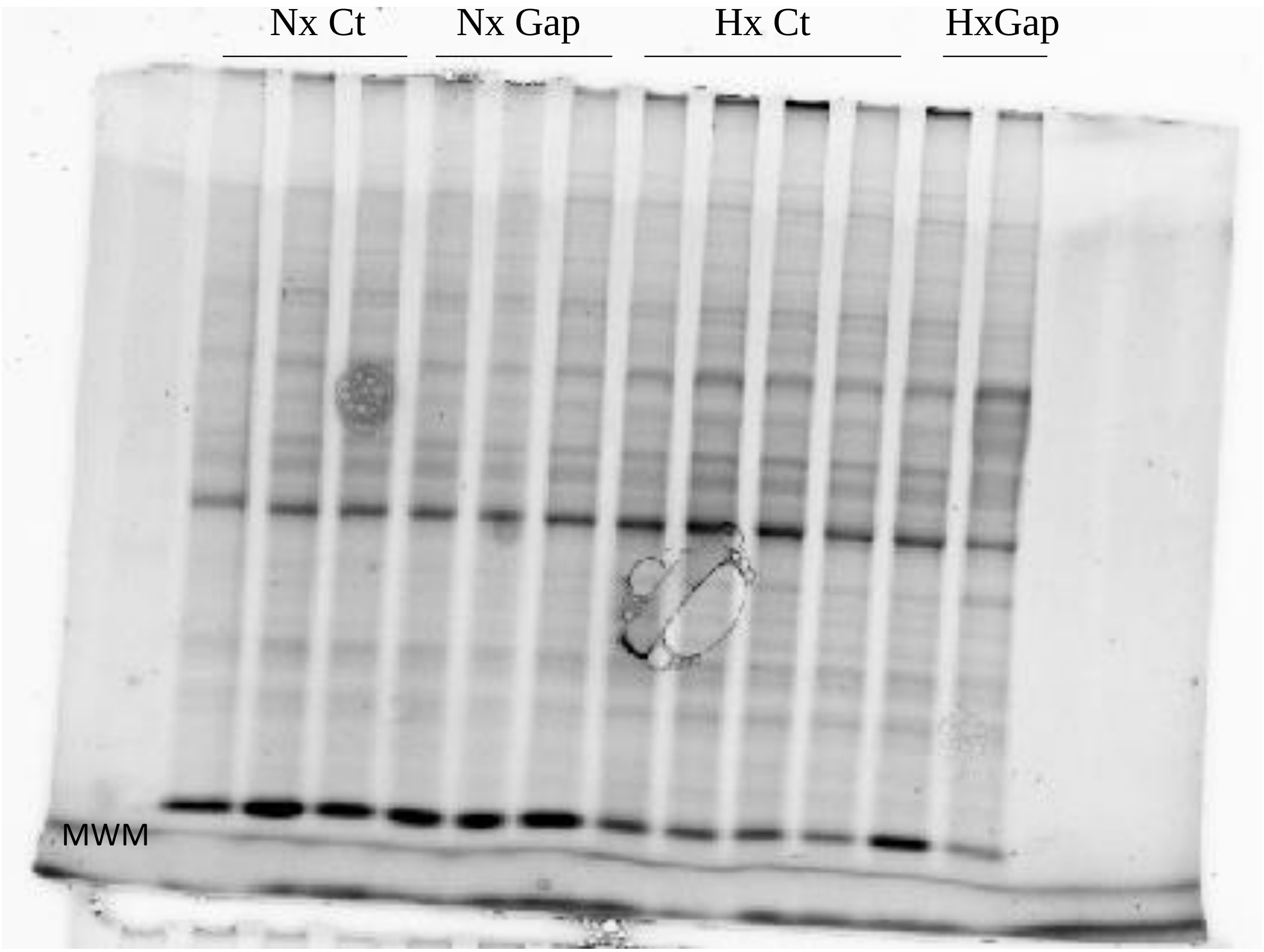

MWM : molecular weight markers

FIGURE 4A

BPD-PH animal model

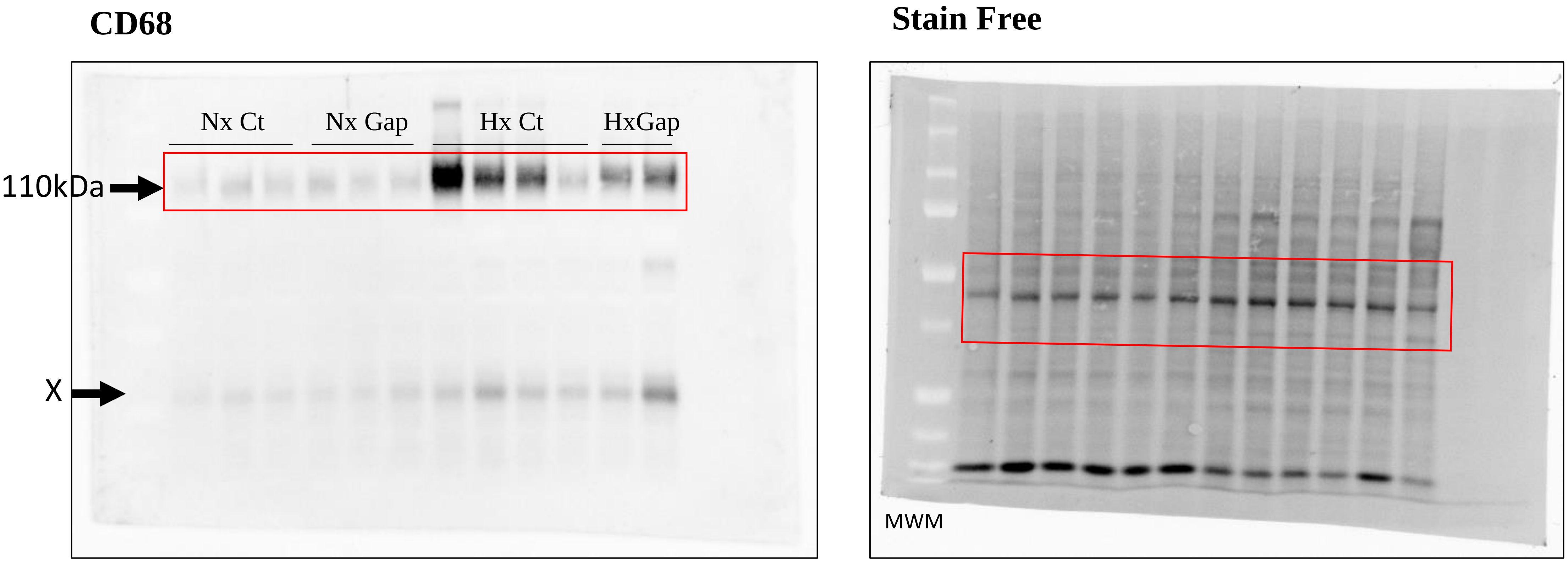

MWM : molecular weight markers

FIGURE 4B

BPD-PH animal model

CD86 GEL

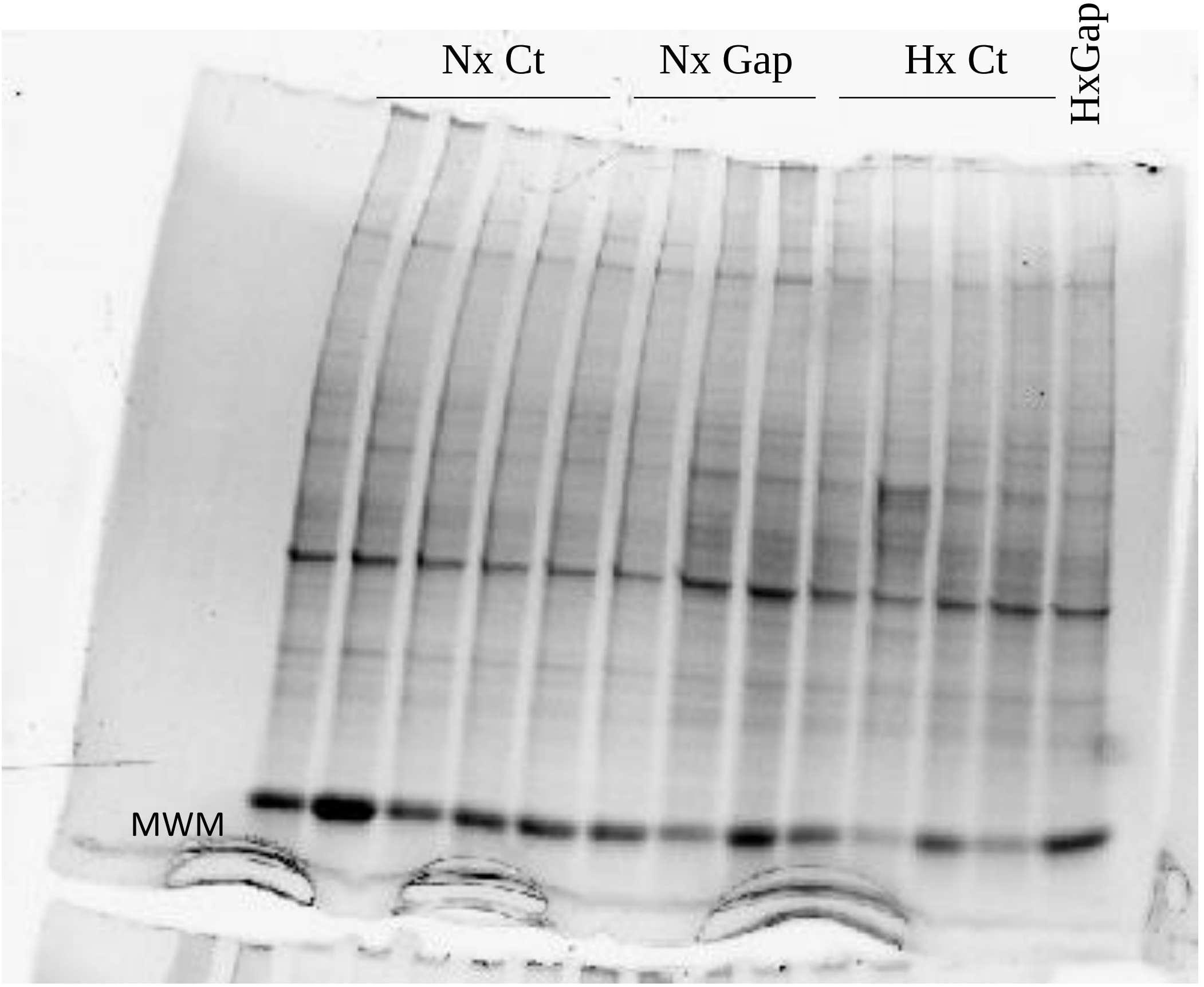

MWM : molecular weight markers

FIGURE 4B

BPD-PH animal model

CD86

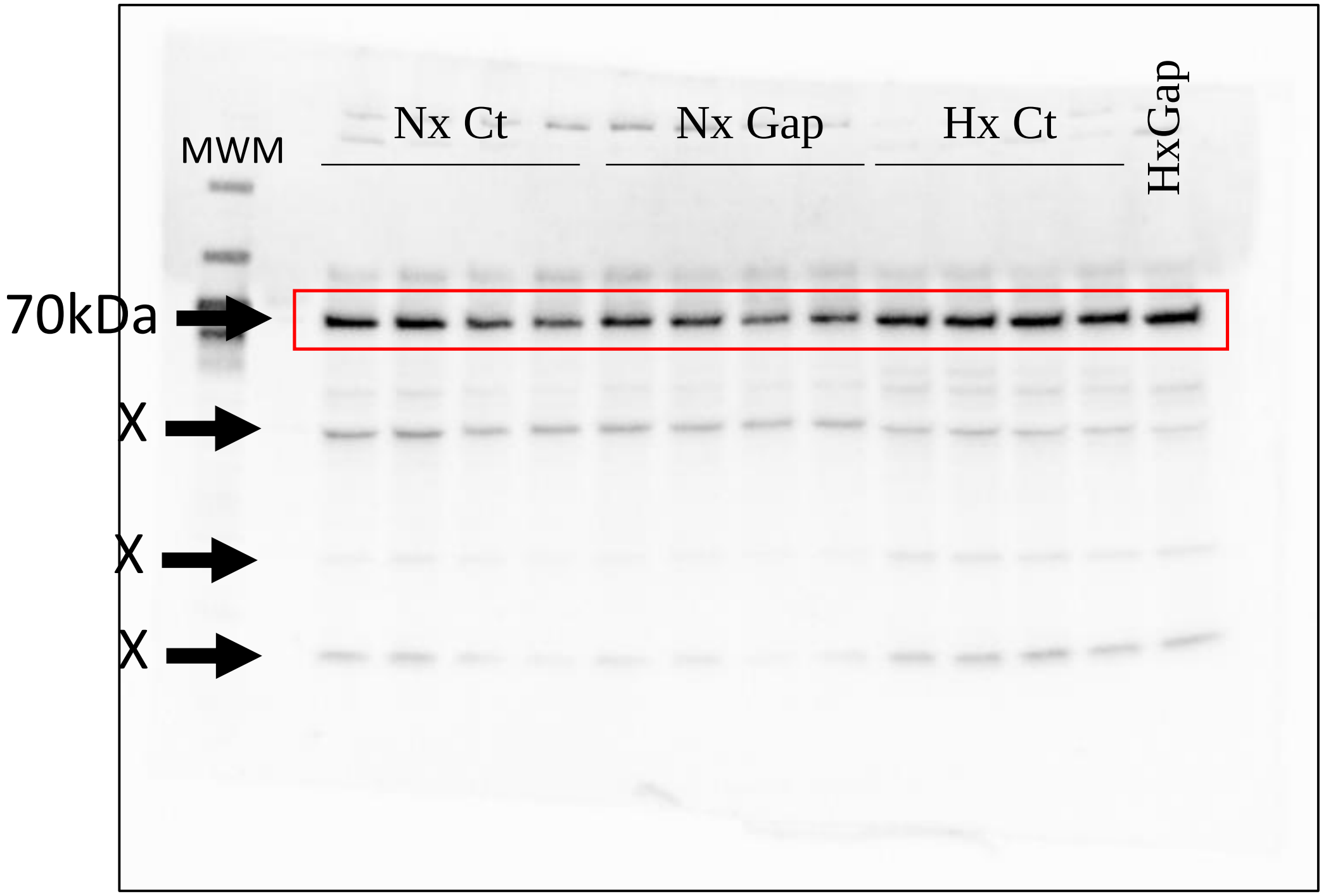

Stain Free

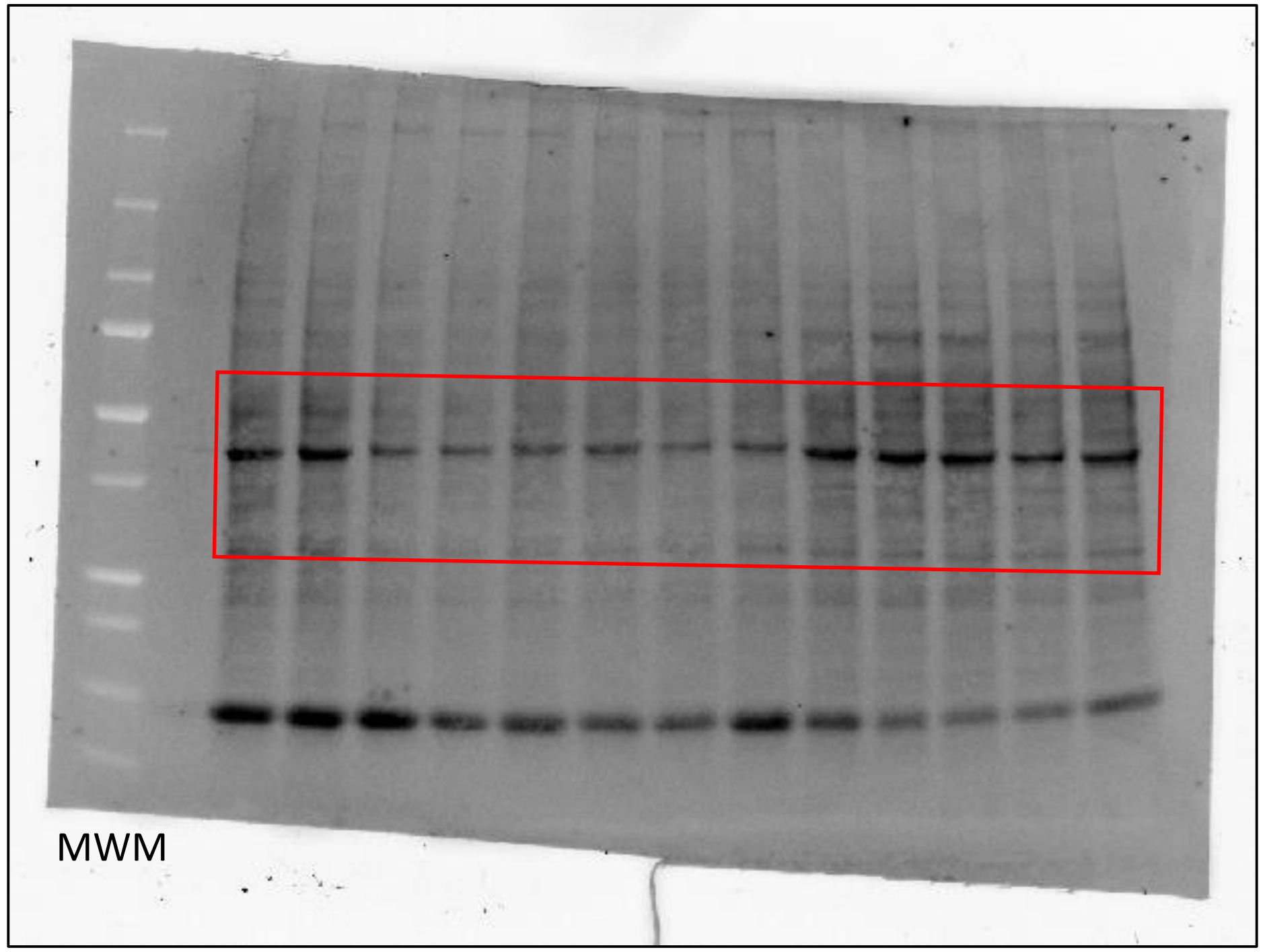

MWM : molecular weight markers

FIGURE 4C

BPD-PH animal model

CD206 GEL

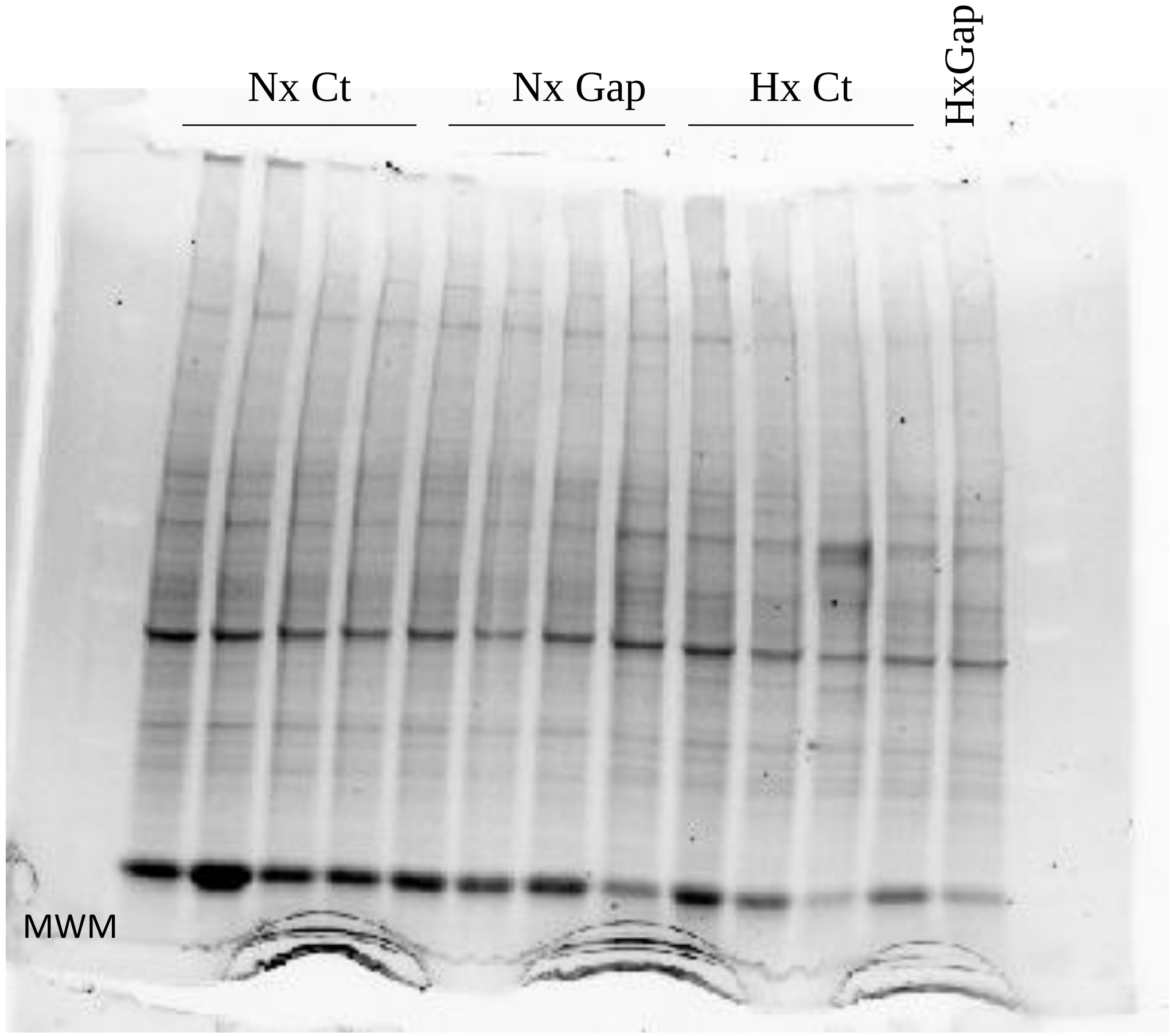

MWM : molecular weight markers

FIGURE 4C

BPD-PH animal model

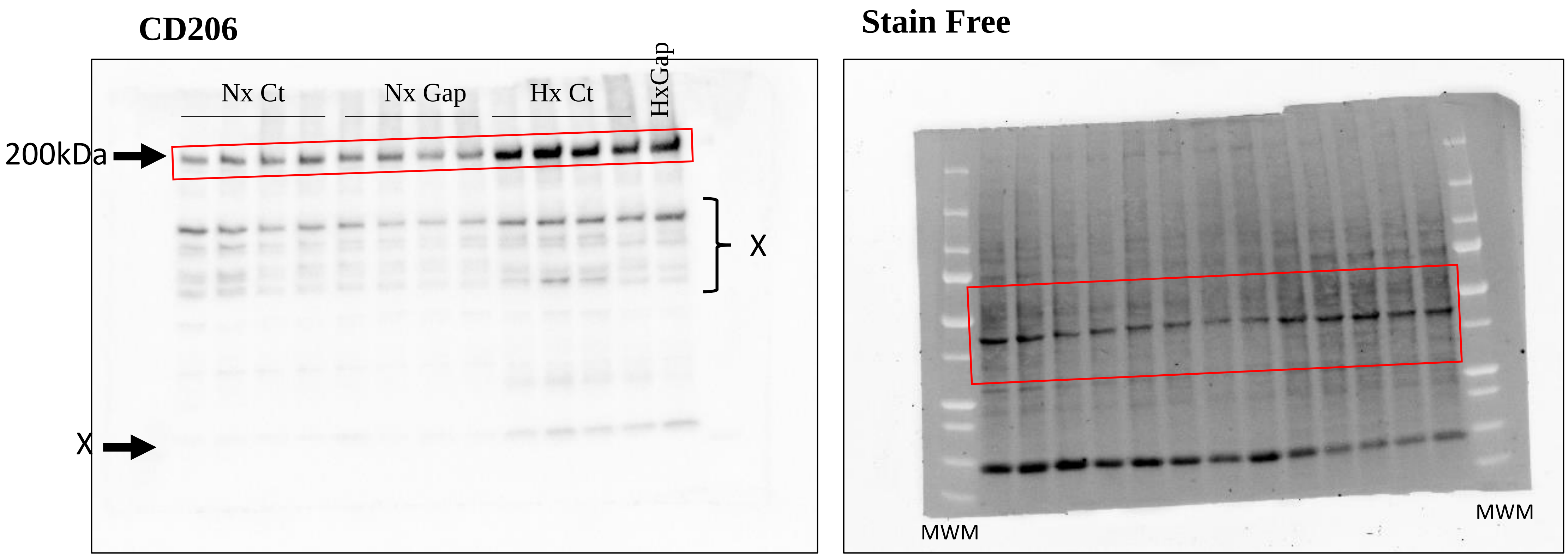

MWM : molecular weight markers

FIGURE 5A

BPD-PH animal model

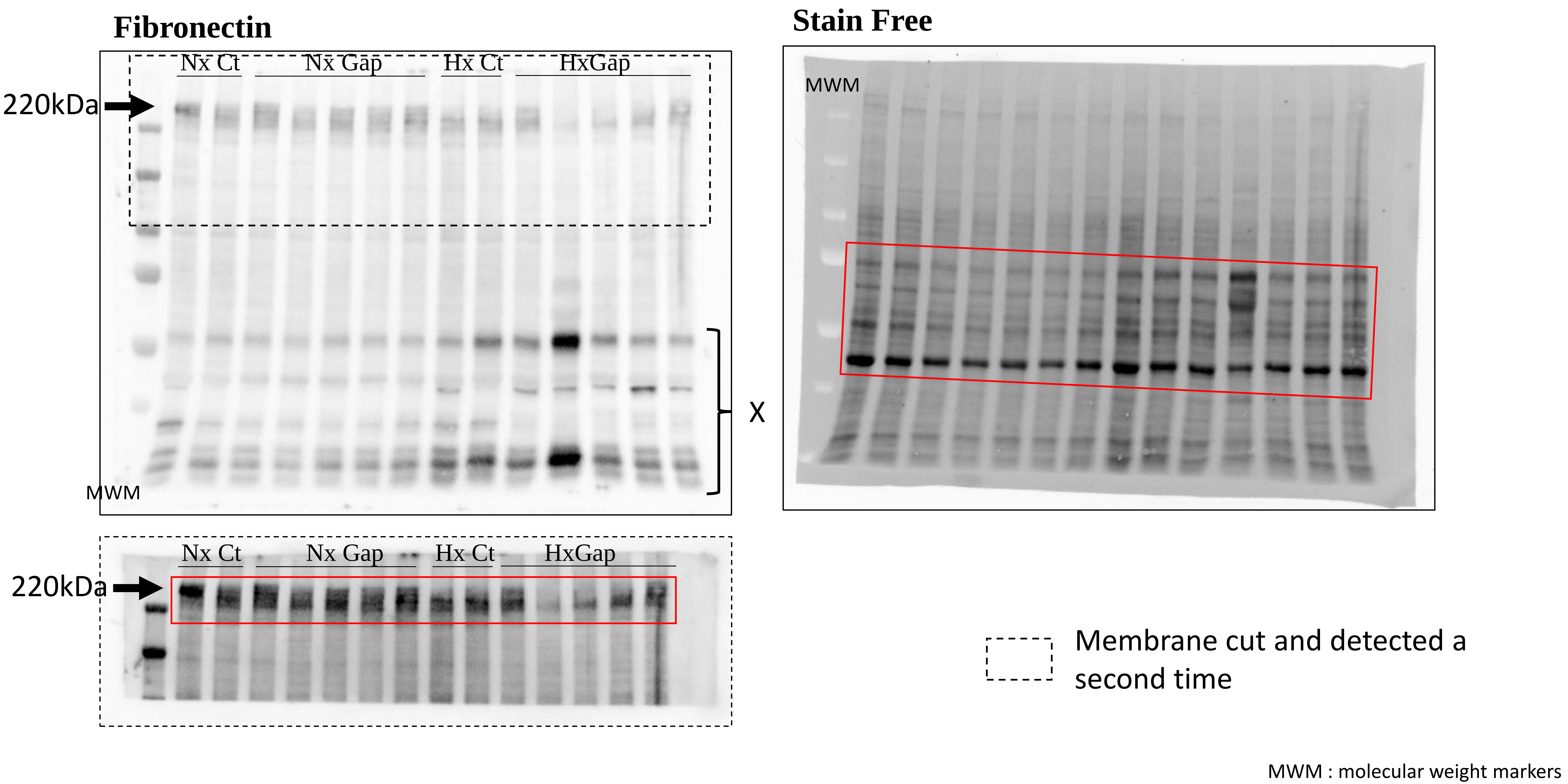

FIGURE 5A

BPD-PH animal model

Fibronectin

Nx Ct Nx Gap Hx Ct HxGap MWM

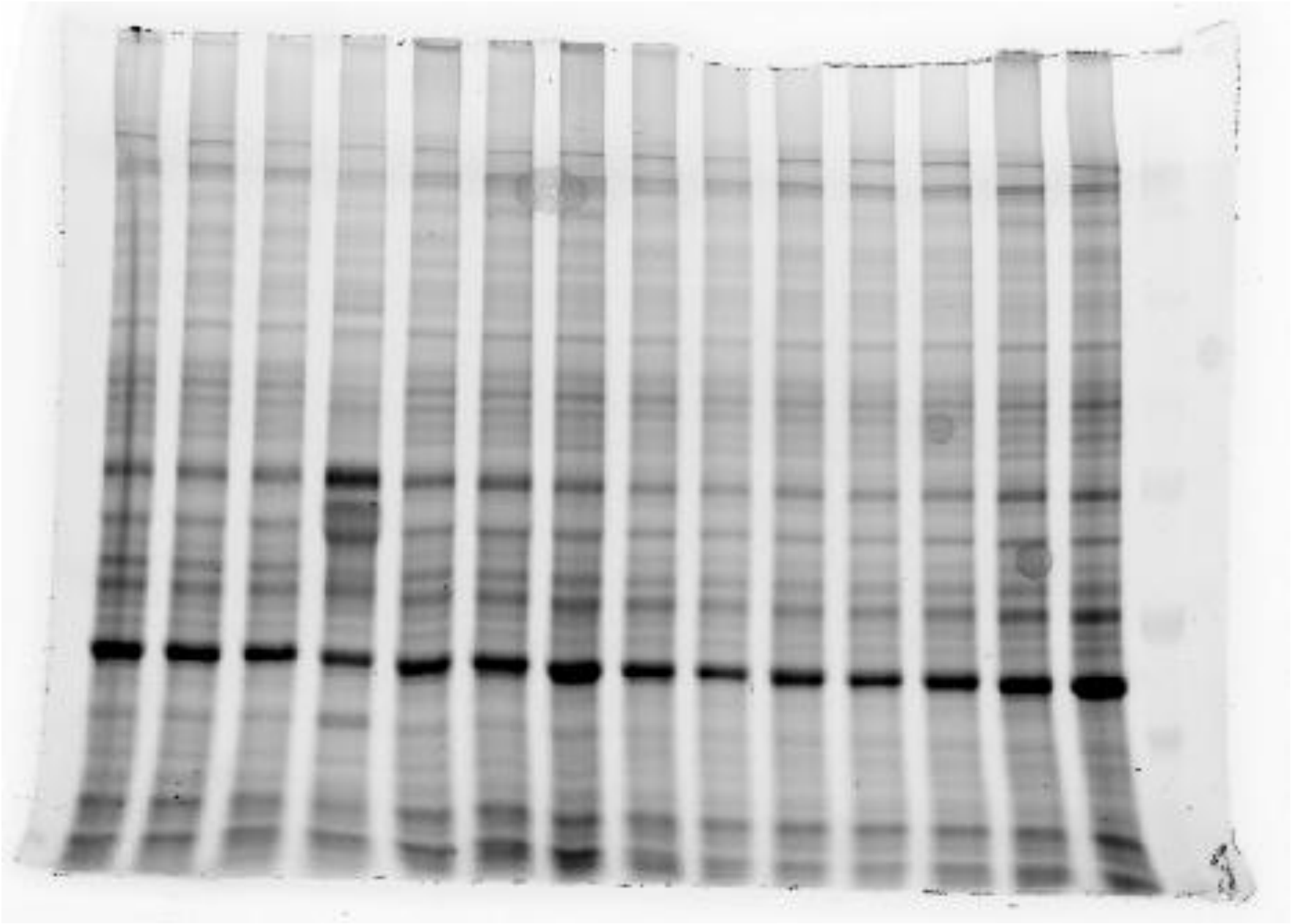

MWM : molecular weight markers

FIGURE 5B

BPD-PH animal model

Col1a GEL

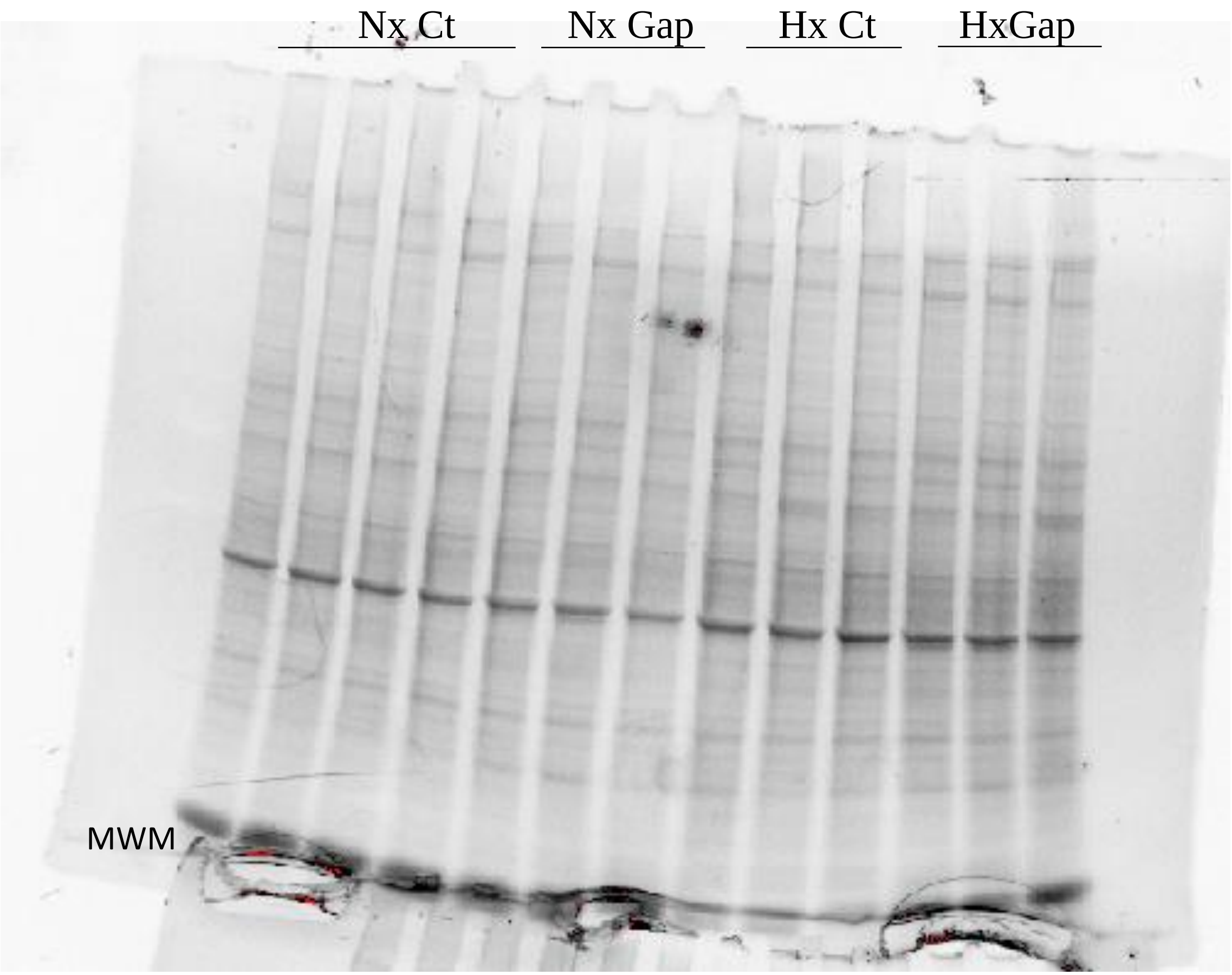

MWM : molecular weight markers

FIGURE 5B

BPD-PH animal model

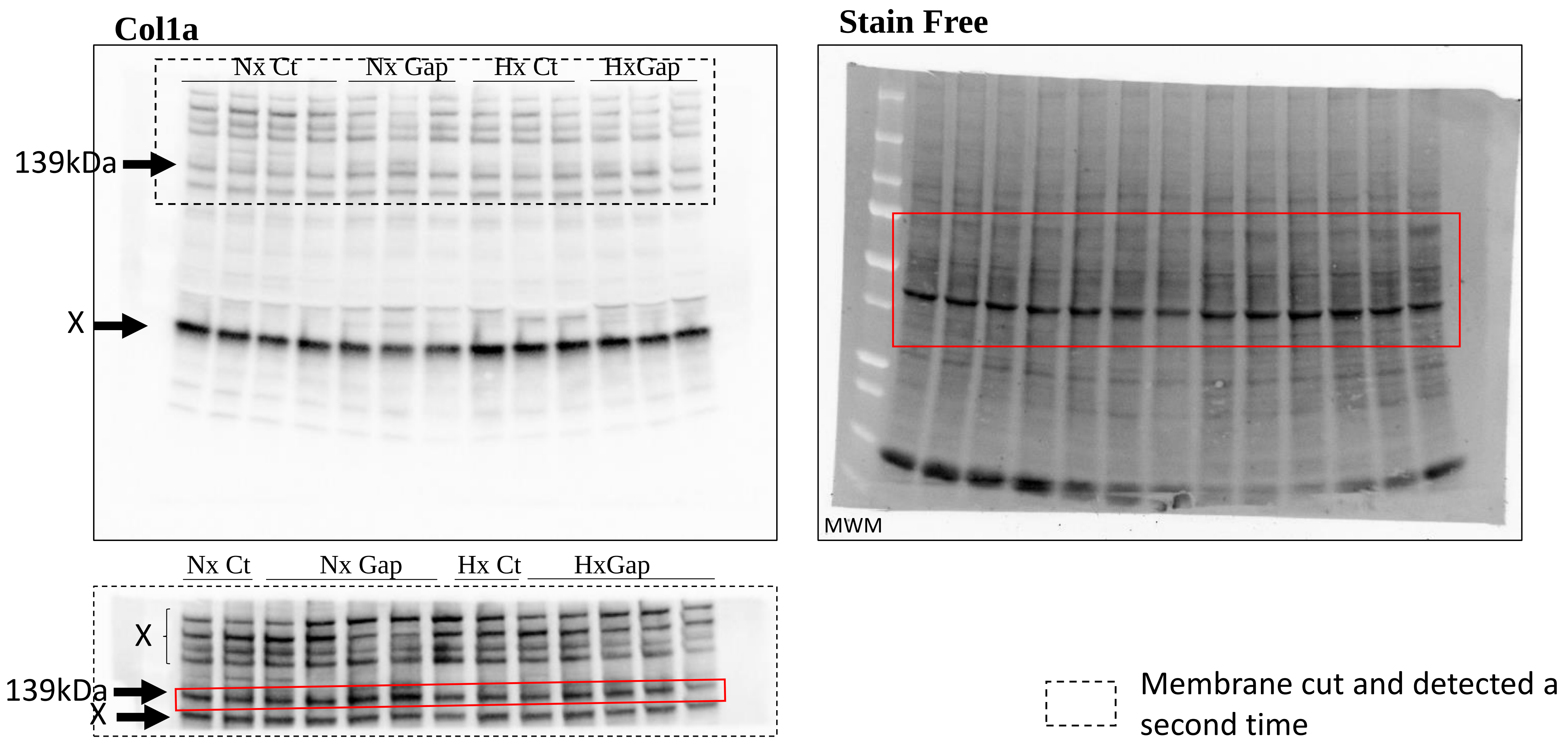

MWM : molecular weight markers

FIGURE 5C

BPD-PH animal model

Col3 GEL

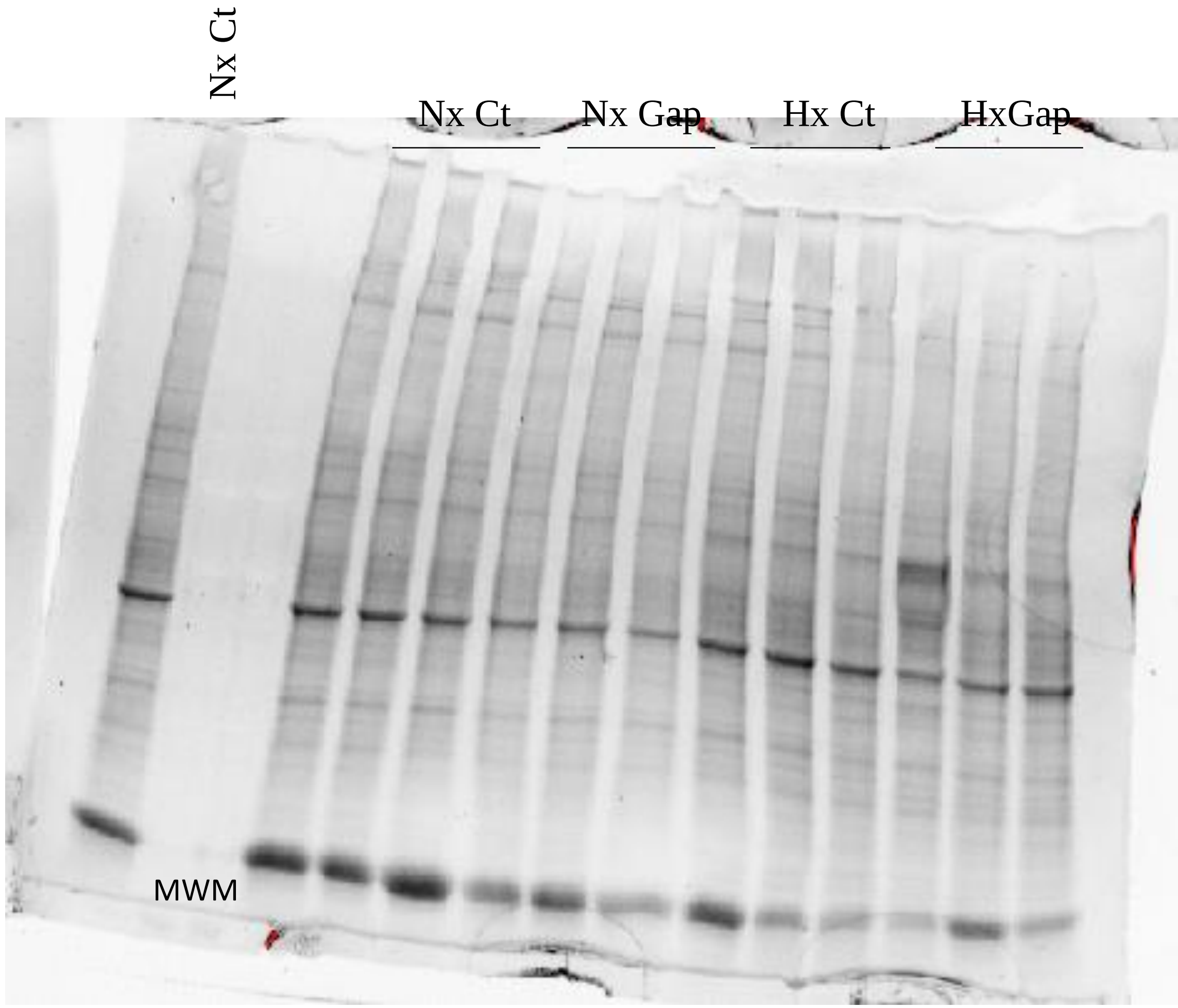

MWM : molecular weight markers

FIGURE 5C

BPD-PH animal model

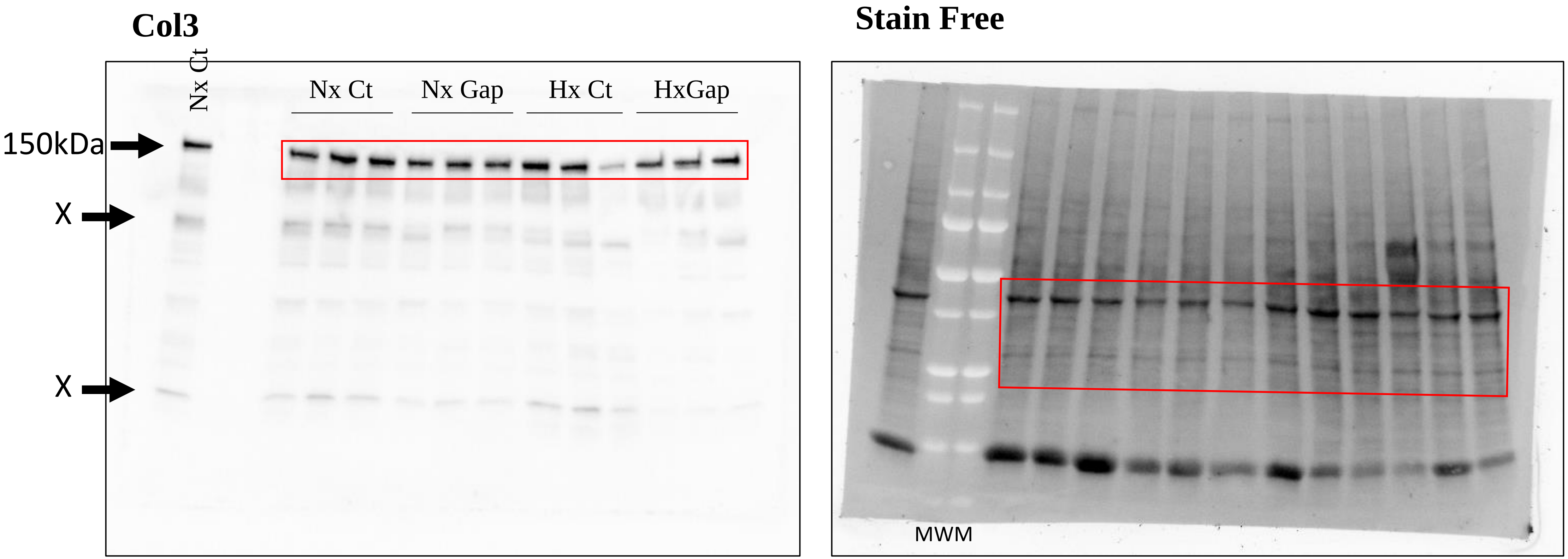

MWM : molecular weight markers

FIGURE S3A

BPD-PH animal model

4-HNE GEL

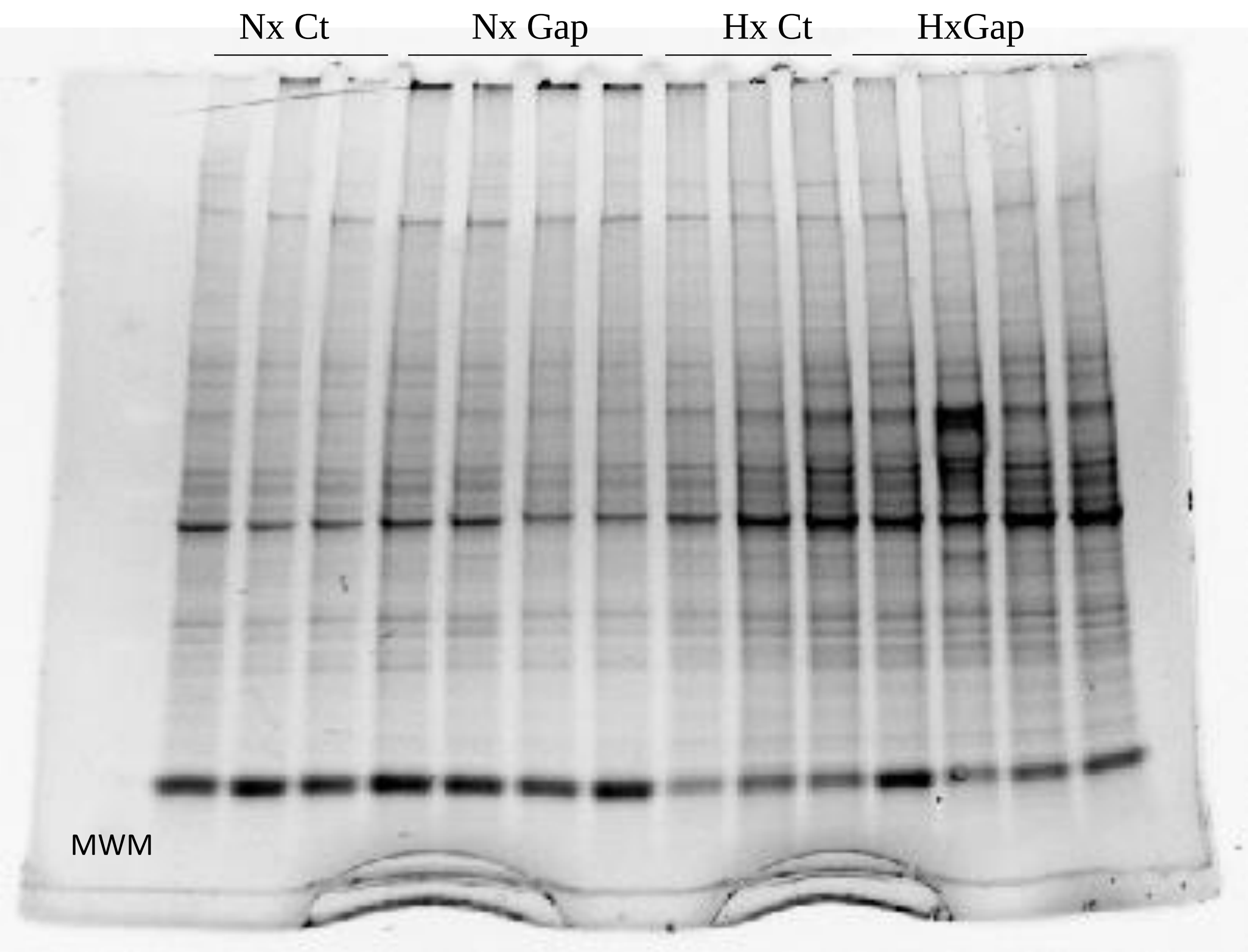

MWM : molecular weight markers

FIGURE S3A

BPD-PH animal model

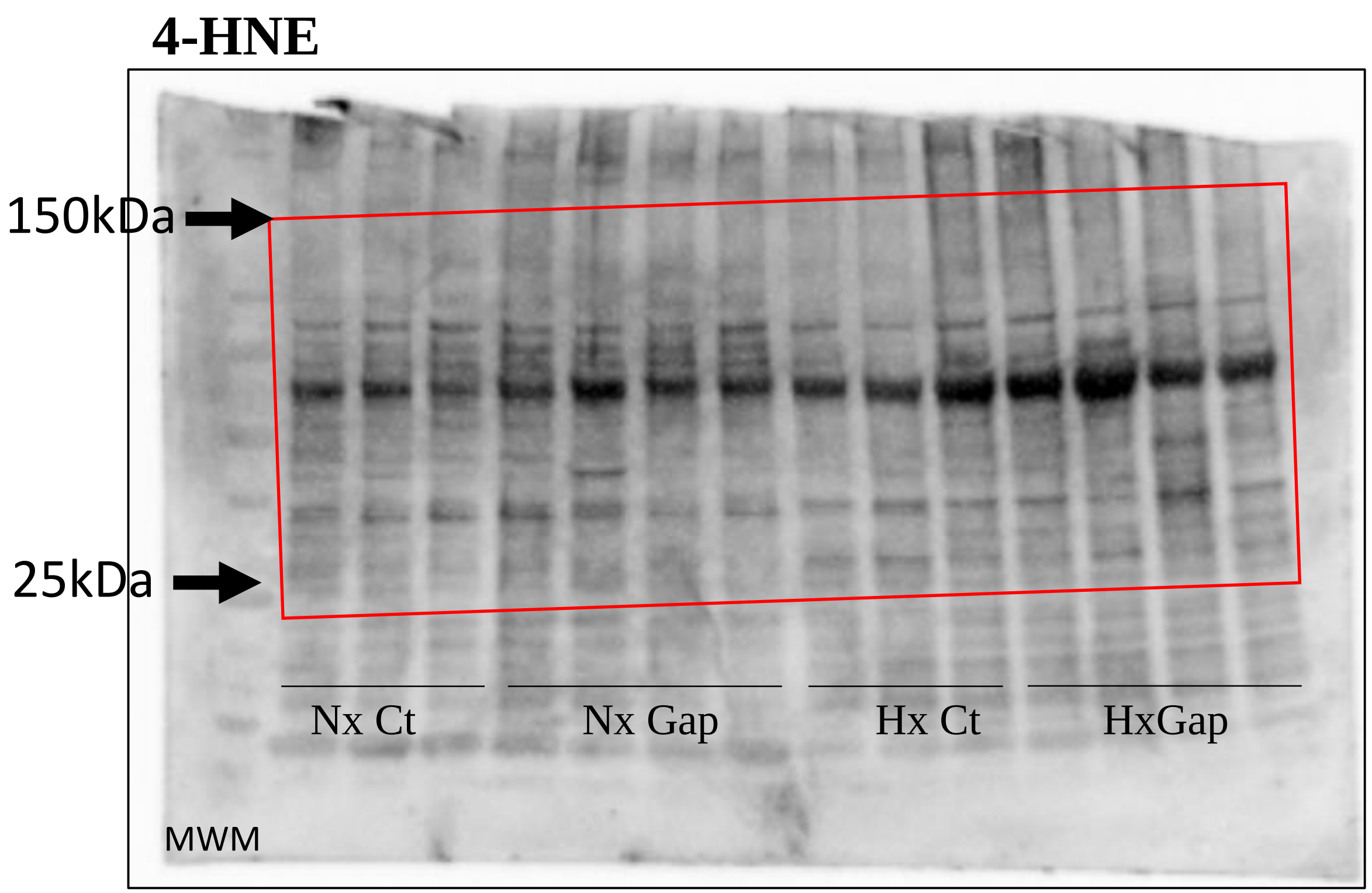

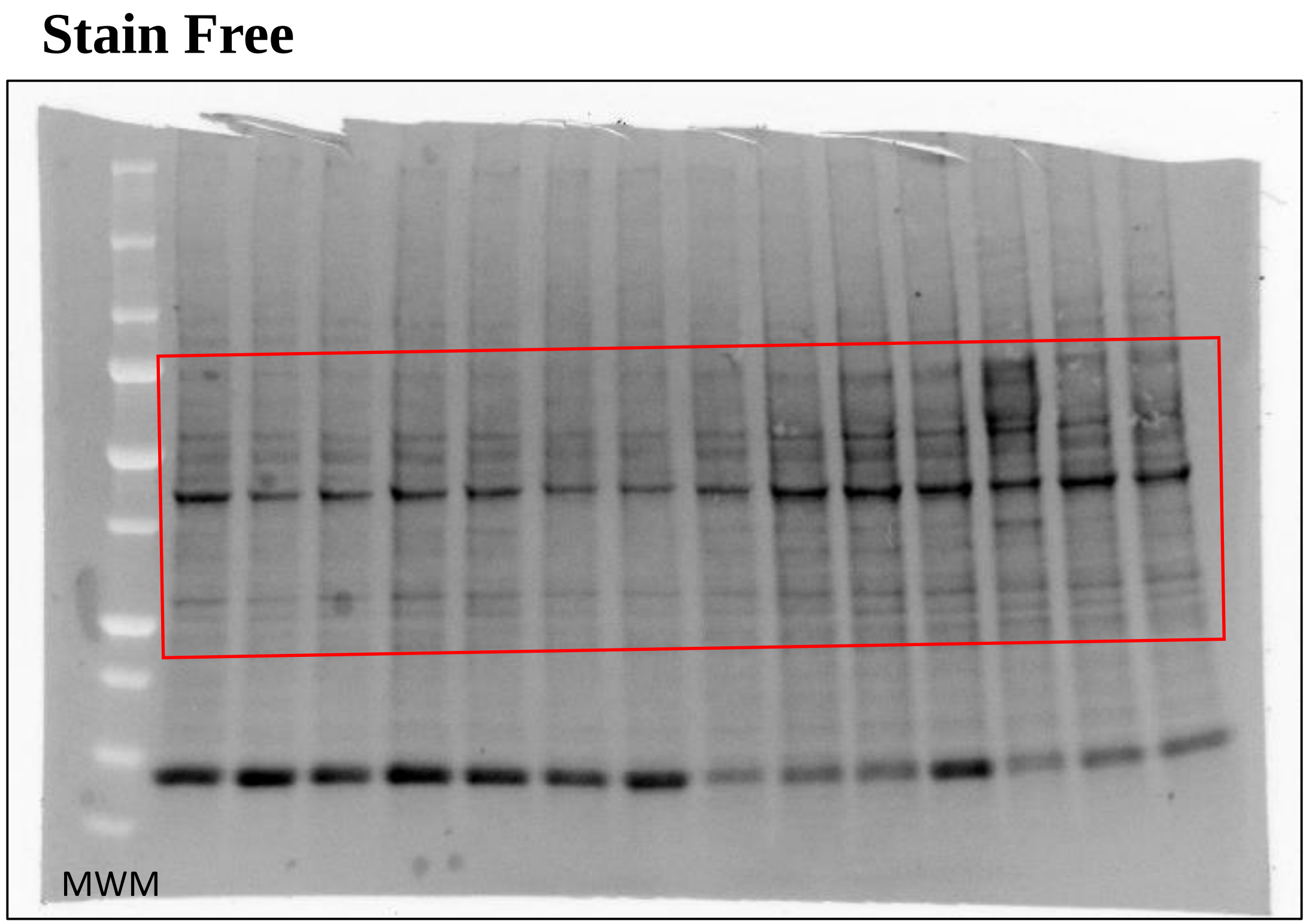

MWM : molecular weight markers

FIGURE S4B

BPD-PH animal model

DNP GEL

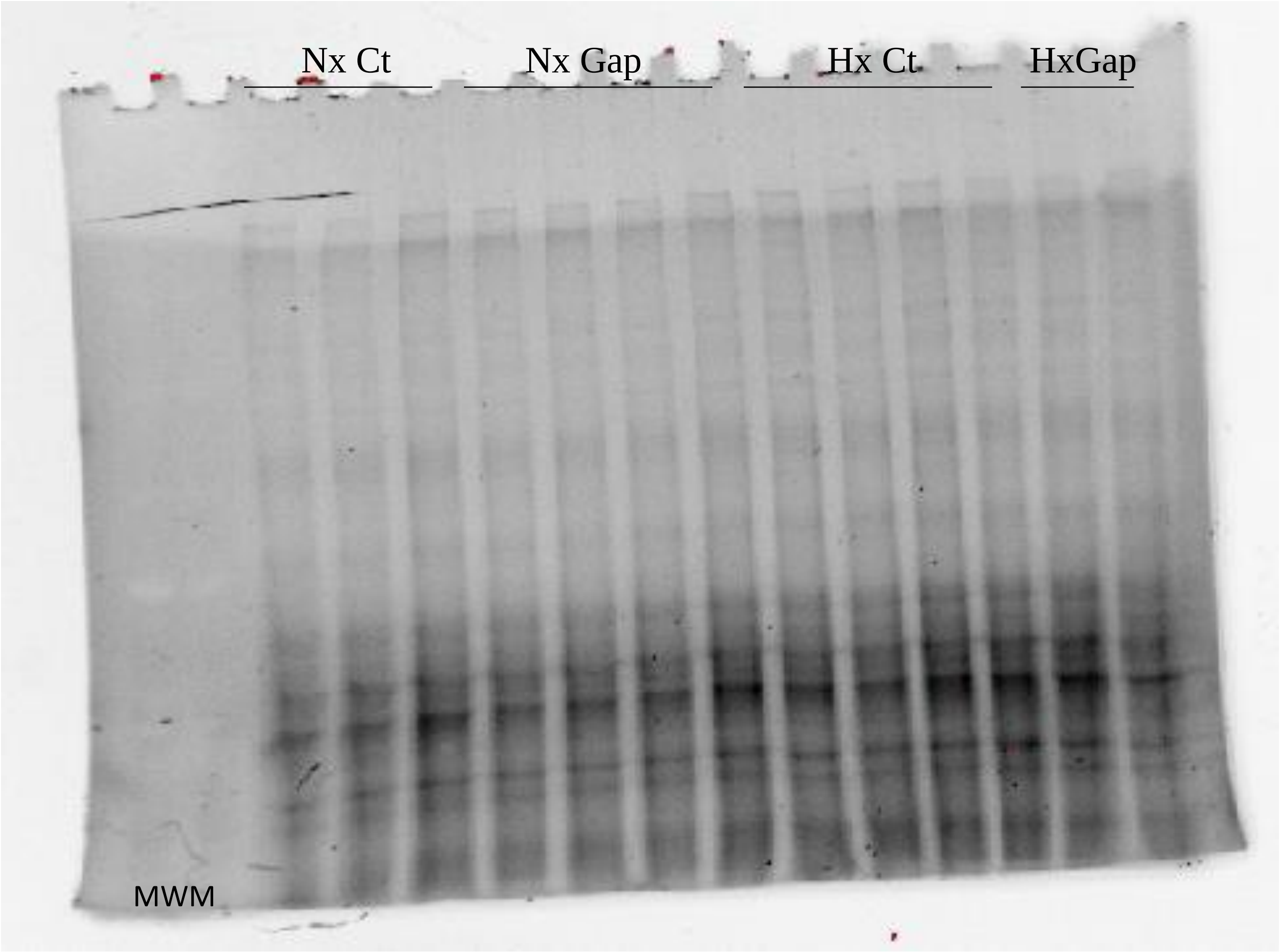

MWM : molecular weight markers

FIGURE S4B

BPD-PH animal model

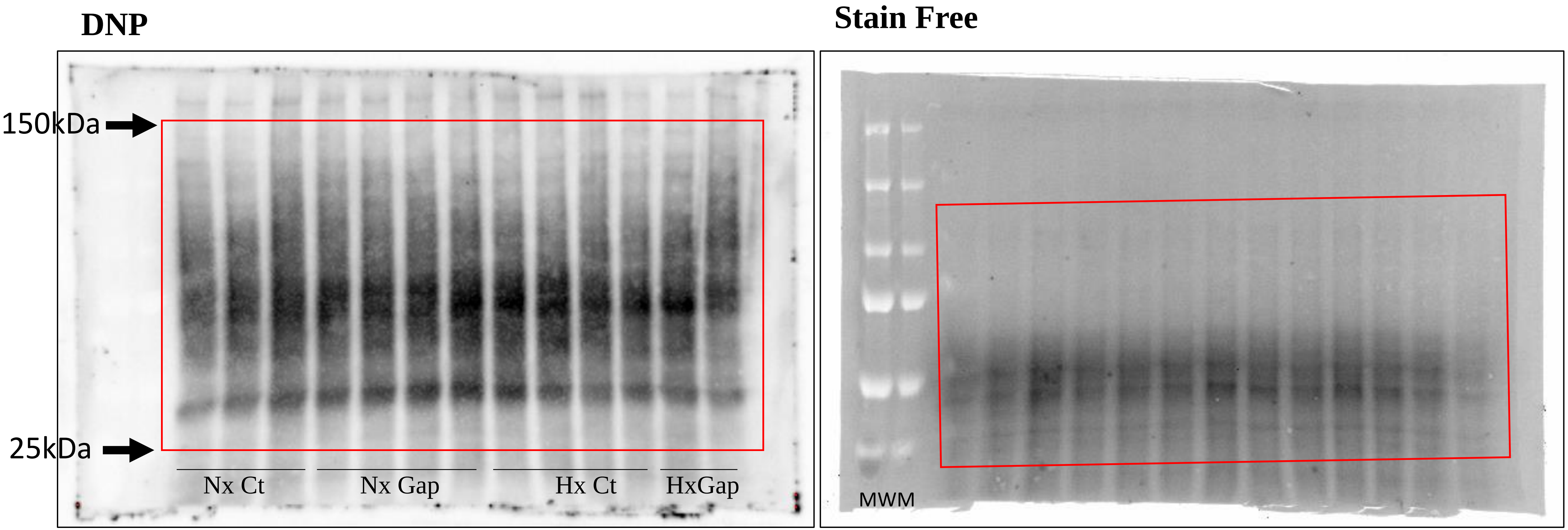

MWM : molecular weight markers

FIGURE S3C

BPD-PH animal model

HO-1 GEL

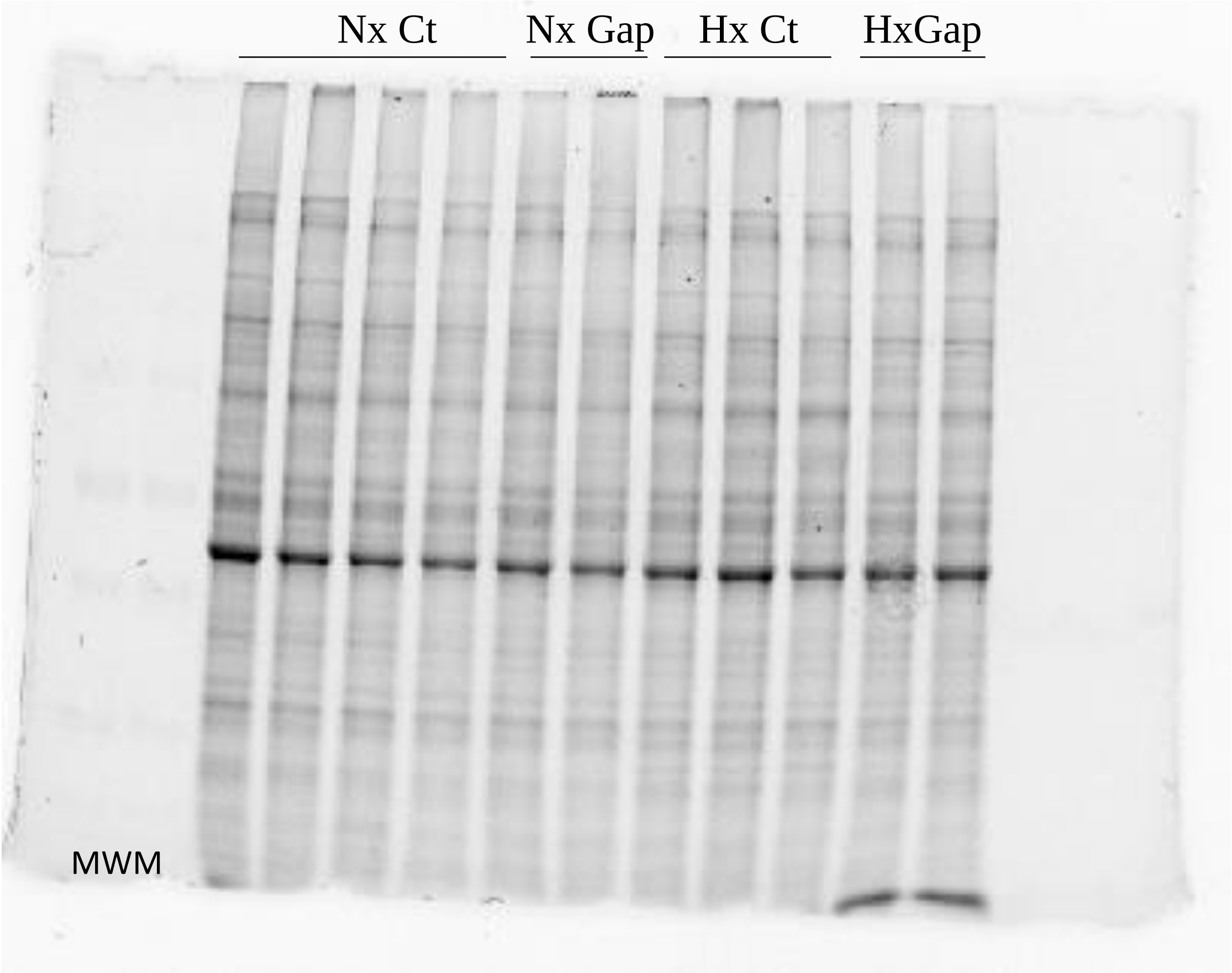

Whole lung

MWM : molecular weight markers

FIGURE S3C

BPD-PH animal model

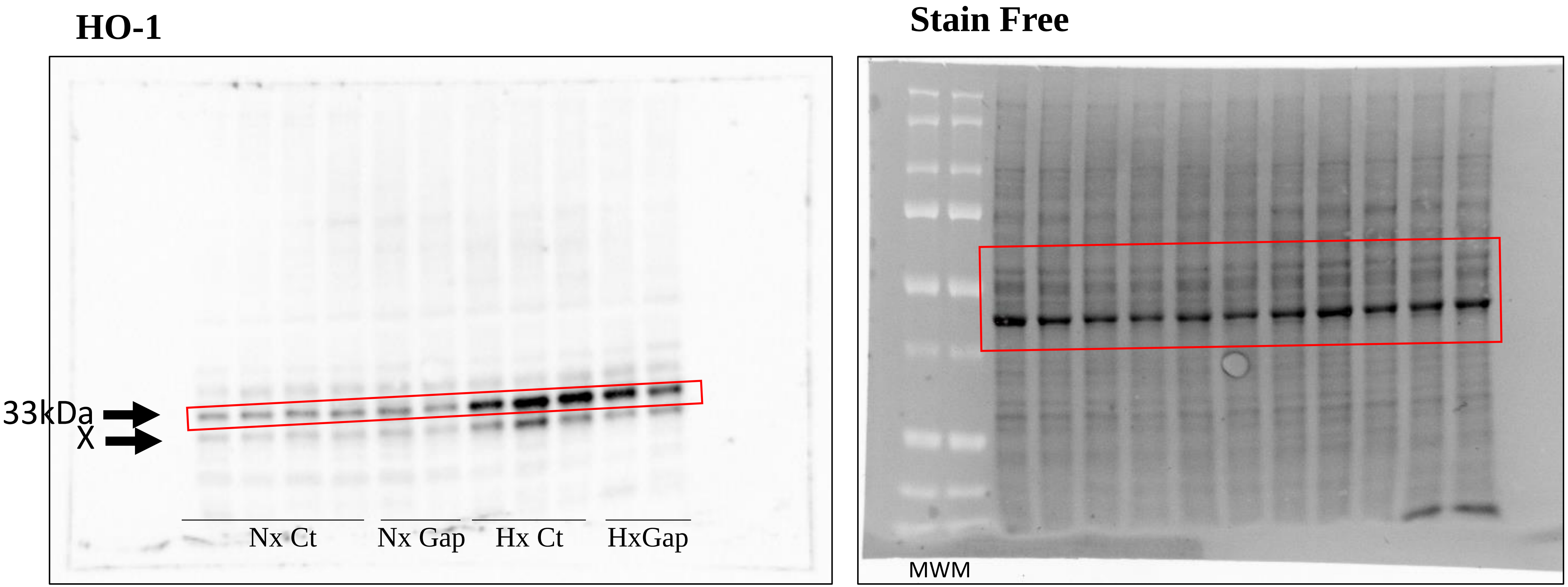

Whole lung

MWM : molecular weight markers

FIGURE S3D

BPD-PH animal model

HO-1 GEL

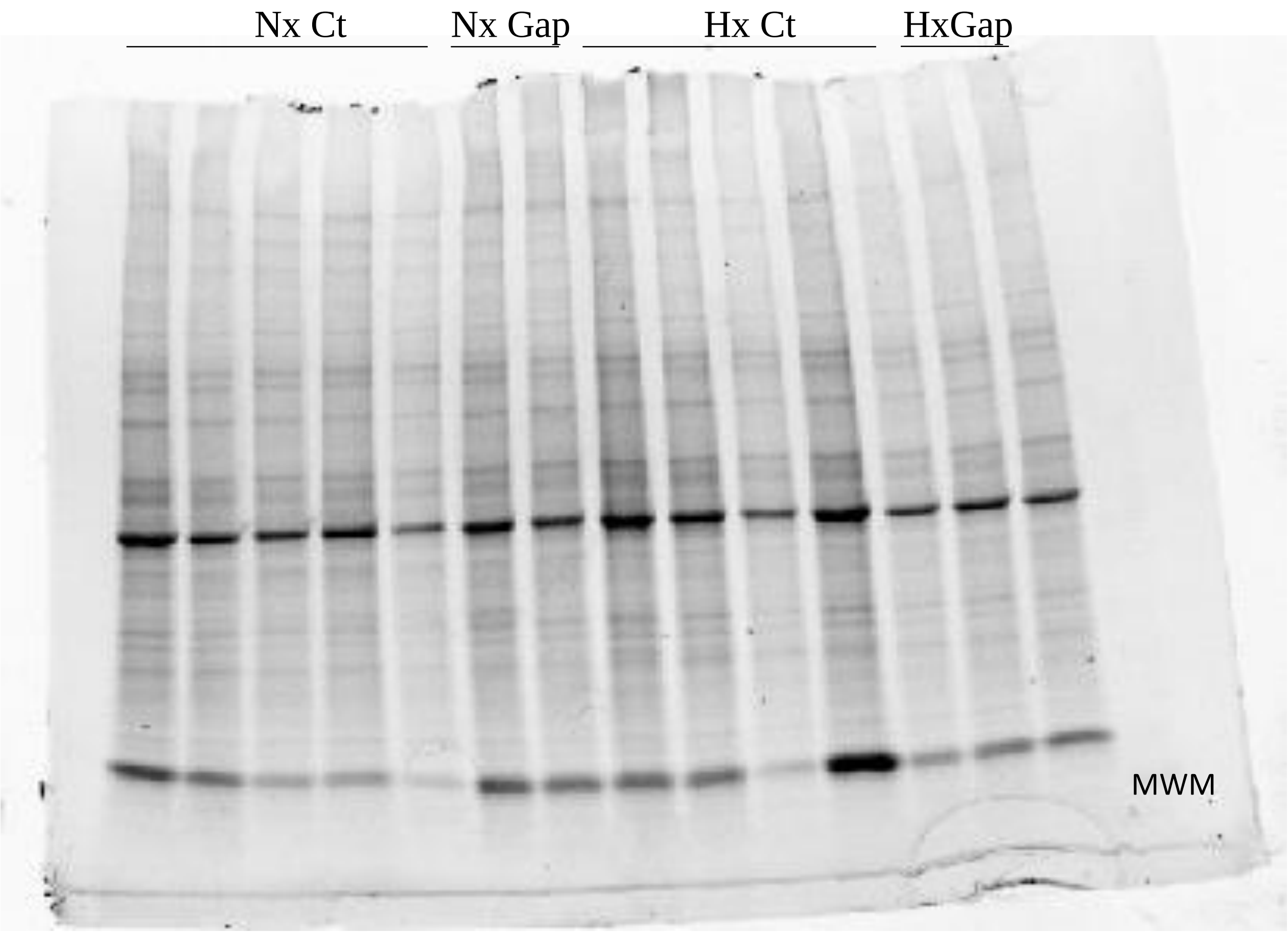

Intrapulmonary arteries

MWM : molecular weight markers

FIGURE S3D

BPD-PH animal model

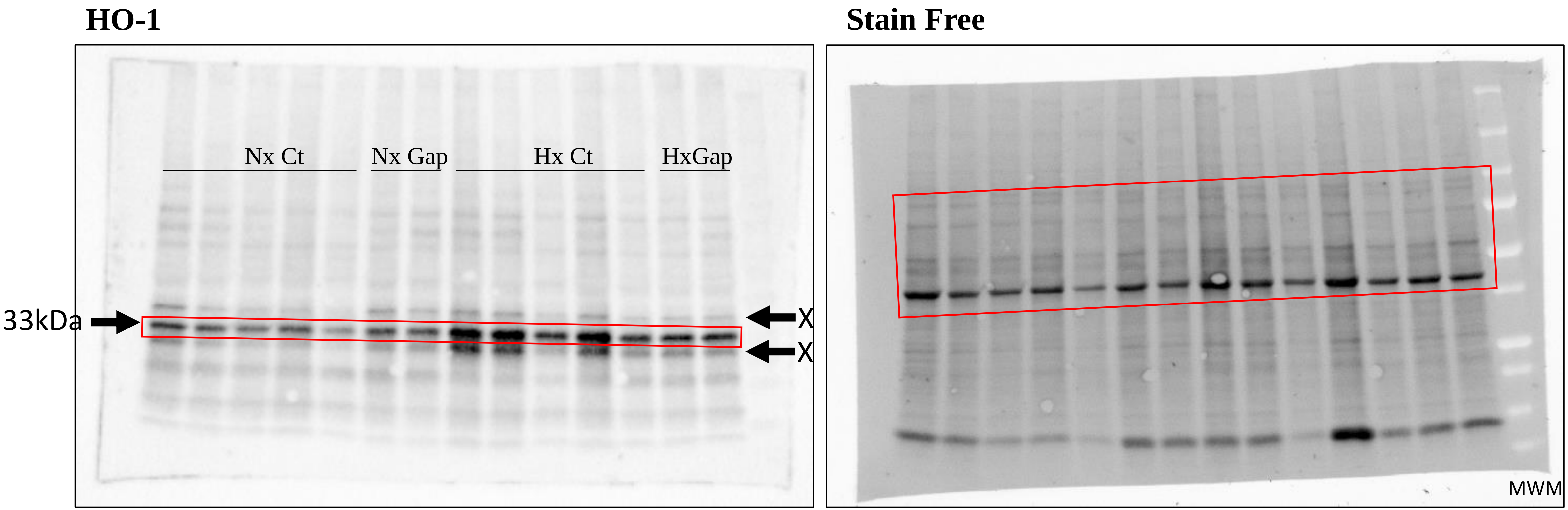

Intrapulmonary arteries

MWM : molecular weight markers

FIGURE S3E

HfPA-SMC

HO-1 GEL

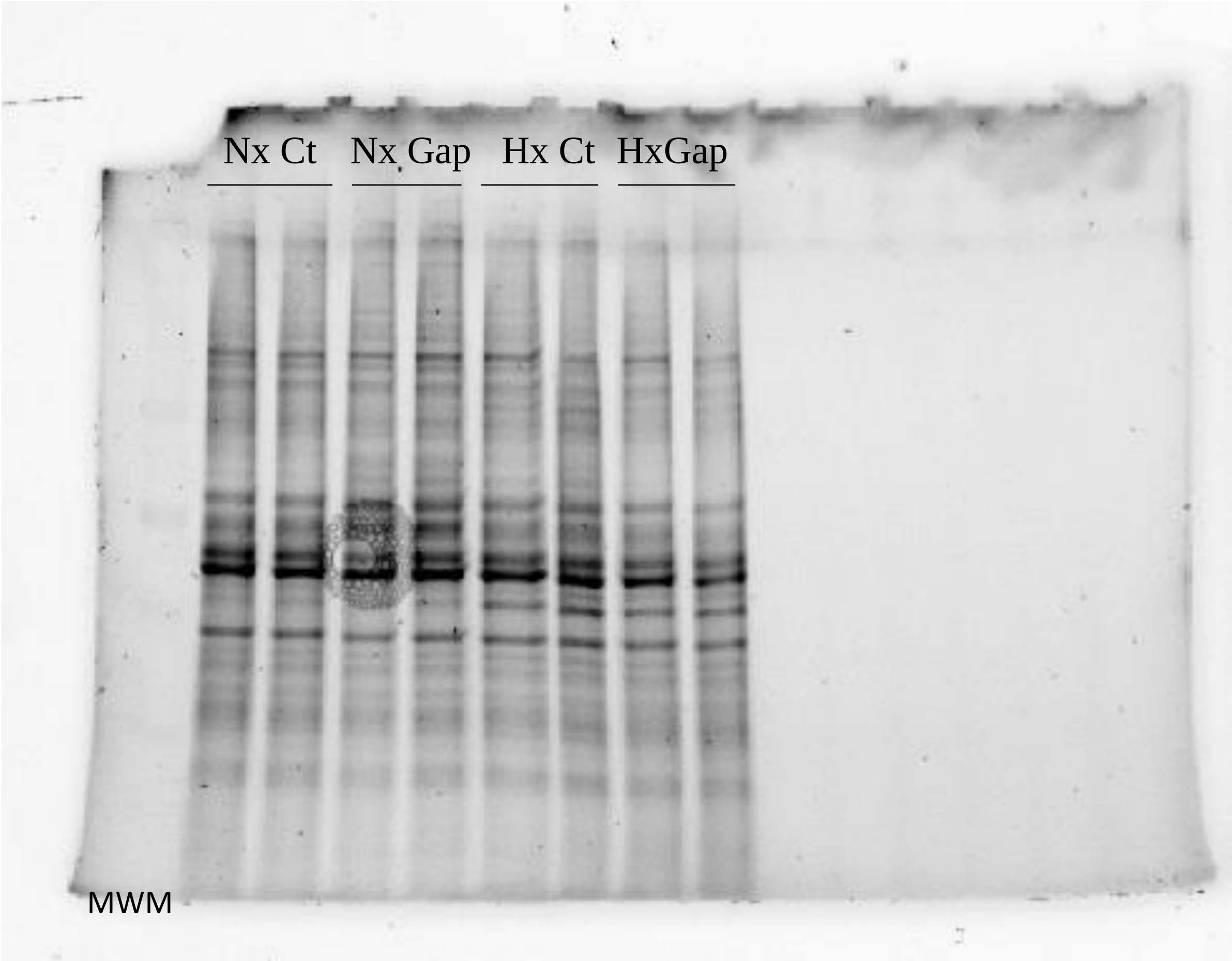

MWM : molecular weight markers

FIGURE S3E

HfPA-SMC

HO-1

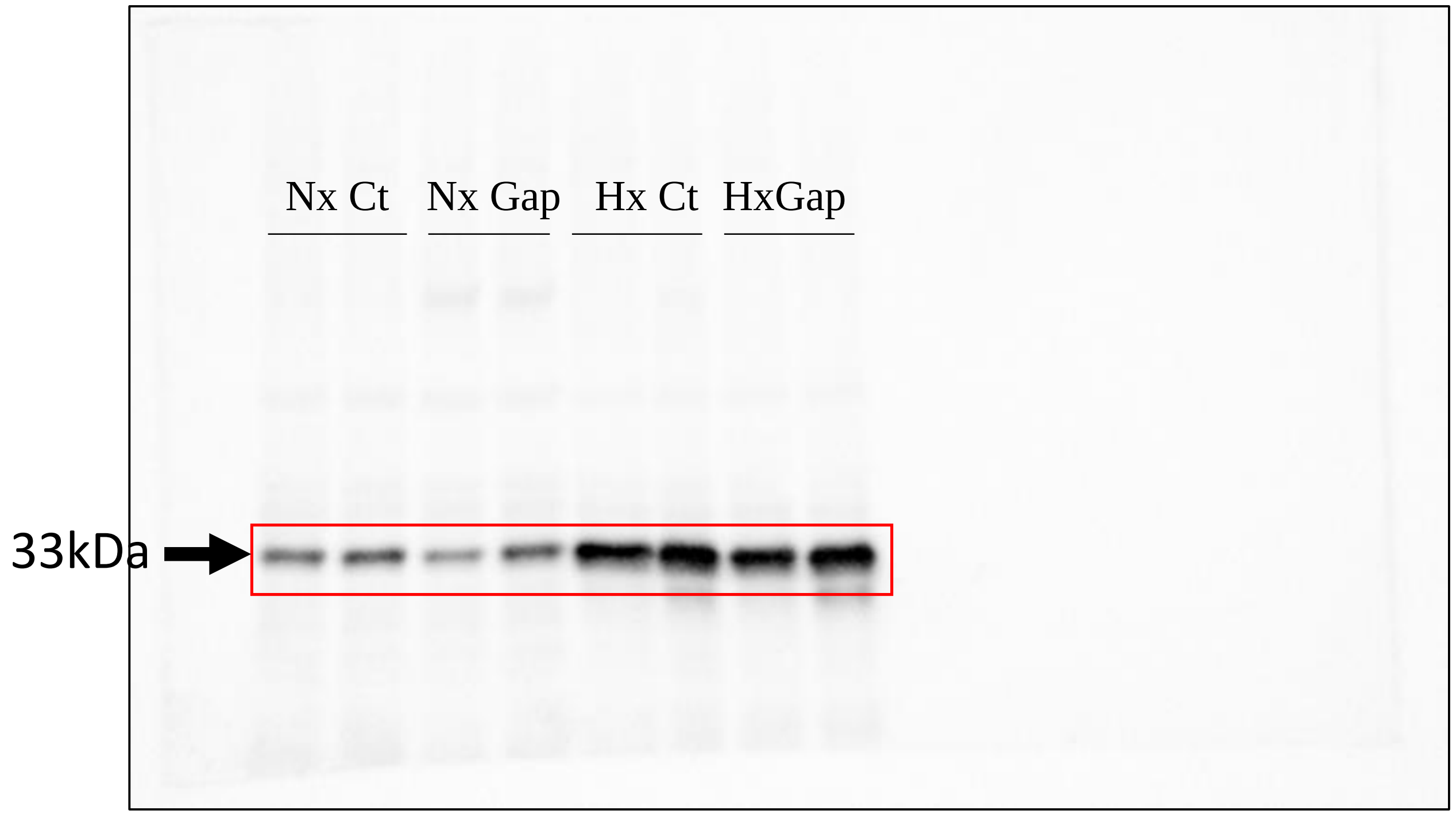

Stain Free

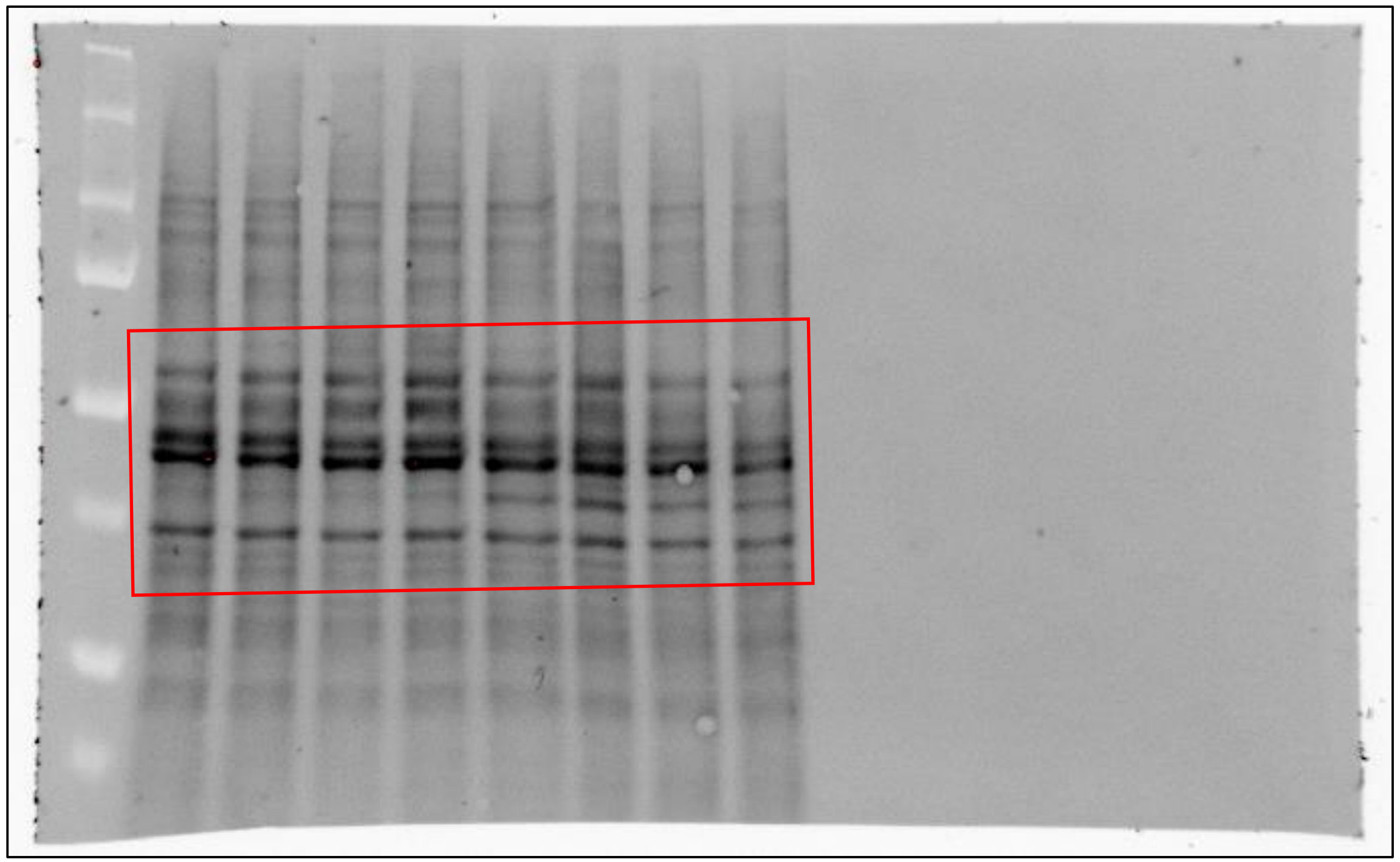

MWM

MWM : molecular weight markers
