# Supplementary material for: Role of connexin 43 in hyperoxia-induced bronchopulmonary dysplasia and associated pulmonary hypertension
Source: PLoS One. 2026 Aug 24;21(8):e0356364. doi: 10.1371/journal.pone.0356364 (PMC13502688; doi:10.1371/journal.pone.0356364)
Supplement: Supplementary file 2 [file pone.0356364.s005.docx]

**Table S1: Primary antibodies used for Western blotting and immunostaining experiments.**

| **Protein** | **Source** | **Reference** | **Reactivity** | **Application** | **Dilution** |
| --- | --- | --- | --- | --- | --- |
| CD68 | Invitrogen | MA5-16654 | Bovine, Rat | Western Blot | 1 :500 |
| CD86 | Clinisciences | NB-22-50271-50 | Human, mouse, rat | Western Blot | 1 :500 |
| CD206 | Abcam | Ab64693 | Human, mouse, rat | Western Blot | 1 :1000 |
| Col1a1* | Abcam | Ab260043 | Human, mouse, rat | Western Blot | 1 :1000 |
| Col3a1^†^ | Abcam | Ab184993 | Human, mouse, rat | Western Blot | 1 :1000 |
| Cx43^‡^ | Sigma-Aldrich | C6219 | Mammals, Human | Western Blot | 1 :5000 |
| DNP^§^ | Merck | S7150 | Species independent | Oxyblot | 1 :150 |
| Fibronectin | Santa Cruz biotechnology | Sc-52331 | Human, mouse, rat | Western Blot | 1 :50 |
| 4-HNE^\|\|^ | Abcam | Ab46545 | Species independent | Western Blot | 1 :500 |
| HO-1^**^ | Proteintech | 66743-1-Ig | Human, mouse, rat | Western Blot | 1 :1000 |
| PCNA^††^ | Abcam | Ab29 | Human, mouse, rat | Immunostaining | 1 :2000 |
| Pro-SP-C^‡‡^ | Sigma-Aldrich | Ab3786 | Mouse, Rat | Western Blot | 1 :500 |
| RT1-40^§§^ | Terrace Biotech | TB-11ART1-40 | Rat | Western Blot | 1 :5000 |
| SP-B^\|\|\|^ | Abcam | Ab231551 | Human, mouse, rat | Western Blot | 1 : 1000 |
| vWF^***^ | Merck | Ab7356-25UG | Human, mouse, rat | Immunostaining | 1 :100 |

^*^Collagen type 1 alpha 1; †Collagen type 3 alpha 1; ^‡^Connexin 43^; §^2,4-Dinitrophenol; ^||^4-Hydroxynonenal; ^**^Heme oxygenase 1; ^††^Proliferating cell nuclear antigen; ^‡‡^ATII cell marker (Pro-surfactant protein C); ^§§^ATI cell marker (Podoplanin); ^|||^ Surfactant protein B; ^***^von Willebrand Factor.
